# Repellency and chemical composition of essential oils from four medicinal plants against *Anopheles arabiensis* (Diptera: Culicidae) under laboratory conditions

**DOI:** 10.1371/journal.pone.0358040

**Published:** 2026-09-15

**Authors:** Zeyede Teshome, Abebe Animut, Belete Adefris Legesse, Mirutse Giday, Esayas Aklilu

**Affiliations:** 1 Department of Biology, Woldia University, Woldia, Ethiopia; 2 Aklilu Lemma Institute of Health Research, Addis Ababa University, Addis Ababa, Ethiopia; 3 Center for Innovative Drug Development and Therapeutic Trials for Africa (CDT-Africa), Addis Ababa University, Addis Ababa, Ethiopia; University of Kashan, IRAN, ISLAMIC REPUBLIC OF

## Abstract

Botanical repellents provide a traditional means of personal protection, yet optimizing their formulations is increasingly required to address shifting mosquito feeding behaviors and mitigate disease transmission. The current study aimed to evaluate the repellent efficacy of essential oils and their binary combinations as complex matrices against *Anopheles arabiensis* bites*,* the major malaria vector in Ethiopia. Essential oils were extracted from fresh parts of four ethnobotanically investigated medicinal plants in the Ghibe Valley, Ethiopia, namely *Croton macrostachyus*, *Echinops kebericho*, *Eucalyptus globulus,* and *Juniperus procera* using hydrodistillation. The chemical composition of the essential oils was analyzed by Gas Chromatography-Mass Spectrometry (GC-MS). Arm-in-cage bioassays using laboratory-reared mosquitoes and human volunteers determined the effective dosage and complete protection time of the essential oils at 10% and 20% concentrations. A total of 131 chemical constituents were identified across the four analyzed essential oils. The *C. macrostachyus* leaf and seed oils provided a significantly longer protection time (*p* < 0.001), reaching 180 minutes with a mean percentage repellency of 85% to 100%. The essential oil of *J. procera* blended with *C. macrostachyus* or *E. kebericho* exhibited significant synergistic repellency (*p* < 0.001). The protection duration demonstrating by *C. macrostachyus* oil underscores their potential to meet the regulatory performance standards for natural insect repellents. Future investigations should include larger sample sizes, dermal toxicity assessments and field validation to fully support the commercialization of these essential oils.

## Introduction

Malaria causes significant public health challenge despite decades of control efforts. It infected over 280 million people and claimed 600,000 lives in 2024 of which over 90% of the cases and deaths were from Africa [1,2]. The disease is the top cause of morbidity and mortality in Ethiopia, driven by a population distribution where approximately 75% of individuals reside in high-endemic areas, 18% in epidemic-prone, and only 7% in low-transmission or malaria-free regions [3–6].

Malaris is transmitted by over 70 *Anopheles* species, of which *Anopheles gambiae* s.s. and *An. arabiensis* are the most effective vectors, while *An. funestus*, *An. bwambae*, *An. merus*, and *An. melas* are involved in localized transmission in sub-Saharan Africa [7,8]. The predominant and most widespread vector in Ethiopia is *An. arabiensis* while *An. nili*, *An. pharoensis*, and *An. funestus* serve as secondary roles in limited settings [9,10]. *An. stephensi*, reported recently, is also a potential vector [11,12].

Vector control remained the primary strategy to combat malaria [13,14]. The use of long-lasting insecticide-treated nets and indoor residual insecticide spraying has been effective to control malaria in indoor settings [13,15,16]. However, these strategies are being challenged by vectors resistance to insecticides [17,18], shift in biting from late-night to early-night and indoor to outdoor [19,20]. This entails the need for alternative control tools including mosquito repellents to suppress vectors regardless of their biting patterns [17,21].

The use of repellents has been acknowledged in vector management [22,23]. Repellents received substantial attention in recent years, largely because of increasing behavioral changes of mosquitoes and outdoor malaria transmission [21]. Repellents remain effective because they cause host-seeking inhibition by temporarily disrupting the mosquitoes’ olfactory responses, which triggers behavioral avoidance by altering their flight orientation, resulting in repellency that prevents host contact [24]. Commercial repellents made from synthetic chemicals like N-diethyl-metatoluamide (DEET), allethrin, N-diethyl mendelic acid amide, and dimethyl phthalate are commonly used for protecting against mosquito bites [22]. However, repeated application of synthetic repellents induces potential health concerns in humans and poses ecotoxicological risks [25–27]. Thus, developing safe and environment friendly alternatives, including from plant extracts, remain a priority [25,28].

Several plant-derived formulations have been validated and commercialized as effective insect repellents [29]. Most notably, essential oils from *Cymbopogon citratus*, *Cymbopogon nardus*, and *Syzygium aromaticum* serve as established botanical standards in vector management [30]. The oils, smokes and tars have also been employed to repel mosquitoes traditionally in Ethiopia [31,32]. Plant based repellents received increased interest in recent years due to their concentrated bioactive phytochemicals [25,33]. Essential oils of plants are complex mixtures of aromatic and chemically pure compounds that convey the unique fragrance and properties of plants that vary greatly by genetics, climate, rainfall and location [34]. Previous studies in Ethiopia demonstrated essential oils of different plants with repellent properties against malaria vectors [35–38]. However, direct comparisons and regulatory evaluations remain challenging due to variations in experimental rigor, such as inconsistent extraction methods and fluctuating mosquito densities per test cage. The current screening study addresses these methodological inconsistencies by implementing standardized protocols for extraction, rigorously adhering to the WHO guidelines, and evaluating selected traditionally used medicinal plants. Our previous ethnobotanical survey in the Ghibe valley, southwest Ethiopia identified and documented several medicinal plants traditionally used to control mosquitoes and other insects [32]. However, scientific evaluation of these ethnobotanically claimed plants against *An. arabiensis* using standardized repellent protocols remained scarce. Blending distinct essential oils can trigger synergistic or antagonistic interactions, altering overall repellent efficacy against mosquitoes [39]. Therefore, the current study investigated the repellent properties of essential oils and their binary combinations extracted from *Croton macrostachyus* Hochst. ex Delile, *Echinops kebericho* Mesfin*, Eucalyptus globulus* Labill. and *Juniperus procera* Hochst. ex Endl. against laboratory reared female *An. arabiensis* bites. A standardized, two-phase experiment involving human volunteers was conducted; phase one determined the minimum effective dosage, while phase two assessed the complete protection time (CPT). Additionally, the chemical composition of the oils was assessed to better understand their efficacy and potential applications in mosquito repellency.

## Materials and methods

### Plant selection and collection

Plants selected on the basis of our previous ethnobotanical survey between March and October, 2024 in Deri Saja Zuria district, Enor, Misha, and Sekoru in the Ghibe Valley, southwest Ethiopia were evaluated [32]. The plants were *C. macrostachyus*, *E. kebericho*, *E. globulus,* and *J. procera* Plant parts were carefully collected in accordance with the local species protection and International Union for Conservation of Nature (IUCN) regulations, with less affected the wild populations in the areas between April and November 2025. Accordingly, fully developed leaves and seeds of *C. macrostachyus* along with leaves of *E. globulus*, were collected from their natural habitat in the Enor district. Leaves of *J. procera* were gathered from natural habitats in the Misha district, while roots of *E. kebericho* were harvested from local farmers in the Deri Saja Zuria district, where they are cultivated and prepared for the local market.

### Essential oil extraction from plant parts

Essential oils were extracted via hydrodistillation using a Clevenger type apparatus. The extraction was conducted at natural product laboratory of Center for Innovative Drug Development and Therapeutic Trials for Africa (CDT Africa), Addis Ababa University (AAU) using two distillation flasks, each with a loading capacity of 600 grams. Fresh plant parts were cut into small pieces and distributed equally between two distillation flasks (600 g of plant material and 3000 mL of water per flask). The distillation chamber was heated at about 80 °C and allowed to boil for more than three hours. The distillate was collected in a separating funnel in which the aqueous portion was separated from the volatile oil. The water layer was slowly drawn off until only the oil layer would remain. The extracted oil was collected in a 10 ml glass bottle and stored at 4 °C up to a maximum of one month prior to chemical analysis and repellency testing.

### Chemical composition analysis of essential oils

Chemical analysis of the essential oils extracted from *E*. *globulus, J*. *procera, C*. *macrostachyus* and *E. kebericho* was performed at the laboratory of natural product chemistry, Adama Science and Technology University via Gas Chromatography-Mass Spectrometry (GC-MS) using an Agilent 7890B gas chromatograph coupled with an Agilent 5977B mass selective detector (Agilent Technologies, Santa Clara, CA, USA). The GC had an HP-5MS column (non-polar column, Agilent Technologies), 30 m × 250 μm internal diameter and 0.25 μm film thickness. The carrier gas was helium flowing at a rate of 1 mL/minute. One μL sample was injected, and the injector temperature was adjusted to 250 °C and the injection mode was split mode with a split ratio of 50:1. The initial oven temperature was programmed from 50 ˚C, held for 1 minute. It was raised to 120 ˚C at 10 ˚C/minute and then ramped by 4˚C/minute to reach 220 ˚C, and finally raised to 280 with the rate of 20 ˚C/minute at this temperature held for 10 minutes. Mass spectra were recorded in EI mode at 70 eV, scanning the 45–550 m/z range.

Data processing and spectral analysis utilized a MassHunter acquisition software workflow. First, the gas chromatograph separated the complex essential oil mixtures into individual components based on their distinct column separation profiles and retention times. Second, the mass spectrometer generated a unique chemical fingerprint for each eluted component through electron ionization and subsequent molecular fragmentation. Mass analysis was conducted using mass-to-charge ratio (m/z) filtering. Compounds were identified by comparing their mass spectra against the National Institute of Standards and Technology (NIST) and Wiley libraries, and by matching their experimental retention indices (RI_Exp_) with literature values (RI_Lit_) [40]. The relative percentage of each chemical constituent was calculated by dividing its individual peak area by the total peak area of all identified compounds and multiplying the result by 100 [41].

### Ethics approval and consent to participate

The Institutional Research Ethics Review Committee (ALIPB-IRERC) of Aklilu Lemma Institute of Health Research, Addis Ababa University (AAU), approved the study and issued an ethical clearance certificate (reference number ALIPB-IRERC/130/2016/24). Written permission letters for field access to all plant collection sites were obtained from the Institute of Public Health in the Central Ethiopia Regional State, as well as from the health office of each study district. Volunteers provided written informed consent on 13 June 2025 after receiving explanations of the procedures and benefits of the study. This consent was obtained prior to conducting each laboratory test of repellency using the arm-in-cage method, which took place from June 15 to November 30, 2025.

### Evaluating repellency of essential oils

The repellency bioassays were performed in accordance with standard WHO guidelines [42]. Each essential oil was diluted in ethanol to prepare a range of serial dilutions at various concentrations. Ethanol evaporates rapidly and serves as a suitable negative control to establish baseline mosquito attraction. Five different (v/v) concentrations in ethanol, such as 10 μL/mL (1%), 25 μL/ mL (2.5%), 50 μL/mL (5%), 75 μL/ mL (7.5%) and 100 μL/mL (10%) were prepared for the dose response testing. Additional ethanolic concentration of 200 μL/mL (20%), were prepared to evaluate the protection duration of each essential oil. Additionally, six binary essential oil combinations (*E. globulus* + *J. procera, E. globulus* + *C. macrostachyus*, *E. globulus* + *E. kebericho, J. procera* + *C. macrostachyus*, *J. procera* + *E. kebericho,* and *C. macrostachyus* + *E. kebericho*) were formulated in a 1:1 (v/v) ratio using micropipettes, followed by shaking to ensure complete homogeneity. These formulations were prepared fresh on the day of testing, stored at room temperature (27 ± 2 °C), and evaluated at concentration of 200 μL/mL (20%) to determine their combined repellent efficacy.

The test mosquitos were reared and maintained in a separate room under optimal environmental conditions of 27 ± 2 °C and 60–70% relative humidity, with constant access to a 10% (w/v) sugar solution. Nulliparous female mosquitoes were starved for 12 hours before the repellent test began. Standardized cages (30 cm × 30 cm × 30 cm) were used for daytime testing in a dark room.

In the current preliminary screening repellency test, a repeated-measures design was utilized to determine both dose-response curves (effective doses) and complete protection time following WHO guideline [42]. This design consisted of five human volunteers (> 30 years old) with no history of allergic reactions to mosquito bites, evaluated across three independent replicates. The protocol produced a total of 15 observational assessments, maintaining acceptable data reliability relative to the conventional sample sizes. This is because it includes differences both within the same person over time and between different people [43]. The volunteers were encouraged to avoid alcohol, tobacco, and any scented products for at least 12 hours before the experiment. The amount of each test oil applied on the arm was determined from the surface area of the arm (in cm^2^) of skin is then calculated according to Eq 1, using the method described by [42].


$$\begin{array}{c} Area\;=\frac{\text{1}}{\text{2}}\left(C_{\text{w}}+\;C_{\text{e}}\right)D_{\text{we}} \end{array}$$ (1)

where *Area* is the total treated surface area of the forearm, $C_{\text{w}}$ is the circumference of the wrist in centimeters, $C_{\text{e}}$ is the elbow-cubital fossa circumference in centimeters, and $D_{\text{we}}$ is the distance between Ce and Cw. Accordingly, the calculated surface of the arm of the exposed volunteers were as follows (V1 = 562.5 cm^2^, V2 = 516 cm^2^, V3 = 588 cm^2^, V4 = 572.5 cm^2^, and V5 = 546.8 cm^2^). Volunteers were required to clean their hands and arms thoroughly followed by drying. A surgical glove was then worn on the hand to prevent biting on the untreated hand. The test area of the exposed volunteer (the inner part of the forearm between the wrist and elbow) was washed with unscented soap and ethanol. Each test consisted of two parts: the right arm was treated with the essential oils, while the left arm served as the control. Each volunteer was exposed to each essential oil once per day. During the time intervals between exposure periods, volunteers were instructed not to rub, touch, or wet the treated arms and were restricted to stay in controlled room to minimize the loss of the oils from the treated forearms.

**Estimation of effective dose:** In this bioassay, a range of doses (1–10%) of all essential oils was tested to determine the effective dosage (ED_50_ and ED_99_). One hundred nulliparous female mosquitoes (5–7 days old) were placed in a cage and allowed to acclimatize for approximately 2 hours. Their biting readiness was then tested by exposing only an ethanol-treated forearm (control arm) for up to 30 seconds. When at least 10 mosquitoes landed on the control arm within 30 seconds, the test continued. The control forearm was removed, and the desired concentration of the oils, with an amount of 1.67 μL/cm^2^, was applied uniformly on the treatment arms of the volunteers using a pipetting and painting method to ensure precise dosage control. Cumulative dosing was chosen following the WHO guidelines because it allows for the precise determination of dose-response curve using a small number of human volunteers. To control the influence of previous applications, each new dose was added to the oil already present on the skin to reach the target concentration. Also, the 30-second exposure time was too short to alter the mosquitoes’ behavior. To prevent the oils from evaporating between applications, the doses were applied at fixed and short time intervals. In addition, evaporation was controlled by performing all tests under constant, controlled laboratory room conditions as well as volunteers were restricted in the laboratory room for the entire experiment to ensure a stable physiological condition. The successive doses of oils were tested one after the other starting from the lowest concentration to the highest, until no landing was recorded. The repellent dose for each test was calculated as the sum of the doses applied, resulting in a cumulative dose for each test [42]. Each test concentration was repeated 3 times per volunteer in separate days using fresh mosquitoes. The number of mosquito landing was recorded and used in data analysis.

**Duration of mosquito landing protection:** in this bioassay, 10% concentrations of each essential oil was selected based on the preliminary dose-response testing and established literature [44–46]. This concentration was selected to exceed calculated baseline ED_99_ values, compensating for the high volatile nature of essential oils. Additionally, a 20% concentration of each essential oil and their binary blends was selected in accordance with WHO guidelines, to allow a direct comparative analysis against a 20% standard DEET positive control [42]. These two concentrations were evaluated to establish a uniform baseline for all five essential oils and their binary blends, as well as to ensure a sufficient chemical reservoir on the skin for measuring protection time. For each treatment, 200 nulliparous female mosquitoes (5–7 days old) were released into the test cage and allowed to acclimatize for 2 hours. An additional 200 nulliparous females were released in a separate cage for the control group to determine the protection time of each individual essential oil and their binary combinations. Following the application of each treatment group at a standardized concentration of 1.67 μL/cm^2^, the arms were initially exposed at 30 minutes, with subsequent exposures continued at specified time intervals. The exposure was made for 3 minutes, then withdrawn for 30 minutes, and then exposed again. Any mosquito landing and biting were counted and scored. The bioassay was terminated upon the landing of a second consecutive mosquito during a single exposure period, with this duration recorded as the complete-protection time. The testing period lasted up to 6 hours for each treatment session, depending on the efficacy. Three independent replicates were conducted for each concentration on separate days using fresh mosquitoes, with a minimum inter-session interval of 7 days between sessions. The percentage of repellency was calculated according to Eq 2, using the method described by [47].


$$\begin{array}{c} \%\text{ Repellency }=\frac{C\;-\;T}{C}\text{x 100} \end{array}$$ (2)

where *C* is number of mosquitoes landing on control forearm, and *T* is the number of mosquitoes landing on the treated forearm.

### Statistical analysis

The Statistical Package for the Social Sciences (SPSS) computer software version 25 was used for analyzing the data. The median effective dosage (ED_50_) and ED_99_ of the essential oils were determined by probit plane regression analysis. Effectiveness of the test was determined by comparing the 95% confidence intervals of the ED_50_ and ED_99_ values. The complete protection time (CPT) for a given dose was estimated from the time elapsed up to the first confirmed mosquito landing and/or probing within 30 minutes. The median CPT and its confidence interval were estimated using the Kaplan–Meier survivor function procedure. After a non-parametric Kruskal-Wallis H test of significant differences, Dunn’s post hoc test was conducted to identify specific differences in the median protection time for each oil, as well as between the protection times of single and combined oils.

## Results

### Yield of essential oils extracted from plant parts

The essential oil yields of the extracted plant parts are summarized in Table 1. The highest yield was obtained from *E. globulus* leaves at 1.50% (18.00 mL/ 1200 g), followed by *J. procera* leaves at 0.23% (2.75 mL/1200 g), and *C. macrostachyus* seeds at 0.18% (2.20 mL/1200 g). Conversely, the lowest volume was obtained from the leaves of *C. macrostachyus*, which yielded 0.02% (0.25 mL/1200 g).

**Table 1:** Essential oil yield (%) of five distinct parts collected from four medicinal plants in the Ghibe Valley, Ethiopia, 2025.

Scientific name	Family name	Local name (Language)	Location coordinates	Parts used	Frequency (%, n = 361)	Voucher number	Yield (%)
*Croton macrostachyus*	Euphorbiaceae	Wanshehna (Guragegna)	8.87868N 37.2312E	Leaves Seeds	81	Z-13–2024	0.02 0.18
*Echinops kebericho*	Asteraceae	Kebercho (Amharic)	7.55885N 37.2803E	Roots	58	Z-37–2024	0.17
*Eucalyptus globulus*	Myrtaceae	Nech Bahir Zaf (Amharic)	8.87925N 37.2384E	Leaves	56	Z-03–2024	1.50
*Juniperus procera*	Cuperssaceae	Yehabesh Tsid (Amharic)	7.69839N 37.7314E	Leaves	63	Z-09–2024	0.23

### Chemical composition of essential oils

A total of 131 chemical compounds were identified across the four analyzed essential oils. The chemical composition of the essential oils, including literature retention index (RI_Lit_), experimental retention index (RI_Exp_), retention time (RT), compound name, registry number (CAS), and peak areas are presented in Tables 2–5.

**Table 2:** Chemical composition of essential oil from the seeds of *Croton macrostachyus*, 2025.

NO	RI_Lit_ [40,48–50]	RI_Exp_	RT	Compound name	CAS	Content (%)
1	977.7	964.9	6.05	β-Pinene	127-91-3	0.85
2	1099.0	1104.0	7.916	Linalool	78-70-6	2.45
3	1337.0	1339.0	12.35	**Cyclohexene, 4-ethenyl-4-methyl-3-(1-methylethenyl)-1-(1-methylethyl)-, (3R-trans)-**	20307-84-0	0.72
4	1351.4	1354.3	12.62	α-Cubebene	17699-14-8	1.37
5	1499.0	1499.0	12.74	Aciphyllene	87745-31-1	1.00
6	1376.2	1394.1	13.27	Copaene	3856-25-5	0.47
**7**	**1440.6**	**1440.0**	**13.58**	**Aromadendrene**	**489-39-4**	**4.13**
8	1391.0	1391.7	13.85	(1S,5S)-2-Methyl-5-((R)-6-methylhept-5-en-2-yl) bicyclo[3.1.0]hex-2-ene	159407-35-9	0.48
**9**	**1433.1**	**1433.0**	**14.5**	**beta-Copaene**	**18252-44-3**	**6.25**
10	1435.0	1437.2	14.8	(1R,3aS,8aS)-7-Isopropyl-1,4-dimethyl-1,2,3,3a,6,8a-hexahydroazulene	36577-33-0	0.63
11	1508.4	1506,0	15.11	β-Bisabolene	495-61-4	0.47
**12**	**1480.6**	**1480.4**	**15.91**	**(-)-Germacrene D**	**23986-74-5**	**4.68**
**13**	**1476.2**	**1474.0**	**16.17**	**γ-Muurolene**	**30021-74-0**	**29.33**
14	1517.9	1497.0	16.26	α-Muurolene	10208-80-7	2.45
15	1504.1	1507.0	16.37	α-Farnesene	502-61-4	2.46
**16**	**1513.1**	**1511.2**	**16.64**	**γ-Cadinene**	**39029-41-9**	**4.32**
**17**	**1523.2**	**1519.0**	**16.88**	**Naphthalene, 1,2,3,5,6,8a-hexahydro-4,7-dimethyl-1-(1-methylethyl)-, (1S-cis)-**	**483-76-1**	**4.15**
18	1532.0	1529.3	17.05	Naphthalene, 1,2,3,4,4a,7-hexahydro-1,6-dimethyl-4-(1-methylethyl)-	16728-99-7	0.42
19	1544.0	1546.2	17.17	Naphthalene, 1,2,4a,5,6,8a-hexahydro-4,7-dimethyl-1-(1-methylethyl)-, [1S-(1α,4aβ,8aα)]-	24406-05-1	1.36
20	1565.6	1571.0	17.73	1,6,10-Dodecatrien-3-ol, 3,7,11-trimethyl-, [S-(Z)]-	7212-44-4	2.33
21	1514.0	1507.0	18.14	Cubebol	23445-02-5	1.35
22	1694.5	1692.8	18.39	(1R,7S, E)-7-Isopropyl-4,10-dimethylenecyclodec-5-enol	81968-62-9	0.54
23	1494.1	1495.0	19.02	(1S,2E,6E,10R)-3,7,11,11-Tetramethylbicyclo [8.1.0] undeca-2,6-diene	24703-35-3	0.31
24	1637.8	1639.1	19.77	τ-Cadinol	5937-11-1	1.21
25	1640.0	1638.9	20.11	τ-Muurolol	19912-62-0	1.52
26	1510.0	1510.0	21.45	Tridecanal	10486-19-8	0.31
**27**	**1761.3**	**1753.0**	**23.00**	**Benzyl Benzoate**	**120-51-4**	**13.24**
28	1822.0	1820.7	24.00	Hexadecanal	629-80-1	0.37
29	1883.0	1881.3	25.58	Cyclohexadecane P489	295-65-8	0.39
30	1964.7	1967.0	28.15	Trachylobane	5282-35-9	0.32

**Table 3:** Chemical composition of essential oil from the roots of *Echinops kebericho*, 2025.

NO	RI_Lit_ [40,48–50]	RI_Exp_	RT	Compound name	CAS	Content (%)
1	1004.1	1007.2	5.26	α-Phellandrene	99-83-2	0.70
**2**	**1011.3**	**1005.0**	**5.40**	**3-Carene**	**13466-78-9**	**4.56**
**3**	**977.7**	964.9	**6.08**	β-Pinene	**127-91-3**	**4.20**
4	989.2	981.0	6.21	β-Myrcene	123-35-3	0.18
5	1103.0	1106.3	6.77	Cymene	25155-15-1	0.15
6	1362.9	1365.0	6.86	2,6-Octadien-1-ol, 3,7-dimethyl-, acetate, (E)-	141-12-8	2.01
7	1059.7	1060.0	7.30	γ-Terpinene	99-85-4	0.55
8	1097.0	1097.0	7.77	4-Isopropylidene-1-cyclohexene	586-62-9	0.19
9	950.3	951.0	7.91	Camphene	79-92-5	0.22
10	1144.4	1133.5	8.70	cis-Verbenol	1845-30-3	0.59
11	1166.6	1170.0	9.04	p-Mentha-1,5-dien-8-ol	1686-20-0	0.41
12	1177.1	1177.0	9.24	Terpinen-4-ol	562-74-3	0.39
13	1280.0	1281.2	11.28	1,7,7-Trimethylbicyclo [2.2.1] hept-2-yl acetate	5655-61-8	1.95
14	1330.7	1330.0	12.14	Silphiperfol-5-ene	138752-24-6	0.85
15	1368.2	1364.0	13.06	(+)-Cycloisosativene	22469-52-9	2.08
**16**	**1392.3**	**1389.0**	**13.47**	**Modephene**	**68269-87-4**	**13.59**
17	1382.7	1365.8	13.63	alpha -Isocomene	65372-78-3	0.54
**18**	**1401.8**	**1399.0**	**13.79**	**Methyleugenol**	**93-15-2**	**6.12**
19	1412.7	1410.9	14.10	(1R,3aS,5aS,8aR)-1,3a,5a-Trimethyl-4-methylenedecahydrocyclopenta[c]pentalene	71596-72-0	2.84
20	1440.6	1440.0	14.33	Aromadendrene	489-39-4	0.17
21	1435.0	1433.6	14.80	(1R,3aS,8aS)-7-Isopropyl-1,4-dimethyl-1,2,3,3a,6,8a-hexahydroazulene	36577-33-0	0.14
22	1408.6	1408.0	15.59	alpha. -Gurjunene	489-40-7	0.62
23	1485.9	1469.0	15.85	trans-β-Ionone	79-77-6	2.93
24	1486.1	1509.0	15.93	Naphthalene, decahydro-4a-methyl-1-methylene-7-(1-methylethenyl)-, [4aR-(4aα,7α,8aβ)]-	17066-67-0	2.12
**25**	**1523.2**	**1519.0**	**16.41**	**Naphthalene, 1,2,3,5,6,8a-hexahydro-4,7-dimethyl-1-(1-methylethyl)-, (1S-cis)-**	**483-76-1**	**13.80**
**26**	**1534.5**	**1532.0**	**16.68**	**(3S,6S)-6-Isopropyl-3-methyl-2-(propan-2-ylidene)-3-vinylcyclohexanone**	**21698-46-4**	**4.90**
27	1472.2	1479.0	18.14	1.beta.,4. beta.H,10. beta.H-Guaia-5,11-diene	22567-17-5	2.47
28	1580.6	1578.1	18.38	Caryophyllene oxide	1139-30-6	0.20
29	1616.6	1613.4	18.86	1H-Benzocyclohepten-7-ol, 2,3,4,4a,5,6,7,8-octahydro-1,1,4a,7-tetramethyl-, cis-	6892-80-4	0.84
30	1637.8	1639.0	19.76	τ-Cadinol	5937-11-1	0.30
31	1420.1	1417.0	20.00	2,6,10,10-Tetramethylbicyclo [7.2.0] undeca-1,6-diene	87-44-5	2.24
32	1651.9	1650.0	20.12	α-Cadinol	481-34-5	1.87
33	1774.2	1769.6	20.29	2-((2R,4aR,8aS)-4a-Methyl-8-methylenedecahydronaphthalen-2-yl) prop-2-en-1-ol	515-20-8	0.86
34	1758.7	1763.0	20.86	7-Isopropenyl-1,4a-dimethyl-4,4a,5,6,7,8-hexahydro-3H-naphthalen-2-one	473-08-5	0.44
35	1695.4	1701.2	20.95	alpha-Costic aldehyde	4586-01-0	0.25
36	1507.7	1507.0	24.09	(1S,7S,8aR)-1,8a-Dimethyl-7-(prop-1-en-2-yl)-1,2,3,7,8,8a-hexahydronaphthalene	190327-38-9	0.43
37	1953.9	1953.0	27.13	Dihydrodehydrocostus lactone	4955-03-7	0.51
**38**	2006.7	2007.2	**28.66**	**Dehydrocostus lactone**	**477-43-0**	**15.74**

**Table 4:** Chemical composition of the essential oil from the leaves of *Eucalyptus globulus*, 2025.

NO	RI_Lit_ [40,48–50]	RI_Exp_	RT	Compound name	CAS	Content (%)
**1**	**1011.3**	**1005.0**	**5.42**	**3-Carene**	**13466-78-9**	**16.85**
2	977.7	964.9	6.05	β-Pinene	127-91-3	0.50
3	989.2	981.0	6.22	β-Myrcene	123-35-3	1.33
4	1004.1	1007.0	6.47	α-Phellandrene	99-83-2	0.32
5	1103.0	1106.3	6.78	Cymene	25155-15-1	0.22
**6**	**1031.8**	**1038.0**	**7.00**	**Eucalyptol**	**470-82-6**	**42.50**
7	1059.7	1064.0	7.32	γ-Terpinene	99-85-4	0.36
8	1093.0	1093.5	7.77	4-Isopropylidene-1-cyclohexene	586-62-9	0.55
9	1177.1	1177.0	9.24	Terpinen-4-ol	562-74-3	1.20
10	1189.7	1190.0	9.48	α-Terpineol	98-55-5	2.57
11	1254.9	1267.0	10.56	2,6-Octadien-1-ol, 3,7-dimethyl-, (E)-	106-24-1	0.63
12	1345.5	1343.6	12.43	2-Oxabicyclo [2.2.2] octan-6-ol, 1,3,3-trimethyl-, acetate	57709-95-2	0.24
**13**	**1347.0**	**1367.0**	**12.64**	**α-Terpinyl acetate**	**80-26-2**	**13.63**
14	1408.6	1408.0	14.04	. alpha. -Gurjunene	489-40-7	0.94
15	1440.6	1440.0	14.75	Aromandendrene	489-39-4	2.93
16	1576.4	1571.0	18.17	(+)-Spathulenol	6750-60-3	0.31
**17**	**1581.8**	**1580.0**	**18.38**	(-)-Globulol	**489-41-8**	**6.06**
18	1650.1	1645.0	19.3	β-Eudesmol	473-15-4	0.93
19	1804.5	1799.7	19.73	Tau-Cadinol acetate	149197-48-8	0.27
20	1651.7	1652.0	20.08	α-Eudesmol	473-16-5	0.28

**Table 5:** Chemical composition of essential oil from the leaves of *Juniperus procera*, 2025.

NO	RI_Lit_ [40,48–50]	RI_Exp_	RT	Compound name	CAS	Content (%)
1	1004.1	1007.7	5.264	α-Phellandrene	99-83-2	0.40
2	954.0	948.7	5.594	(+)-Camphene	5794-03-6	2.52
3	1022.0	1023.0	5.952	β-Cymene	535-77-3	0.32
**4**	**977.7**	964.9	**6.056**	β-Pinene	**127-91-3**	**4.66**
**5**	**989.2**	**981.0**	**6.217**	**β-Myrcene**	**123-35-3**	**3.49**
**6**	**1011.3**	**1005.0**	**6.616**	**3-Carene**	**13466-78-9**	**35.09**
7	1030.0	1026.0	6.789	β-Phellandrene	555-10-2	0.64
**8**	**973.0**	**973.0**	**6.864**	**4(10)-Thujene**	**3387-41-5**	**3.31**
9	1059.7	1060.0	7.297	γ-Terpinene	99-85-4	0.22
**10**	**1086.9**	1097.0	**7.777**	**4-Isopropylidene-1-cyclohexene**	**586-62-9**	**6.23**
11	1099.0	1104.0	7.915	Linalool	78-70-6	2.15
12	1144.2	1144.0	8.528	trans-Verbenol	1820-09-3	0.54
13	1143.4	1445.0	8.718	Camphor	76-22-2	0.62
14	1153.0	1149.8	9.042	(-)-(Z)-Verbenol	18881-04-4	0.21
15	1177.1	1177.0	9.232	Terpinen-4-ol	562-74-3	0.80
16	1189.7	1190.0	9.457	α-Terpineol	98-55-5	0.43
17	1226.7	1220.0	9.798	cis-Carveol	1197-06-4	0.29
18	1285.0	1280.0	11.26	1,7,7-Trimethylbicyclo [2.2.1] hept-2-yl acetate	5655-61-8	1.30
19	1317.6	1318.0	11.82	2,4-Decadienal, (E, E)-	25152-84-5	0.17
**20**	**1420.1**	**1417.0**	**14.29**	**Caryophyllene**	**87-44-5**	**5.64**
21	1476.2	1474.0	15.59	γ-Muurolene	30021-74-0	0.56
22	1486.1	1509.0	15.87	Naphthalene, decahydro-4a-methyl-1-methylene-7-(1-methylethenyl)-, [4aR-(4aα,7α,8aβ)]-	17066-67-0	0.18
23	1440.6	1440.0	16.04	Aromandendrene	489-39-4	0.21
24	1513.1	1511.0	16.53	γ-Cadinene	39029-41-9	0.31
25	1547.6	1544.0	16.64	α-Copaen-11-ol	41370-56-3	0.18
26	1523.2	1519.0	16.73	Naphthalene, 1,2,3,5,6,8a-hexahydro-4,7-dimethyl-1-(1-methylethyl)-, (1S-cis)-	483-76-1	0.88
27	1435.0	1433.6	17.08	Guaia-6,9-diene	36577-33-0	0.35
28	1547.5	1537.0	17.39	Elemol	639-99-6	1.24
29	1694.5	1693.6	17.54	(1R,7S, E)-7-Isopropyl-4,10-dimethylenecyclodec-5-enol	81968-62-9	0.20
30	1788.0	1784.7	17.62	2-((2R,4aR,8aS)-4a-Methyl-8-methylenedecahydronaphthalen-2-yl) prop-2-en-1-ol	515-20-8	0.18
31	1580.6	1578.0	18.32	Caryophyllene oxide	1139-30-6	0.62
32	1604.7	1606.0	18.98	(1R,3E,7E,11R)-1,5,5,8-Tetramethyl-12-oxabicyclo [9.1.0] dodeca-3,7-diene	19888-34-7	0.46
33	1630.9	1635.0	19.5	gamma-eudesmol	1209-71-8	0.58
34	1650.1	1645.0	20	beta-eudesmol	473-15-4	0.16
35	1651.7	1669.3	20.07	alpha-eudesmol	473-16-5	1.90
**36**	**1993.1**	**1989.0**	**28.37**	**13-epi-manoyl oxide**	**596-84-9**	**12.41**
37	20054.0	20054.2	29.78	Dehydroabietane	19407-28-4	0.18
38	2080.5	2080.0	30.37	decahydrophenanthrene	35241-40-8	1.78
39	2079.3	2082.0	30.9	Kolavelool	19941-81-2	0.54
40	1948.0	1939.9	31.71	(+)-Cembrene	1898-13-1	0.51
41	2213.6	2211.8	32.63	Sandaracopimaral	3855-14-9	0.31
42	2303.0	2298.7	34.62	trans-Totarol, Podocarpa-8,11,13-trien-13-ol, 14-isopropyl-	511-15-9	1.04
43	2325.0	2326.2	34.82	Ferruginol	514-62-5	0.18

The essential oil extracted from the seeds of *C. macrostachyus* contained a total of 30 chemical constituents, accounting for 89.88% of the overall chemical composition. The predominant compound identified was γ-Muurolene followed by Benzoic acid-phenylmethyl ester, which comprised 29.33% and 13.24% of the total mixture, respectively (Table 2).

A total of 38 chemical constituents, representing 91.95% of the total chemical composition of essential oil extracted from *E. kebericho*, were identified. The most abundant compound identified from this oil was Dehydrocostus lactone (15.74%) followed by Naphthalene, 1,2,3,5,6,8a-hexahydro-4,7-dimethyl-1-(1-methylethyl)-, (1S-cis)- (13.80%) and Modephene (13.59%) (Table 3).

In the essential oil of *E*. *globulus*, 20 chemical constituents representing 92.62% of the total chemical composition were identified. The major constituents of this essential oil are Eucalyptol (42.50%) followed by 3-carene (16.85%) and α-Terpinyl acetate (13.63%) (Table 4).

In total, 43 chemical constituents, accounting for 93.99% of the total chemical composition of *J. procera* essential oil, were identified. It mainly contained 3-carene (35.09%) followed by 13-epi-manoyl oxide (12.41%) (Table 5).

### Dose-dependent repellent effects of essential oils

The five essential oils, applied at a uniform rate of 1.67 μL/cm^2^, demonstrated varying degrees of repellent activity against *An. arabiensis* as shown in Table 6. The essential oils obtained from *C. macrostachyus* leaves and seeds exhibited the lowest ED_50_ values (0.45%; 95% CI: 0.39–0.51 and 0.48%; 95% CI: 0.41–0.53, respectively) and ED_99_ values (2.48%; 95% CI: 2.29–2.73 and 2.50%; 95% CI: 2.31–2.77, respectively). Conversely, the essential oil obtained from *J. procera* demonstrated the weakest repellent activity, yielding the highest ED_50_ (0.97%; 95% CI: 0.92–1.06) and ED_99_ (4.83%; 95% CI: 4.55–5.16) values.

**Table 6:** The effective dosages required to repel 50% (ED_50_) and 99% (ED_99_) of the mosquitoes for five essential oils against laboratory-reared *Anopheles arabiensis*, 2025.

Essential oils	(*B* ± *SE*)	Z value (*p*)	ED_50_ (95%CI)	ED_99_ (95%CI)	X^2^ (df = 3)	*p*-value
*C. macrostachyus* (leaves)	3.14 ± 0.18	17.54 (<.001)	0.45 (0.39–0.51)	2.48 (2.29–2.73)	6.04	0.109
*C. macrostachyus* (seeds)	3.16 ± 0.13	16.13 (<.001)	0.48 (0.41–0.53)	2.50 (2.31–2.77)	5.94	0.101
*E. kebericho*	3.02 ± 0.08	36.27 (<.001)	0.96 (0.94–1.02)	4.10 (3.89–4.36)	7.46	0.059
*E. globulus*	3.47 ± 0.11	32.15 (<.001)	0.83 (0.79–0.87)	3.87 (3.64–4.14)	3.67	0.299
*J. procera*	3.53 ± 0.10	35.46 (<.001)	0. 97 (0.92–1.06)	4.83 (4.55–5.16)	7.25	0.064

Abbreviations: B, regression slope; SE, standard error; *Z*, Wald Z-statistic; *p*, p-value; χ², Chi-square goodness-of-fit statistic; df, degrees of freedom; ED_50_, effective dosage required to repel 50% of the mosquito population; ED_99_, effective dosage required to repel 99% of the mosquito population; 95% CI, 95% confidence interval.

### Repellency duration of the essential oils

**Fig 1:**
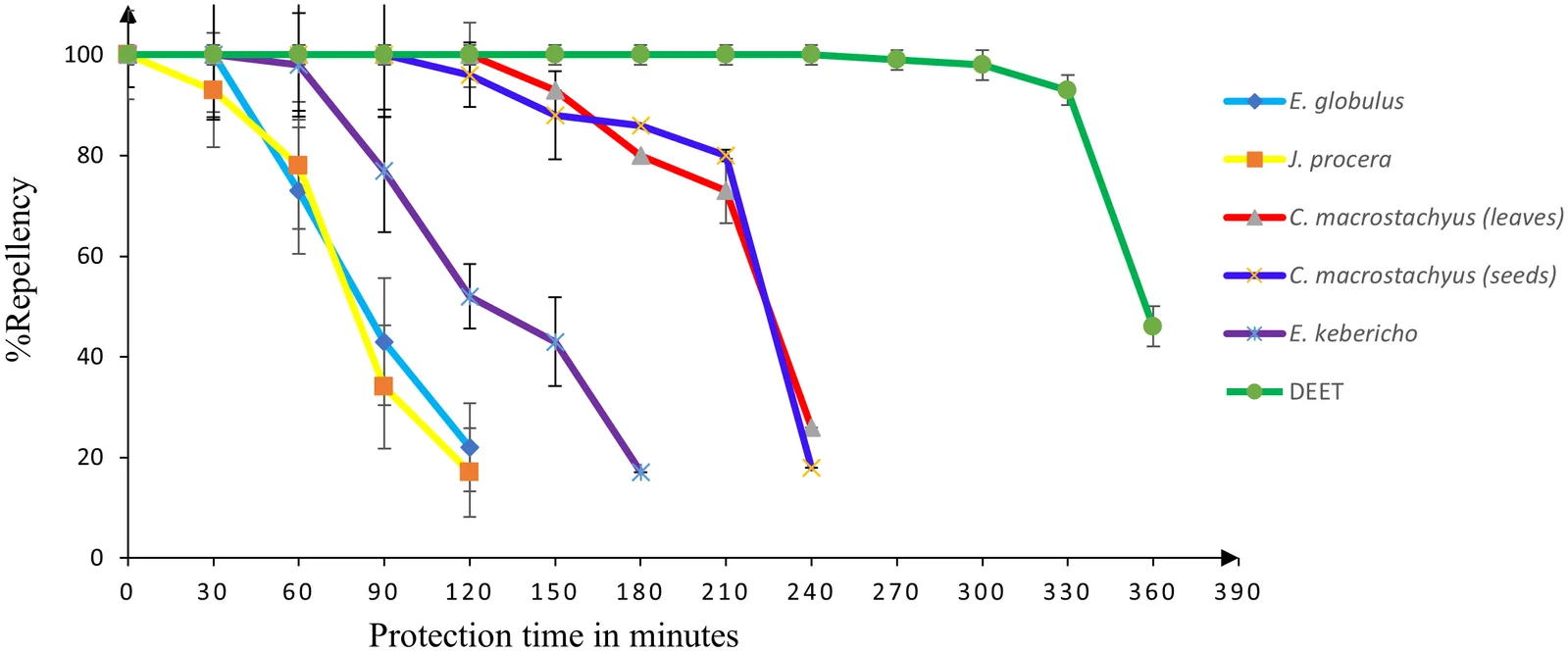
Percentage repellency of five essential oils and DEET at a 20% concentration against laboratory-reared *Anopheles arabiensis,* 2025.

Essential oils isolated from *C. macrostachyus* leaves and seeds provided protection for up to 180 minutes, with a percentage repellency ranging from 87% to 100% at concentration of 20%. After 210 minutes, the repellency decreased to a range of 47% to 53%, and eventually reached 0% after 270 minutes. The essential oil of *J. procera* provided the shortest protection time, exhibiting 93% to 100% repellency at 30 minutes post-application and decreasing to 70% to 80% by 60 minutes. DEET, a standard commercial repellent, showed significantly greater repellency, maintaining 93% to 100% efficacy for up to 330 minutes of the test duration against *An. arabiensis* (Fig 1).

**Fig 2:**
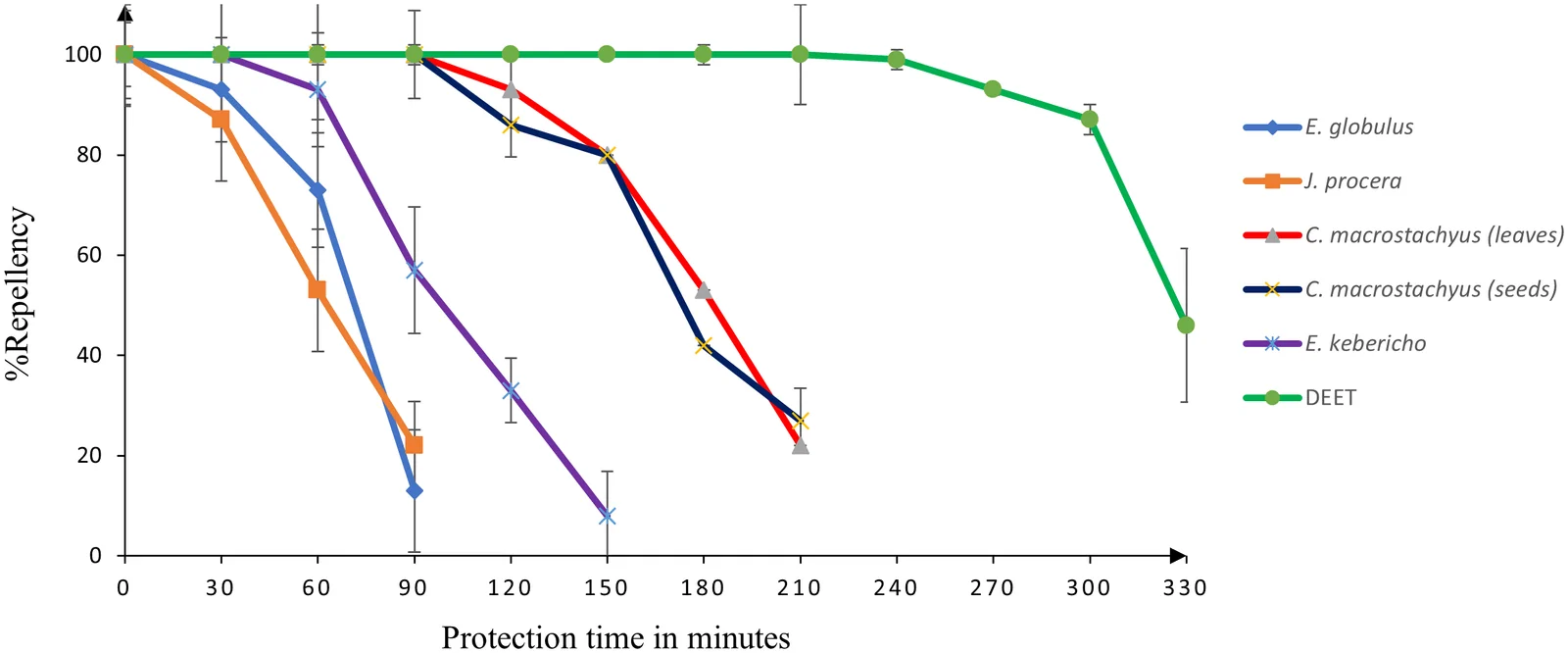
Percentage repellency of five essential oils at a 10% concentration and DEET against laboratory-reared *Anopheles arabiensis,* 2025.

All five essential oils demonstrated repellent activity against *An. arabiensis*, providing 80% to 100% protection for 30 minutes at a 10% concentration (Fig 2). Both essential oils from *C. macrostachyus* seeds and leaves provided protection for up to 150 minutes with a repellency range of 86% to 93%. Similarly, *E. kebericho* maintained 86% to 93% repellency for 60 minutes with percentage repellency, whereas *J. procera* oil showed the shortest protection time, decreased to 53% or lower by 60 minutes post-application. DEET, the standard commercial repellent, provided significantly longer repellency, maintaining 84% to 100% for up to 330 minutes post-application.

A Kruskal-Wallis H test showed that the protection time differed significantly (*p* < 0.001) among the five essential oils and DEET at both 10% and 20% concentrations. A pairwise Dunn’s post hoc test demonstrated that the essential oils from *C. macrostachyus* leaves, *C. macrostachyus* seeds and *E. kebericho* roots provided a significantly longer protection duration than *E. globulus* and *J. procera* oils (*p* < 0.05) (Table 7).

**Table 7:** Median protection time of five essential oils and DEET against laboratory-reared *Anopheles arabiensis*, 2025.

Essential oil formulations	10% concentration		20% concentration	
	Median protection time (minutes)	Range (min-max)	Median protection time (minutes)	Range (min-max)
DEET (Positive Control)	390^a^	270–390	362.00^a^	330–390
*C. macrostachyus* (leaves)	152^b^	150–153	211^b^	153–241
*C. macrostachyus* (seeds)	141^b^	93–153	214^b^	151–270
*E. kebericho*	92^b^	62–151	151^b^	91–153
*E. globulus*	62^c^	31–91	91^c^	33–152
*J. procera*	33 ^c^	31–63	61^c^	32–91

Data represent the median values of *n* = 5 subjects, evaluated in 3 independent replicates. The superscript letters (^a, b, c^) attached to values within a column indicate statistically significant differences (Dunn’s post-hoc test for multiple comparisons). Abbreviations: min–max, minimum to maximum value range.

### Duration of repellent effects from blended essential oils

**Fig 3:**
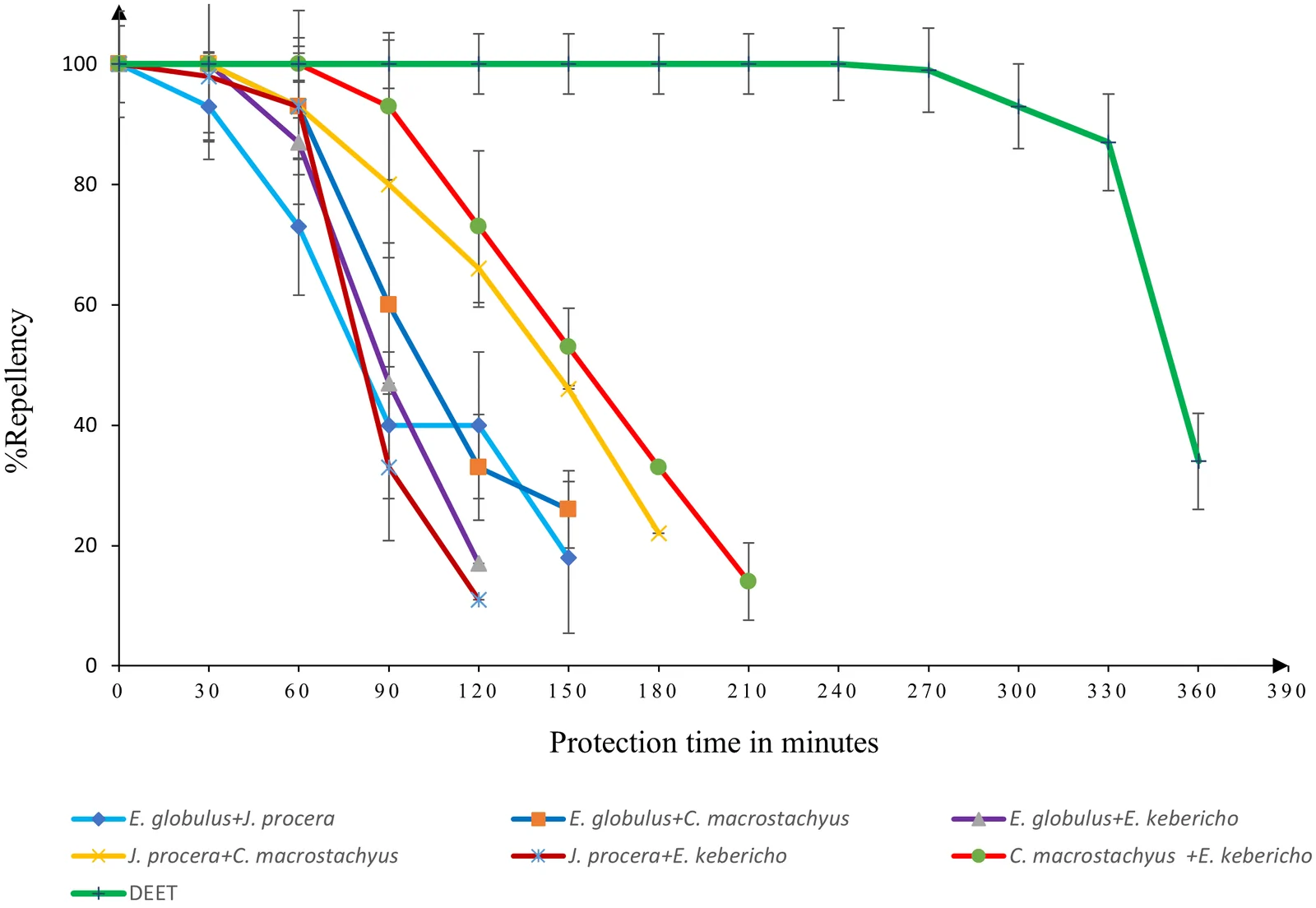
Percentage repellency of six blended essential oils and DEET at a 20% concentration against laboratory-reared *Anopheles arabiensis,* 2025.

All essential oil combinations provided significant protection soon after the application. However, the *C. macrostachyus*+*E. kebericho* and *J. procera*+*C. macrostachyus* blends demonstrated the longest protection times, maintaining greater than 80% protection against *An. arabiensis* for 90 minutes (Fig 3).

A Kruskal-Wallis H test showed that the median protection time differed significantly (*p* < 0.001) between binary oil combinations and DEET. The pairwise Dunn’s post hoc test demonstrated that two of the six blended oils, *C. macrostachyus* seeds + *E. kebericho* and *J. procera* + *C. macrostachyus* seeds, provided significantly longer median protection time than the other formulations (Table 8).

**Table 8:** Median protection time of six blended essential oils and DEET at a 20% concentration against laboratory-reared *Anopheles arabiensis*, 2025.

Blended essential oil formulations	Median protection time (minutes)	Range (min-max)
DEET	390^a^	330–390
*C. macrostachyus* (seeds) + *E. kebericho*	152^b^	90–211
*J. procera* + *C. macrostachyus* (seeds)	150^b^	61–153
*E. globulus* + *C. macrostachyus* (seeds)	93^c^	33–212
*E. globulus* + *E. kebericho*	93^c^	61–150
*J. procera* + *E. kebericho*	91^c^	63–93
*E. globulus* + *J. procera*	91^c^	32–150

Data represent the median values of *n* = 5 subjects, evaluated in 3 independent replicates. The superscript letters (^a, b, c^) attached to values within a column indicate statistically significant differences (*p* < 0.05, Dunn’s post-hoc test for multiple comparisons). Abbreviations: min–max, minimum to maximum value range.

A Kruskal-Wallis H test revealed significant differences in protection time when comparing single oils to their binary blends (*p* < 0.001). A pairwise comparisons using Dunn’s post hoc test showed that blending with *C. macrostachyus* seeds or *E. kebericho* significantly improved repellent efficacy compared to *J. procera* alone (*p* < 0.001) (Table 9).

**Table 9:** Comparisons of median protection time between the single and blended essential oils at a 20% concentration against laboratory-reared *Anopheles arabiensis*, 2025.

Single and blended oil formulations	Median protection time (minutes)	Range (min-max)
*C. macrostachyus* (seeds)	214^a^	151–270
*E. kebericho*	151^a^	91–153
*E. globulus*	91^ab^	33–152
*J. procera*	61^b^	32–91
*C. macrostachyus* (seeds) + *E. kebericho*	152^a^	90–211
*J. procera* + *C. macrostachyus* (seeds)	150^a^	61–153
*E. globulus* + *C. macrostachyus* (seeds)	93^a^	33–212
*E. globulus* + *E. kebericho*	93^a^	61–150
*J. procera* + *E. kebericho*	91^a^	63–93
*E. globulus* + *J. procera*	91^ab^	32–150

Data represent the median values of *n* = 5 subjects, evaluated in 3 independent replicates. The superscript letters (^a, b^) attached to values within a column indicate statistically significant differences. Values sharing a common letter are not significantly different (Dunn’s post-hoc test for multiple comparisons). Abbreviations: min–max, minimum to maximum value range.

## Discussion

The present study evaluated the essential oils extracted from parts of selected plants such as *C. macrostachyus*, *E. kebericho*, *E. globulus,* and *J. procera* in the Ghibe valley for their repellency against *An. arabiensis* under laboratory condition. In this study, a high yield of essential oil was obtained from the leaves of *E. globulus.* Essential oil yields of fresh *E. globulus* in Ethiopia and elsewhere range from over 0.2% to 5% with varying degrees depending on location, season, and age, with younger leaves generally yielding more oil in line with the result of the current study [51]. Among the four evaluated plants, the lowest oil yield was obtained from leaves of *C. macrostachyus,* which is consistent with the low essential oil yields from the berries of this species using hydrodistillation noted in previous studies [52]*.* This variation in plant parts explains the minor differences in overall yield, as these structures possess distinct metabolic characteristics.

A total of 131 chemical constituents were identified from the four essential oils assessed for their chemical composition in this study. Of these, the most abundant compounds have previously been reported for repellent properties such as Eucalyptol, 3-Carene and α-Terpinyl acetate, 13-epi-manoyl oxide, γ -Muurolene, benzoic acid-phenylmethyl ester, and Dehydrocostus lactone were identified [53,54]. While these individual compounds are reported in the literature to repel mosquitoes, the specific interactions among them in the current study remain to be fully investigated [53,55,56]. Other less abundant constituents identified from the essential oils, such as linalool and *α*-pinene have been previously documented to possess repellent properties [57]. Similarly, *β*-phellandrene was identified from *J. procera* oil, which has been reported as mosquito repellent [58].

The current investigation found that essential oils of the selected four plants, namely *C. macrostachyus*, *E. kebericho*, *E. globulus* and *J. procera,* have substantial repellent properties with varying levels of effects against *An. arabiensis* bites. As a result, the findings of this study supported the findings of the earlier ethnobotanical study on the usage of these plants for the traditional control of mosquitoes and other arthropods in the Ghibe Valley, southwest Ethiopia [32]. The present study revealed that essential oil from the leaf and seed parts of *C. macrostachyus* showed promising repellent activity. A comparison of the five essential oils’ ED_50_ values (dosage giving a 50% repellent effect) against *An. arabiensis* revealed that the repellent properties varied with dose. Among the five essential oils evaluated, those extracted from the leaves and seeds of *C. macrostachyus* demonstrated statistically identical repellent activities. The ED_50_ of both oils of *C. macrostachyus* was lower compared to the five essential oils. These results demonstrated that oils from both the leaf and seed parts of *C. macrostachyus* had the strongest repelling efficacy. This may be attributed to the variations in the type and quantity of active compounds present in the oil, which could influence repelling properties [59]. The scientific literature on the repellent efficacy of essential oils against *An. arabiensis* is often inconsistent [22]. These deviations often correlate with the concentration of the essential oil tested and the physicochemical properties of the carrier solvent [60]. Furthermore, the chemical profiles and biological efficacy of essential oils are variable may be because of the locality of plants, extraction methods, and the bioassay protocols. Additionally, variations in experimental parameters, such as dosages, formulations, mosquito species, and human volunteers may account for the inconsistencies [61]. For example, in some prior laboratory and field trials, protection duration was defined as the time elapsed until the first or second mosquito landing, whereas other studies utilized the onset of actual biting activity as the terminal endpoint [22,62].

Among the five tested essential oils, leaf and seed oils of *C. macrostachyus* protected for more than 150 minutes at a 10% concentration and increased to 180 minutes at a 20% concentration. The increased protection period of the oil at greater concentrations could be attributed to an increase in the concentration of the active ingredient contained in the oils. This demonstrates that increasing the concentration of essential oils can increase repellency, which is supported by the findings of several previous studies [35,63]. However, a major limitation of using concentrated essential oils is the potential risk of skin irritation or allergic reaction. Therefore, future research should include dermal toxicity assessments to balance the repellent efficacy with human skin safety. The duration of protection exhibited by the essential oil of *C. macrostachyus* in the present study is longer than *O. suave, O. americanum, O. kilimandscharicum,* and *Lantana camara* oils against *An. gambiae,* which have shown less than an hour of complete protection in laboratory studies in Kenya [64]. The protection duration of *C. macrostachyus* oils observed in the current laboratory screening was also greater than those of known botanical repellents. Specifically, exceeds the 60-minute rapid volatile decay typically observed in monoterpene-rich *Mentha* × *piperita* and citral-dominated matrices like *C. citratus* oils [45]. Furthermore, this sustained repellency are comparable to the 120–240-minute protection benchmark typically exhibited by heavier phenolic matrices, such as undiluted *S. aromaticum* oil [30]. However, literature indicates that the practical application of essential oils as long-lasting insect repellents is often limited by their high volatility and rapid oxidation rates [65]. The Environmental Protection Agency (EPA) of the United States has established that 120 minutes should be the standard minimum time required for a repelling effect of greater than 80% protection in order to qualify for registration [66,67]. In line with this, the essential oils extracted from both the leaves and seeds of *C. macrostachyus* demonstrated promising protection against laboratory-reared *An. arabiensis.* The evaluation of essential oil of *C. macrostachyus* for its repellency against *An. arabiensis* is the first in Ethiopia in spite of its wide use as herbal medicine by the indigenous people of tropical Africa [68]. For example, a recent laboratory study in the country reported that the methanol crude extract and ethyl acetate fractions of *C. macrostachyus* leaves showed a promising larvicidal and adulticidal activity against *An. gambiae* [69]. However, further studies are required with larger sample sizes, a comprehensive dermal toxicity assessments and field validation because laboratory bioassays alone may not fully predict field performance due to environmental factors such as wind, temperature fluctuations, and natural mosquito behavioral variability.

Essential oil of *E. kebericho* protected the skin for more than 90 minutes at 20% concentrations. These findings support the traditional usage of *E. kebericho* as a mosquito repellent in the Ghibe valley, Ethiopia [32]. Although a previous study confirmed that *E. kebericho* oil exhibits strong repellency against *An. arabiensis* at low concentrations, its specific protection time was not reported. However, it provided a longer protection time than the oils of *O. suave, O. americanum, O. kilimandscharicum,* and *Lantana camara* against *An. gambiae* [64].

*E. globulus* and *J. procera* protected the skin for less than an hour in both 10% and 20% concentrations, with *E. globulus* protected for more than an hour at 20% concentration. Comparable result was reported by Wano [70], who evaluated *E. globulus* essential oil against *An. arabiensis* using 10% and 20% concentrations in laboratory arm-in-cage bioassays with 30 female mosquitoes on human volunteers. The complete protection time of *E. globulus* and *J. procera* was comparable *to O. suave, O. americanum, O. kilimandscharicum* and *Lantana camara An. gambiae* [64]. A previous study conducted in Ethiopia demonstrated that the methanol extract of *J. procera* provided significant protection against *An. arabiensis* [35]. Furthermore, it has been reported that the repellency of methanol extract of the *J. procera* increases in a concentration-dependent manner [35].

When comparing the repellent effects of the essential oil blends, the 1:1 binary combination of *C. macrostachyus* with *E. kebericho*, *J. procera* with *C. macrostachyus* (seeds) demonstrated a greater effectiveness than the remaining oil combinations. The importance of blending essential oils is modifying the interactions of their active chemical constituents [71]. The biological consequences of these blends may impact repellent efficacy of either in synergistic interactions that increase protection time or antagonistic that reduce protection time. The result of the current study showed that blending with *C. macrostachyus* (seeds) or *E. kebericho* significantly improves repellent efficacy or possesses a strong synergistic effect compared to *J. procera* alone. This strong synergistic effect might be due to the heavy sesquiterpene lactones and esters found in *E. kebericho* and *C. macrostachyus* act as natural fixatives [72]. But other combinations preserve the repellent efficacy of the stronger component. These findings suggest that most of blended essential oils exhibited comparable repellency to single essential oils with minor differences. This may be due to competitive binding at identical mosquito olfactory receptors or independent evaporation, which prevent any increase of protection time relative to individual oils [45].

The shorter protection duration of the essential oils compared to DEET in the current study may suggest that there is faster loss of repellent activity due to faster volatilization of compounds of the essential oils [73]. To maximize the effectiveness of essential oils as repellent against mosquito bites, particular formulations might require. Adding 5% vanillin to *E. globulus* oil increased its protection period against *Ae. albopictus* from 3–5 hours under laboratory conditions in China [74]. Citronella oil has long been used in commercial repellent formulations and is popular in India, however it is generally rated as less effective than repellents using synthetic active ingredients [75]. Despite the fact that all of the essential oils employed in this study are less effective than DEET, they are thought to be safer and can be used frequently. These essential oils can provide a safe, affordable, and practical alternative for mosquito control due to the local availability and easy application of the source plants. They can be produced sustainably at low cost, making them potential alternatives for local community level protection.

### Limitations of the study

A notable limitation of this study is that individual bioassays were not conducted using the isolated major chemical constituents of the essential oils to identify potent repellent chemicals against mosquitoes. This study did not test complex mixtures containing three or more oils, limiting our understanding of the potential synergistic or antagonistic interactions that may occur within multi-component oil blends. The evaluation was limited to a single application method, failing to compare a surface painting with spatial sprays, human-bait arm-in-cage tests and heated diffusion methods. The residual efficacy and applicability of these essential oils were also not optimized by advanced formulations, such as macro- and nano-encapsulation techniques, which are required for reducing evaporation rates and improving protection times. Furthermore, an economic feasibility analysis was not performed to evaluate the cost-effectiveness of large-scale essential oil extraction and practical formulation deployment. A dermal toxicity assessment was not performed, which is required for establishing safety margins. The use of only five participants also limits broad statistical generalization of the current screening results, despite using a repeated measures design.

## Conclusions

In total, 131 chemical constituents were identified across the four analyzed essential oils. In this laboratory screenings, all five evaluated essential oils demonstrated strong repellency against laboratory-reared *An. arabiensis*, though their effectiveness varied and provided shorter protection time than DEET. Specifically, *C. macrostachyus* leaf and seed oils provided the longest protection time against laboratory-reared *An. arabiensis*, lasting 180 and 150 minutes at 20% and 10% concentrations, respectively. Additionally, blending *J. procera* oil with *C. macrostachyus* or *E. kebericho* produced a significant synergistic repellency (*p* < 0.001). Overall, the results underscore that *C. macrostachyus* leaf and seed oils have the potential to meet the regulatory performance standards for natural insect repellents. However, future research should include larger sample sizes, dermal toxicity assessments, and field validation to fully support the commercialization of these essential oils.

## Supporting information

S1 File GC-MS Chromatogram of essential oil of *Eucalyptus globulus.* (PDF)

S2 File GC-MS Chromatogram of essential oil of *Juniperus procera.* (PDF)

S3 File GC-MS Chromatogram of essential oil of *Croton macrostachyus.* (PDF)

S4 File GC-MS Chromatogram of essential oil of *Echinops kebericho.* (PDF)

S5 File Minimal datasets for dose response study. (XLSX)

S6 File Minimal datasets for complete protection time study of individual essential oils at 10% concentration. (XLSX)

S7 File Minimal datasets for complete protection time study individual essential oils and their blends at 20% concentration. (XLSX)

## Abbreviations

AAU: Addis Ababa University
ALIHR: Aklilu Lemma Institute of Health Research
CDT-Africa: Center for Innovative Drug Development and Therapeutic Trials for Africa
CPT: Complete Protection Time
DEET: N-diethyl-metatoluamide
ED: Effective Dosage
GC-MS: Gas Chromatography-Mass Spectrometry
SPSS: Statistical Package for the Social Sciences
RI: Retention Index
WHO: World Health Organization
