# Supplementary material for: Repellency and chemical composition of essential oils from four medicinal plants against Anopheles arabiensis (Diptera: Culicidae) under laboratory conditions
Source: PLoS One. 2026 Sep 15;21(9):e0358040. doi: 10.1371/journal.pone.0358040 (PMC13577348; doi:10.1371/journal.pone.0358040)

**Sample Name**		**Position**	1	**Instrument Name**	GCMS	**User Name**	
**Inj Vol**	1	**InjPosition**		**SampleType**		**IRM Calibration Status**	Not Applicable
**Data Filename**	Z-03.D	**ACQ Method**	EO ZED 1612 2025.M	**Comment**		**Acquired Time**	27-Dec-25 2:14:28 AM (

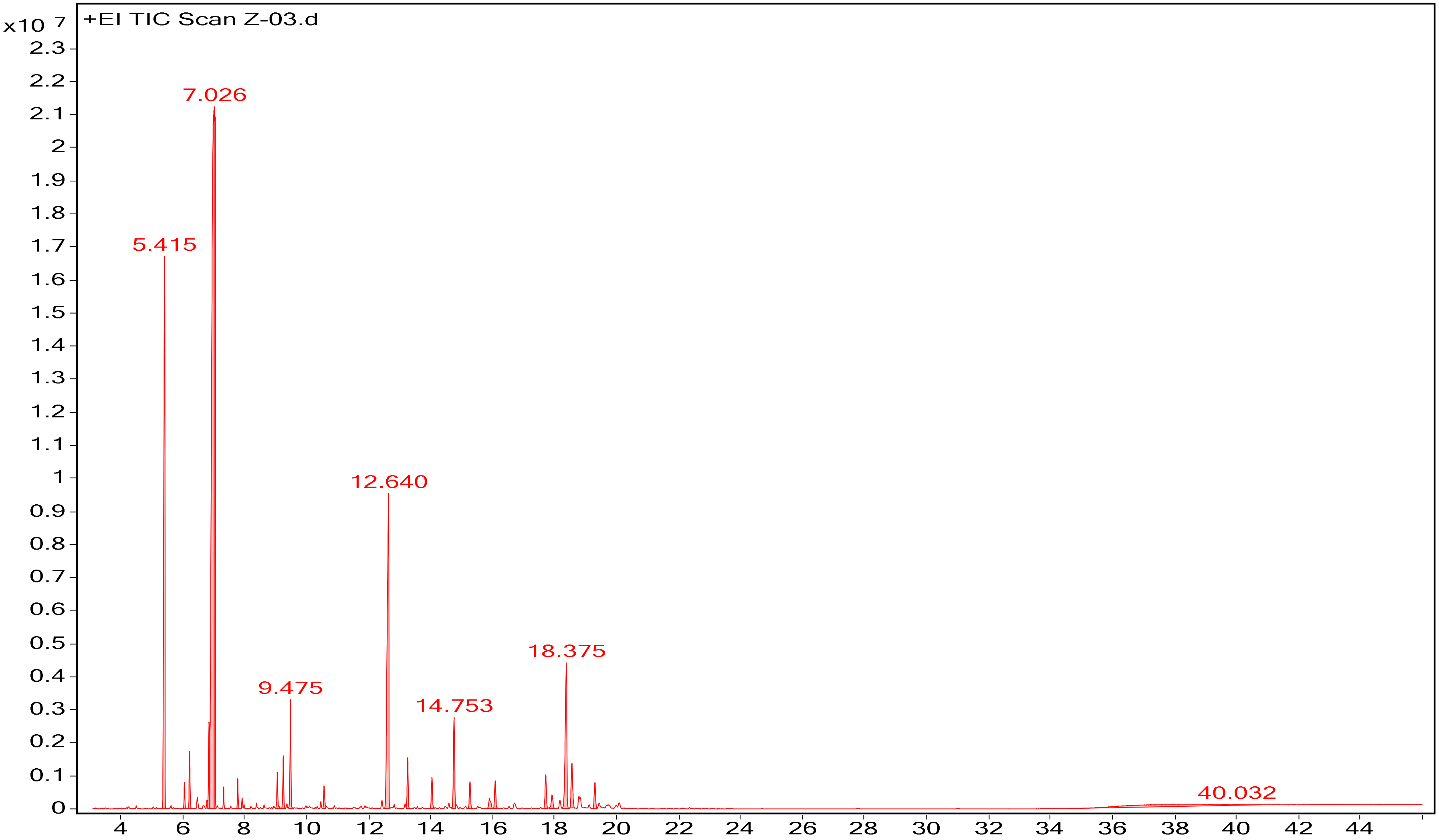

Supplement: Supplementary file 1 [file pone.0358040.s001.pdf]
