# Supplementary material for: Repellency and chemical composition of essential oils from four medicinal plants against Anopheles arabiensis (Diptera: Culicidae) under laboratory conditions
Source: PLoS One. 2026 Sep 15;21(9):e0358040. doi: 10.1371/journal.pone.0358040 (PMC13577348; doi:10.1371/journal.pone.0358040)
Supplement: Supplementary file 5 [file pone.0358040.s010.pdf]

PLOS One

Repellency of selected essential oils against Anopheles arabiensis (Diptera: Culicidae) under laboratory condition

--Manuscript Draft--

**Manuscript Number:**	PONE-D-26-14817R1
**Article Type:**	Research Article
**Full Title:**	Repellency of selected essential oils against Anopheles arabiensis (Diptera: Culicidae) under laboratory condition
**Short Title:**	Repellency of selected essential oils against Anopheles arabiensis (Diptera: Culicidae)
**Corresponding Author:**	Zeyede Teshome, M.SC.<br>Addis Ababa University<br>Addis Ababa, Ethiopia, ETHIOPIA
**Keywords:**	Essential oils, Ethiopia, Malaria, Medicinal plants, Mosquitoes, Repellency
**Abstract:**	The use of repellent provides human protection from mosquitoes and, hence, mosquito-borne diseases in light of the widespread shift in biting patterns. Plant-based repellents have traditionally been used to protect people from mosquitos. The current study aimed to assess the repellent effects of five essential oils against laboratory-reared Anopheles arabiensis bites, the major malaria vector in Ethiopia. Ethnobotanically investigated medicinal plants from the Ghibe Valley, southwest Ethiopia, namely Croton macrostachyus, Echinopskebericho, Eucalyptus globulus and Juniperus procera were used for extraction of essential oils. Essential oils were extracted from the fresh plant parts using hydro-distillation methods. The chemical compositions of the essential oils were analyzed by Gas Chromatography-Mass Spectrometry (GC-MS). Laboratory arm-in-cage bioassays with human volunteers were conducted to estimate the effective dosage and evaluate the complete protection time at fixed concentrations of 10% and 20%. The data was analyzed by probit plane regression for dosage estimation, Kaplan–Meier and Kruskal-Wallis H tests for protection time, and Spearman's rank correlation coefficient (ρ) to correlate chemical compounds with repellent efficacy. There were 162 chemical compounds identified, including benzoic acid-phenylmethyl ester (ρ = 0.775) and -gamma-Muurolene (ρ = 0.316) which, were most abundant compounds and showed a positive correlation trend with repellency and are known to have insect repellent properties. The findings revealed that C. macrostachyus leaves and seed oils have a significantly longer protection time (P < 0.001), achieving 180 minutes with a mean protection of 85-100%. Blending J. procera oil with C. macrostachyus (seeds) or E. kebericho resulted in a significant synergistic repellent effect (P < 0.001). Other oil combinations maintained the repellent efficacy of the stronger component. The protection duration of C. macrostachyus leaf and seed oils meets the Environmental Protection Agency's registration criteria. However, further study is required under field conditions as well as a skin toxicity assessment.
**Order of Authors:**	Zeyede Teshome, PhD candidate
	Abebe Animut, PhD
	Belete Adefris Legesse, PhD
	Mirutse Giday, PhD
	Esayas Aklilu, PhD
**Response to Reviewers:**	Please see attached response to reviewers file
**Additional Information:**	
**Question**	**Response**
**Financial Disclosure**<br><br>Enter a financial disclosure statement that	Yes

Powered by Editorial Manager® and ProduXion Manager® from Aries Systems Corporation

describes the sources of funding for the work included in this submission. Review the submission guidelines for detailed requirements. View published research articles from *PLOS ONE* for specific examples.<br><br>This statement is required for submission and **will appear in the published article** if the submission is accepted. Please make sure it is accurate.<br><br>**Funded studies**<br>Enter a statement with the following details:<br>• Initials of the authors who received each award<br>• Grant numbers awarded to each author<br>• The full name of each funder<br>• URL of each funder website<br>• Did the sponsors or funders play any role in the study design, data collection and analysis, decision to publish, or preparation of the manuscript?<br><br>Did you receive funding for this work?	
Please add funding details.<br>as follow-up to "**Financial Disclosure**<br><br>Enter a financial disclosure statement that describes the sources of funding for the work included in this submission. Review the submission guidelines for detailed requirements. View published research articles from *PLOS ONE* for specific examples.<br><br>This statement is required for submission and **will appear in the published article** if the submission is accepted. Please make sure it is accurate.	This study was financed by the Office of the Vice President for Research and Innovation, Addis Ababa University (grant number RD/PY-524/2023).

**Funded studies**<br>Enter a statement with the following details:<br>• Initials of the authors who received each award<br>• Grant numbers awarded to each author<br>• The full name of each funder<br>• URL of each funder website<br>• Did the sponsors or funders play any role in the study design, data collection and analysis, decision to publish, or preparation of the manuscript?<br><br>Did you receive funding for this work?"	
Please select the country of your main research funder (please select carefully as in some cases this is used in fee calculation).<br>as follow-up to "**Financial Disclosure**<br><br>Enter a financial disclosure statement that describes the sources of funding for the work included in this submission. Review the submission guidelines for detailed requirements. View published research articles from *PLOS ONE* for specific examples.<br><br>This statement is required for submission and **will appear in the published article** if the submission is accepted. Please make sure it is accurate.<br><br>**Funded studies**<br>Enter a statement with the following details:<br>• Initials of the authors who received each award<br>• Grant numbers awarded to each author<br>• The full name of each funder<br>• URL of each funder website<br>• Did the sponsors or funders play any role in the study design, data collection and analysis, decision to publish, or preparation of the manuscript?<br><br>Did you receive funding for this work?"	ETHIOPIA - ET
**Competing Interests**	The authors have declared that no competing interests exist.

Powered by Editorial Manager® and ProduXion Manager® from Aries Systems Corporation

Use the instructions below to enter a competing interest statement for this submission. On behalf of all authors, disclose any competing interests that could be perceived to bias this work—acknowledging all financial support and any other relevant financial or non-financial competing interests.<br><br>This statement is required for submission and **will appear in the published article** if the submission is accepted. Please make sure it is accurate and that any funding sources listed in your Funding Information later in the submission form are also declared in your Financial Disclosure statement.<br><br>View published research articles from *PLOS ONE* for specific examples.<br><br>**NO authors have competing interests**<br><br>Enter: *The authors have declared that no competing interests exist.*<br><br>**Authors with competing interests**<br><br>Enter competing interest details beginning with this statement:<br><br>*I have read the journal's policy and the authors of this manuscript have the following competing interests: [insert competing interests here]*<br><br>* typeset	
**Data Availability**<br><br>Authors are required to make all data underlying the findings described fully available, without restriction, and from the time of publication. PLOS allows rare exceptions to address legal and ethical concerns. See the PLOS Data Policy and FAQ for detailed information.	Yes - all data are fully available without restriction

A Data Availability Statement describing where the data can be found is required at submission. Your answers to this question constitute the Data Availability Statement and **will be published in the article**, if accepted.<br><br>**Important:** Stating 'data available on request from the author' is not sufficient. If your data are only available upon request, select 'No' for the first question and explain your exceptional situation in the text box.<br><br>Do the authors confirm that all data underlying the findings described in their manuscript are fully available without restriction?	
Additional data availability information:	
**Ethics Statement**<br><br>Enter an ethics statement for this submission. This statement is required if the study involved:<br><br>• Human participants<br>• Human specimens or tissue<br>• Vertebrate animals or cephalopods<br>• Vertebrate embryos or tissues<br>• Field research<br><br>Write "N/A" if the submission does not require an ethics statement.<br><br>General guidance is provided below. Consult the submission guidelines for detailed instructions. **Make sure that all information entered here is included in the Methods section of the manuscript.**	The Institutional Research Ethics Review Committee (ALIPB-IRERC) of Aklilu Lemma Institute of Health Research, Addis Ababa University, approved the study and issued an ethical clearance certificate (reference number ALIPB-IRERC/130/2016/24). Volunteers provided written informed consent on 13 June 2025 after explaining the procedures and benefits of the study prior to conducting every laboratory test of repellency using the in arm-in cage method from June 15 to November 30, 2025.

Powered by Editorial Manager® and ProduXion Manager® from Aries Systems Corporation

**Format for specific study types**<br><br>**Human Subject Research (involving human participants and/or tissue)**<br>• Give the name of the institutional review board or ethics committee that approved the study<br>• Include the approval number and/or a statement indicating approval of this research<br>• Indicate the form of consent obtained (written/oral) or the reason that consent was not obtained (e.g. the data were analyzed anonymously)<br><br>**Animal Research (involving vertebrate animals, embryos or tissues)**<br>• Provide the name of the Institutional Animal Care and Use Committee (IACUC) or other relevant ethics board that reviewed the study protocol, and indicate whether they approved this research or granted a formal waiver of ethical approval<br>• Include an approval number if one was obtained<br>• If the study involved *non-human primates*, add *additional details* about animal welfare and steps taken to ameliorate suffering<br>• If anesthesia, euthanasia, or any kind of animal sacrifice is part of the study, include briefly which substances and/or methods were applied<br><br>**Field Research**<br><br>Include the following details if this study involves the collection of plant, animal, or other materials from a natural setting:<br>• Field permit number<br>• Name of the institution or relevant body that granted permission	
**Data Availability**<br><br>Before publication, Authors are required to make fully available and without restriction all data underlying their findings. Sharing the data underlying your findings increases the visibility, credibility, and impact of your work, while supporting transparency and future scientific discovery. Please see our PLOS Data	All relevant data are within the manuscript and its Supporting Information files.

Policy page for detailed information on this policy.<br><br>A **Data Availability Statement**, detailing where the data can be accessed, is required at first submission. Insert your Data Availability Statement in the box below.<br><br>Please see the data reporting section of our submission guidelines for instructions on what you need to include in your Data Availability Statement. Examples of acceptable Data Availability Statements are included here for reference:<br>1. The minimal data set [and accompanying code, where generated] is available at [repository name] via [DOI hyperlink]<br>2. No data was generated by this study. The following existing data sources were used: [dataset name] from [dataset location] available via [web address or DOI hyperlink].<br>3. For Study Protocols: No datasets were generated or analysed during the current study. All relevant data from this study will be made available upon study completion.<br><br>*PLOS allows rare exemptions to address legal and ethical concerns. If you have legal or ethical restrictions, please use the box below to detail these in full sentences for the Journal team to consider.*<br><br>This statement will be typeset if the manuscript is accepted for publication.	

Powered by Editorial Manager® and ProduXion Manager® from Aries Systems Corporation

Manuscript

Click here to access/download;Manuscript;Manuscript.docx

Repellency of selected essential oils against *Anopheles arabiensis* (Diptera: Culicidae) under laboratory condition

Zeyede Teshome[1,2*], Abebe Animut[2], Belete Adefris Legesse[3], Mirutse Giday[2], Esayas Aklilu[2]

[1]Department of Biology, Woldia University, P.O. box 50, Woldia, Ethiopia.

[2]Aklilu Lemma Institute of Health Research, Addis Ababa University, P O Box 1176, Addis Ababa, Ethiopia.

[3]Center for Innovative Drug Development and Therapeutic Trials for Africa (CDT-Africa), Addis Ababa University, P O Box 1176, Addis Ababa, Ethiopia.

*Correspondence:

Zeyede Teshome

zeyedetw21@gmail.com (Z.T.)

Abstract

The use of repellent provides human protection from mosquitoes and, hence, mosquito-borne diseases in light of the widespread shift in biting patterns. Plant-based repellents have traditionally been used to protect people from mosquitos. The current study aimed to assess the repellent effects of five essential oils against laboratory-reared *Anopheles arabiensis* bites, the major malaria vector in Ethiopia. Ethnobotanically investigated medicinal plants from the Ghibe Valley, southwest Ethiopia, namely *Croton macrostachyus*, *Echinops kebericho*, *Eucalyptus globulus* and *Juniperus procera* were used for extraction of essential oils. Essential oils were extracted from the fresh plant parts using hydro-distillation methods. The chemical compositions of the essential oils were analyzed by Gas Chromatography-Mass Spectrometry (GC-MS). Laboratory arm-in-cage

bioassays with human volunteers were conducted to estimate the effective dosage and evaluate the complete protection time at fixed concentrations of 10% and 20%. The data was analyzed by probit plane regression for dosage estimation, Kaplan–Meier and Kruskal-Wallis H tests for protection time, and Spearman's rank correlation coefficient (ρ) to correlate chemical compounds with repellent efficacy. There were 162 chemical compounds identified, including benzoic acid-phenylmethyl ester ($\rho = 0.775$) and -gamma-Muurolene ($\rho = 0.316$) which, were most abundant compounds and showed a positive correlation trend with repellency and are known to have insect repellent properties. The findings revealed that *C. macrostachyus* leaves and seed oils have a significantly longer protection time ($P < 0.001$), achieving 180 minutes with a mean protection of 85-100%. Blending *J. procera* oil with *C. macrostachyus* (seeds) or *E. kebericho* resulted in a significant synergistic repellent effect ($P < 0.001$). Other oil combinations maintained the repellent efficacy of the stronger component. The protection duration of *C. macrostachyus* leaf and seed oils meets the Environmental Protection Agency's registration criteria. However, further study is required under field conditions as well as a skin toxicity assessment.

Keywords: Essential oils, Ethiopia, Malaria, Medicinal plants, Mosquitoes, Repellency

Introduction

Malaria causes significant public health challenge despite decades of control efforts. It infected over 280 million people and claimed 600,000 lives in 2024 of which over 90% of the cases and deaths were from Africa [1, 2]. About 75% of the population lives in high-endemic areas, 18% in epidemic-prone and only 7% in low- or malaria-free [3, 4]. The disease is the top cause of morbidity and mortality in Ethiopia [5-7].

Over 70 *Anopheles* species transmit malaria of which *Anopheles gambiae* s.s. and *An. arabiensis* are the most effective vectors, while *An. funestus*, *An. bwamnbe*, *An. merus*, and *An.*

melas are involved in localized transmission in sub-Saharan Africa [8, 9]. The predominant and most widespread vector in Ethiopia is *An. arabiensis* while *An. nili*, *An. pharoensis*, and *An. funestus* serve as secondary roles in limited settings [10, 11]. *An. stephensi*, reported recently, is also a potential vector [12, 13].

Vector control remained the primary strategy to combat malaria [14, 15]. The use of long-lasting insecticide-treated nets and indoor residual insecticide spraying has been effective to control malaria in indoor settings [14, 16, 17]. However, these strategies are being challenged by vectors resistance to insecticides [18, 19], shift in biting from late-night to early-night and indoor to outdoor [20, 21]. This entails the need for alternative control tools including mosquito repellents to suppress vectors regardless of their biting patterns [18, 22].

The use of repellents has been acknowledged in vector management [23, 24]. Repellents received substantial attention in recent years, largely because of increasing behavioral changes of mosquitoes and outdoor malaria transmission [22]. Commercial repellents made from synthetic chemicals like N-diethyl-metatoluamide (DEET), allethrin, N-diethyl mendelic acid amide, and dimethyl phthalate are commonly used for protecting against mosquito bites [23]. However, repeated application of synthetic repellents induces potential health concerns in humans and poses ecotoxicological risks [25-27]. Thus, safe, economical and environment friendly repellent, including from plants, remains a priority [25, 28].`

The oils, smokes, tars, etc. have been employed to repel mosquitoes in Ethiopia, including the Ghibe valley, Ethiopia [29, 30]. Plant based repellents received increased interest in recent years due to their concentrated bioactive phytochemicals that are both safe and biodegradable [25, 31]. Essential oils of plants are complex mixtures of aromatic and chemically pure compounds that convey the unique fragrance and properties of plants that vary greatly by genetics, climate, rainfall

and location [32]. Previous studies in Ethiopia demonstrated essential oils of different plants with repellent properties against malaria vectors [33-36]. There are ethnobotanically claimed medicinal plants used to control mosquitoes and other insects in the Ghibe valley, southwest Ethiopia, previously identified and documented by our ethnobotanical survey [30]. However, the repellency of essential oils derived from such traditionally/ethnobotanically claimed medicinal plants against *An. arabiensis,* the major malaria vector in Ethiopia remains scarce. The current study investigated the repellent properties of essential oils extracted from *Croton macrostachyus* Hochst. ex Delile, *Echinops kebericho* Mesfin*, Eucalyptus globulus* Labill. and *Juniperus procera* Hochst. ex Endl. against laboratory reared female *An. arabiensis* bite. Additionally, the chemical composition of the oils was assessed to better understand their efficacy and potential applications in mosquito repellency.

Materials and methods

Plant selection and collection

Plants selected on the basis of our previous ethnobotanical survey between March and October, 2024 in Deri Saja Zuria district, Enor, Misha, and Sekoru in the Ghibe Valley, southwest Ethiopia were evaluated [30]. The plants were *C. macrostachyus*, *E. kebericho*, *E. globulus,* and *J. procera* Plant parts were carefully collected in accordance with the local species protection and IUCN regulations, with less affected the wild populations in the areas between April and November 2025. Accordingly, fully developed leaves and seeds of *C. macrostachyus* along with leaves of *E. globulus*, were collected from their natural habitat in the Enor district. Leaves of *J. procera* were gathered from natural habitats in the Misha district, while roots of *E. kebericho* were harvested

from local farmers in the Deri Saja Zuria district, where they are cultivated and prepared for the local market.

Essential oil extraction from plant parts

Essential oils were extracted by hydro-distillation processes using a Clevenger apparatus at natural product laboratory of CDT Africa, Addis Ababa University, using two distillation flasks with a capacity of 600 grams. Fresh plant parts (1200 g each) were cut into small pieces and placed in a distillation flask (600 g per flask) with enough water (3000 mL per flask) added. The distillation chamber was heated at about 80-100℃ and allowed to boil for more than three hours. The distillate was collected in a separating funnel in which the aqueous portion was separated from the volatile oil. The water layer was slowly drawn off until only the oil layer would remain. The oil was collected in a 10ml glass bottle and stored at 4℃ until it used for the chemical analysis and repellency test against *Anopheles arabiensis* bite.

Gas chromatography/mass spectrometry (GC/MS) analysis

The essential oils of *E. globulus, J. procera, C. macrostachyus* and *E. kebericho* were analyzed using GC-MS at the laboratory of natural product chemistry, Adama Science and Technology University. GC-MS analysis was performed using a GC (7890B, Agilent Technologies, China) coupled with an MS (5977B Network, Agilent Technologies and USA). The GC had an HP-5MS column (non-polar column, Agilent Technologies), 30 m × 250 µm internal diameter and 0.25 µm film thickness. The carrier gas was helium flowing at a rate of 1 mL/minute. 1µL sample was injected, and the injector temperature was adjusted to 250°C and the injection mode was split mode with a split ratio of 50:1. The initial oven temperature was programmed from 50˚C, held for 1 minute. It was raised to 120˚C at 10˚C/minute and then ramped by 4˚C/minute to reach 220˚C, and

finally raised to 280 with the rate of 20˚C/minute at this temperature held for 10 minutes. Mass spectra were recorded in EI mode at 70 eV, scanning the 45-550 m/z range. Percentage of each compound was calculated by area of a specific compound divided by the sum area of all identified compounds and multiplied by 100 [37].

Ethics approval and consent to participate

The Institutional Research Ethics Review Committee (ALIPB-IRERC) of Aklilu Lemma Institute of Health Research, Addis Ababa University, approved the study and issued an ethical clearance certificate (reference number ALIPB-IRERC/130/2016/24). Written permission letters for field access to all plant collection sites were obtained from the Institute of Public Health in the Central Ethiopia Regional State, as well as from the health office of each study district. Volunteers provided written informed consent on 13 June 2025 after receiving explanations of the procedures and benefits of the study. This consent was obtained prior to conducting each laboratory test of repellency using the arm-in-cage method, which took place from June 15 to November 30, 2025.

Evaluating repellency of essential oils

The repellency of essential oils derived from various plant parts, namely *C. macrostachyus* (leaves and seeds), *E. kebericho* (roots), *E. globulus* (leaves) and *J. procera* (leaves) was evaluated against a laboratory colony of female *An. arabiensis*, which was maintained at the Aklilu Lemma Institute of Health Research, Addis Ababa University following the WHO guidelines [38]. Each essential oil was diluted in ethanol to prepare a range of serial dilutions at various concentrations. Thus, five different v/v concentrations, such as 10 µL oil in 1mL ethanol (1%), 25 µL oil per1 mL ethanol (2.5%), 50 mL oil per 1 mL ethanol (5%), 75 µL oil per 1mL ethanol (7.5%) and 100 µL oil per 1 mL ethanol (10%) were prepared for the dose response test. Additional concentrations, such as 100

µL oil per 1 mL ethanol (10%) and 200 µL oil per 1 mL of ethanol (20%), were prepared to evaluate the protection duration of each essential oil. Additionally, a total of six binary combinations of the essential oils were created, including *globulus* + *J. procera, E. globulus* + *C. macrostachyus* (seeds), *E. globulus* + *E. kebericho, J. procera* + *C. macrostachyus* (seeds), *J. procera* + *E. kebericho,* and *C. macrostachyus* (seeds) + *E. kebericho,* all mixed in a 1:1 v/v ratio. The required concentration of 200 µL of oil per 1 mL of ethanol (20% v/v) for each combination was then prepared to evaluate the effects of the blended oils.

The test mosquitos were reared, maintained and tested in a separate room under optimal conditions. Mosquitoes were maintained at 27 ± 2°C, 60 to 70% relative humidity, and had constant access to a 10% sugar solution. Nulliparous female mosquitoes were starved for 12 hours before the repellent test began. Standardized cages (30cm × 30cm × 30cm) were used for daytime testing in a dark room.

Five healthy adult human volunteers (>30 years old) with no history of allergic reaction to mosquito bites were selected to participate in the experiment. Participants were encouraged to avoid alcohol, tobacco, and any scented products for at least 12 hours before the experiment. The amount of each test oil applied on the arm was determined from the surface area of the arm (in cm^2) of skin is then calculated according to the formula (38). Area = ½ $(C_w + C_e) D_{we}$ where, C_w is the circumference of the wrist in cm, C_e is the elbow-cubital fossa circumference in cm, and D_{we} is the distance in between Ce and Cw. Accordingly, the calculated surface of the arm of the exposed volunteers were as follows (V1= 562.5 cm^2, V2= 516 cm^2, V3= 588 cm^2, V4= 572.5 cm^2, and V5= 546.8 cm^2).

Volunteers were required to clean their hands and arms thoroughly followed by drying. A surgical glove was then worn on the hand to prevent biting on the untreated hand. The test area of

the exposed volunteer (the inner part of the forearm between the wrist and elbow) was washed with unscented soap and ethanol. Each test consisted of two parts: the right arm was treated with the essential oils, while the left arm served as the control. Each volunteer was exposed to each essential oil once per day. During the time intervals between exposure periods, volunteers were instructed not to rub, touch, or wet the treated arms and were restricted to stay in controlled room to minimize the loss of the oils from the treated forearms.

Estimation of effective dose: In this bioassay, a range of doses (1-10%) of all essential oils was tested to determine the effective dosage (ED_{50} and ED_{99}). One hundred nulliparous female mosquitoes (5-7 days old) were placed in a cage and allowed to acclimatize for approximately 2 hours. Their biting readiness was then tested by exposing only an ethanol-treated forearm (control arm) for up to 30 seconds. When at least 10 mosquitoes landed on the control arm within 30 seconds, the test continued. The control forearm was removed, and the desired concentration of the oils, with an amount of 1.67 μL/cm², was applied uniformly on the treatment arms of the volunteers. The successive doses of oils were tested one after the other starting from the lowest concentration to the highest, until no landing was recorded. The repellent dose for each test was calculated as the sum of the doses applied, resulting in a cumulative dose for each test [38]. To prevent the effect of evaporation, the interval between applications was minimum and exposure of each dose lasting 30 seconds. Each test concentration was repeated 3 times per volunteer in different days using fresh mosquitoes. The number of mosquito landing was recorded and used in data analysis.

Duration of mosquito landing protection: In this bioassay, due to the highly volatile nature of the essential oils and vary significantly in their calculated ED_{99} values, they were applied a fixed concentration at 10%, which exceeded the minimum dosage needed for achieving 99%

repellency (ED_{99}) of all the five oils [39-41]. In addition, a 20% concentration, comparable to the concentration of standard DEET (positive control) was used for comparison [38]. These two doses were selected to establish a baseline for all five essential oils tested in this study, as well as providing sufficient chemical reservoir on the skin for measuring protection time. Two hundred nulliparous female mosquitoes (5-7 days old) were placed into a cage for treatment, and rested for about 2 hours to acclimatize. An additional 200 nulliparous females were placed in a separate cage for the control group to assess the duration of protection provided by each essential oil and their 1:1 combination. After the application of each desired oil concentration (1.67 $\mu L/cm^2$), both the treatment and control arms were exposed after 30 minutes, and this exposure continued at specified time intervals. The exposure was made for 3 minutes, then withdrawn for 30 minutes, and then exposed again. Any mosquito landing and biting were counted and scored. The experiment was stopped when the second mosquitoes landed on the test area and recorded as the complete-protection time. The testing period lasted up to 6 hours, depending on the efficacy. Three replicates were conducted for each test of concentrations with an identical test on different days using fresh mosquitoes.

Statistical analysis

The Statistical Package for the Social Sciences (SPSS) computer software version 25 was used for analyzing the data. The median effective dosage (ED_{50}) and ED_{99} of the essential oils were determined by probit plane regression analysis. Effectiveness of the test was determined by comparing the 95% confidence intervals of the ED_{50} and ED_{99} values. The complete protection time (CPT) for a given dose was estimated from the time elapsed up to the first confirmed mosquito landing and/or probing within 30 minutes. The median CPT and its confidence interval were estimated using the Kaplan–Meier survivor function procedure. After a non-parametric Kruskal-

Wallis H test of significant differences, Dunn's post hoc test was conducted to identify specific differences in the median protection time for each oil, as well as between the protection times of single and combined oils. Spearman's rank correlation coefficient (ρ) was also used to analyze the relationship between chemical composition and repellent effect of the oils using most abundant compounds (each >10%). The strength of the relationship was assessed based on the magnitude of ρ, where values close to 1.0 indicate a positive correlation trend and values near -1.0 indicate a negative correlation trend. The percentage of repellency was calculated by the formula [42].

$$\% \text{ Repellency} = \frac{C - T}{C} x100$$

where C = number of mosquitoes landing on control arm; and T = number of mosquitoes landing on the treated forearm.

Results

Yield of essential oils extracted from plant parts

The yields of essential oils extracted from plant parts are presented in Table 1. The highest yield of essential oil was obtained from the leaves of *E. globulus,* at *1.5*% (18 mL per 1200 g) followed by the leaves of *J. procera,* at 0.23% (2.75 mL per 1200 g) and the seeds of *C. macrostachyus,* at 0.18% (2.2 mL /1200 g). The lowest volume was obtained from the leaves of *C. macrostachyus*, yielding 0.07% (0.25 mL per 1200 g).

Chemical composition of essential oils

The results of the chromatographic analyses of the essential oils, including the compound retention time (RT), compound name, registry number (CAS), and percentage of compound content, are

presented in Table 2. A total of 162 chemical compounds were identified in the essential oils. The essential oils are mainly composed of monoterpenes, sesquiterpenes, alcohols and phenols.

In the essential oil of *E. globulus*, 28 compounds representing 100% of the total mixture were identified. The major constituents of this essential oil are 1,8-Cineole (42.50%) followed by delta-3-Carene (16.85%) and α-Terpinyl acetate (13.63). All three of these most abundant chemical compounds derived from *E. globulus* have been known for insect-repellent properties. Spearman's rank correlation analysis revealed the following correlation coefficients for these compounds: 1,8-Cineole ($\rho = -0.258$), Delta-3-Carene ($\rho = -1.00$), and α-Terpinyl Acetate ($\rho = -0.258$).

A total of fifty-two compounds, accounting for 98.23% of the total composition of *J. procera* essential oil, were identified. It mainly contained delta-3-Carene (32.04%) followed by 13-epi-manoyl oxide (11.10%). The correlation coefficient for 13-epi-manoyl oxide is ($\rho = -0.775$). This compound is a sesquiterpene and there are indications that some sesquiterpenes can exhibit repellent properties against mosquitoes and other insects.

The essential oil extracted from the seeds of *C. macrostachyus* contained a total of 32 compounds, accounting for 92.11% of the overall composition. The predominant compound identified was -gamma-Muurolene followed by Benzoic acid-phenylmethyl ester, which comprised 29.33% and 13.24% of the total mixture, respectively. Both of them have been known to have insect-repellent properties, including mosquitoes. Spearman's rank correlation analysis revealed the correlation coefficients for -gamma-Muurolene ($\rho = 0.316$) and Benzoic acid-phenylmethyl ester ($\rho = 0.775$).

A total of 50 compounds, representing 99% of the total composition of essential oil extracted from *E. kebericho*, were identified. The most abundant compound identified from this oil was

Dehydrocostus lactone (13.74%) with Spearman's rank correlation coefficients of (ρ =0.258). It is known for mosquito repellent activity.

Table 1. Amount of oil extracted from parts of plants that occur in Ghibe valley, southwest Ethiopia, 2025

Scientific name	Family name	Local name (Language)	Location coordinates	Parts used	Frequency (%, n=361)	Voucher number	Yield (%)
Croton macrostachyus	Euphorbiaceae	Wanshehna (Guragegna)	8.87868N 37.2312E	Leaves<br>Seeds	81	Z-13-2024	0.07<br>0.18
Echinops kebericho	Asteraceae	Kebercho (Amharic)	7.55885N 37.2803E	Root	58	Z-37-2024	0.17
Eucalyptus globulus	Myrtaceae	Nech Bahir Zaf (Amharic)	8.87925N 37.2384E	Leaves	56	Z-03-2024	1.5
Juniperus procera	Cuperssaceae	Yehabesh Tsid (Amharic)	7.69839N 37.7314E	Leaves	63	Z-09-2024	0.23

Table 2. Chemical composition of the essential oils, 2025

Essential oils	NO	RT	Compound name	CAS	Content (%)
E. globulus	**1**	**5.42**	**delta-3-Carene**	**13466-78-9**	**16.85**
	2	6.05	2(10)-Pinene	127-91-3	0.50
	3	6.22	1,6-Octadiene, 7-methyl-3-methylene-	123-35-3	1.33
	4	6.47	1,3-Cyclohexadiene, 2-methyl-5-(1-methylethyl)-	99-83-2	0.32
	5	6.78	Benzene, 1-methyl-4-(1-methylethyl)-	25155-15-1	0.22
	6	6.85	D-Limonene	5989-27-5	2.50
	**7**	**7.00**	**1,8-cineole**	**470-82-6**	**42.50**
	8	7.32	1,4-Cyclohexadiene, 1-methyl-4-(1-methylethyl)-	99-85-4	0.36
	9	7.77	4-Isopropylidene-1-cyclohexene	586-62-9	0.55
	10	9.05	Bicyclo [2.2.1] heptane, 2-chloro-1,7,7-trimethyl-, (1R-endo)-	30462-53-4	0.90
	11	9.24	Terpinen-4-ol	562-74-3	1.20
	12	9.48	α-Terpineol	98-55-5	2.57
	13	10.56	Lemonol	106-24-1	0.63
	14	12.43	2-Oxabicyclo [2.2.2] octan-6-ol, 1,3,3-trimethyl-, acetate	57709-95-2	0.24
	**15**	**12.64**	**α-Terpinyl acetate**	**80-26-2**	**13.63**
	16	13.26	Lavandulol acetate	25905-14-0	1.34
	17	14.04	. alpha. -Gurjunene	489-40-7	0.94
	18	14.75	Aromandendrene	489-39-4	2.93
	19	15.9	(-)-Dehydroaromadendrene	997220-88-5	0.56
	20	16.08	(+)-Leden	997221-46-4	0.90
	21	16.69	Nootkatene	5090-61-9	0.29
	22	17.91	(-)-Globulol	489-41-8	0.61
	23	18.17	(+)-Spathulenol	6750-60-3	0.31
	**24**	**18.38**	**Globulol**	**489-41-8**	**6.06**
	25	19.3	β-Eudesmol	473-15-4	0.93
	26	19.44	Aromandendrene	489-39-4	0.29
	27	19.73	Tau-Cadinol acetate	149197-48-8	0.27
	28	20.08	α-Eudesmol	473-16-5	0.28
J. procera	1	5.264	1,3-Cyclohexadiene, 2-methyl-5-(1-methylethyl)-	99-83-2	0.40
	2	5.594	(+)-Camphene	5794-03-6	2.52
	3	5.952	β-Cymene	535-77-3	0.32

	**4**	**6.056**	**2(10)-Pinene**	**127-91-3**	**4.66**
	**5**	**6.217**	**β-Myrcene**	**123-35-3**	**3.49**
	**6**	**6.616**	**delta-3-Carene**	**13466-78-9**	**32.04**
	7	6.789	β-Phellandrene	555-10-2	0.64
	**8**	**6.864**	**4(10)-Thujene**	**3387-41-5**	**3.31**
	9	7.297	γ-Terpinene	99-85-4	0.22
	**10**	**7.777**	**4-Isopropylidene-1-cyclohexene**	**586-62-9**	**6.23**
	11	7.915	Linalool	78-70-6	2.15
	12	8.528	trans-Verbenol	1820-09-3	0.54
	13	8.718	1,7,7-trimethylbicyclo [2.2.1] heptan-2-one	76-22-2	0.62
	14	9.042	(-)-(Z)-Verbenol	18881-04-4	0.21
	15	9.232	Terpinen-4-ol	562-74-3	0.80
	16	9.457	α-Terpineol	98-55-5	0.43
	17	9.798	cis-Carveol	1197-06-4	0.29
	18	10.65	D-Dihydrocarvone	7764-50-3	0.19
	**19**	**11.12**	**1,5-Cyclooctadiene, 3-(1-methyl-2-propenyl)-**	**16538-88-8**	**3.11**
	20	11.26	1,7,7-Trimethylbicyclo [2.2.1] hept-2-yl acetate	5655-61-8	1.30
	21	11.82	2,4-Decadienal, (E, E)-	25152-84-5	0.17
	22	12.08	Methyl 11,12-tetradecadienoate	997340-45-0	0.18
	23	14.29	Isocaryophyllene	87-44-5	2.64
	**24**	**15.09**	**1,4,7, -Cycloundecatriene, 1,5,9,9-tetramethyl-, Z, Z, Z-**	**997220-60-2**	**3.00**
	25	15.59	. gamma. -Muurolene	30021-74-0	0.56
	26	15.87	. beta. -Selinene	17066-67-0	0.18
	27	16.04	Aromandendrene	489-39-4	0.21
	28	16.53	γ-Cadinene	39029-41-9	0.31
	29	16.64	α-Copaen-11-ol	41370-56-3	0.18
	30	16.73	. delta. -Cadinene	483-76-1	0.88
	31	17.08	Guaia-6,9-diene	36577-33-0	0.35
	32	17.39	Elemol	639-99-6	1.24
	33	17.54	(1R,7S, E)-7-Isopropyl-4,10-dimethylenecyclodec-5-enol	81968-62-9	0.20
	34	17.62	2-((2R,4aR,8aS)-4a-Methyl-8-methylenedecahydronaphthalen-2-yl) prop-2-en-1-ol	515-20-8	0.18
	35	18.32	Caryophyllene oxide	1139-30-6	0.62
	36	18.98	(1R,3E,7E,11R)-1,5,5,8-Tetramethyl-12-oxabicyclo [9.1.0] dodeca-3,7-diene	19888-34-7	0.46
	37	19.5	gamma-eudesmol	1209-71-8	0.58

	38	20	beta-eudesmol	473-15-4	0.16
	39	20.07	alpha-eudesmol	473-16-5	1.90
	40	20.79	. gamma. -Cadinene	30021-74-0	0.36
	41	23.49	8.alpha.,11-Elemadiol	64373-81-5	2.63
	**42**	**28.37**	**13-epi-manoyl oxide**	**596-84-9**	**11.10**
	43	29.78	Dehydroabietane	19407-28-4	0.18
	44	30.37	decahydrophenanthrene	35241-40-8	1.78
	45	30.9	Kolavelool	19941-81-2	0.54
	46	31.71	(+)-Cembrene	1898-13-1	0.51
	47	32.63	Sandaracopimaral	3855-14-9	0.31
	48	34.15	Gestonorone -H2O P898	996002-07-5	0.52
	49	34.28	Pregn-16-en-20-one, 3-(acetyloxy)-, (3. beta.,5. beta.)-	2601-07-2	0.23
	50	34.45	Abieta-7,13-dien-18-al	6704-50-3	1.38
	51	34.62	trans-Totarol, Podocarpa-8,11,13-trien-13-ol, 14-isopropyl-	511-15-9	1.04
	52	34.82	Ferruginol	514-62-5	0.18
C. macrostachyus (seeds)	1	6.05	2(10)-Pinene	127-91-3	0.85
	2	7.916	1,6-Octadien-3-ol, 3,7-dimethyl-	78-70-6	2.45
	3	12.35	Cyclohexene, 4-ethenyl-4-methyl-3-(1-methylethenyl)-1-(1-methylethyl)-, (3R-trans)-	20307-84-0	0.72
	4	12.62	. alpha. -Cubebene	17699-14-8	1.37
	5	12.74	Aciphyllene	87745-31-1	1.00
	6	13.27	Copaene	3856-25-5	0.47
	**7**	**13.58**	**Aromadendrene**	**489-39-4**	**4.13**
	8	13.85	(1S,5S)-2-Methyl-5-((R)-6-methylhept-5-en-2-yl) bicyclo[3.1.0]hex-2-ene	159407-35-9	0.48
	9	14.34	1,1,7-Trimethyl-4-methylenedecahydro-1H-cyclopropa[e]azulene	489-39-4	0.37
	**10**	**14.5**	**beta-Copaene**	**18252-44-3**	**6.25**
	11	14.8	(1R,3aS,8aS)-7-Isopropyl-1,4-dimethyl-1,2,3,3a,6,8a-hexahydroazulene	36577-33-0	0.63
	12	15.11	. beta. -Bisabolene	495-61-4	0.47
	**13**	**15.91**	**(-)-Germacrene D**	**23986-74-5**	**4.68**
	**14**	**16.17**	**. gamma. -Muurolene**	**30021-74-0**	**29.33**
	15	16.26	. alpha. -Muurolene	10208-80-7	2.45
	16	16.37	. alpha. -Farnesene isomer [3,7,11-Trimethyl-1,3,6,10-dodecatetraene]	502-61-4	2.46
	**17**	**16.64**	**gamma-Cadinene**	**39029-41-9**	**4.32**
	**18**	**16.88**	**. delta. -Cadinene**	**483-76-1**	**4.15**
	19	17.05	Naphthalene, 1,2,3,4,4a,7-hexahydro-1,6-dimethyl-4-(1-methylethyl)-	16728-99-7	0.42

	20	17.17	alpha-cadinene	24406-05-1	1.36
	21	17.73	1,6,10-Dodecatrien-3-ol, 3,7,11-trimethyl-, [S-(Z)]-	7212-44-4	2.33
	22	18.14	Cubebol	23445-02-5	1.35
	23	18.39	(1R,7S, E)-7-Isopropyl-4,10-dimethylenecyclodec-5-enol	81968-62-9	0.54
	24	19.02	(1S,2E,6E,10R)-3,7,11,11-Tetramethylbicyclo [8.1.0] undeca-2,6-diene	24703-35-3	0.31
	25	19.77	. tau. -Cadinol	5937-11-1	1.21
	26	20.11	. tau. -Muurolol	19912-62-0	1.52
	27	20.83	. gamma. -Cadinene	30021-74-0	1.86
	28	21.45	Tridecanal	10486-19-8	0.31
	**29**	**23.00**	**Benzoic acid- phenylmethyl ester**	**120-51-4**	**13.24**
	30	24.00	Hexadecanal	629-80-1	0.37
	31	25.58	Cyclohexadecane P489	295-65-8	0.39
	32	28.15	Trachylobane	5282-35-9	0.32
E. kebericho	1	5.26	1,3-Cyclohexadiene, 2-methyl-5-(1-methylethyl)-	99-83-2	0.70
	**2**	**5.40**	**delta-3-Carene**	**13466-78-9**	**4.56**
	**3**	**6.08**	**2(10)-Pinene**	**127-91-3**	**4.20**
	4	6.21	1,6-Octadiene, 7-methyl-3-methylene-	123-35-3	0.18
	5	6.77	Benzene, 1-methyl-4-(1-methylethyl)-	25155-15-1	0.15
	6	6.86	2,6-Octadien-1-ol, 3,7-dimethyl-, acetate, (E)-	141-12-8	2.01
	7	7.30	1,4-Cyclohexadiene, 1-methyl-4-(1-methylethyl)-	99-85-4	0.55
	8	7.77	4-Isopropylidene-1-cyclohexene	586-62-9	0.19
	9	7.91	Bicyclo[2.2.1]heptane, 2,2-dimethyl-3-methylene-	79-92-5	0.22
	10	8.70	Verbenol P294	1845-30-3	0.59
	11	9.04	p-Mentha-1,5-dien-8-ol	1686-20-0	0.41
	12	9.24	3-Cyclohexen-1-ol, 4-methyl-1-(1-methylethyl)-	562-74-3	0.39
	13	11.28	1,7,7-Trimethylbicyclo [2.2.1] hept-2-yl acetate	5655-61-8	1.95
	14	12.14	Silphiperfol-5-ene	138752-24-6	0.85
	15	12.34	Longifolene-(V4)	61262-67-7	3.52
	16	12.61	Silphinene	74284-57-4	0.17
	17	13.06	(+)-Cycloisosativene	22469-52-9	2.08
	**18**	**13.31**	**Pethylbrene**	**442900-37-0**	**8.89**
	**19**	**13.47**	**Modephene**	**68269-87-4**	**8.59**
	20	13.63	(1R,3aS,5aS,8aR)-1,3a,4,5a-Tetramethyl-1,2,3,3a,5a,6,7,8-octahydrocyclopenta[c]pentalene	65372-78-3	0.54

	**21**	**13.79**	**Benzene, 1,2-dimethoxy-4-(2-propenyl)-**	**93-15-2**	**6.12**
	22	14.10	(1R,3aS,5aS,8aR)-1,3a,5a-Trimethyl-4-methylenedecahydrocyclopenta[c]pentalene	71596-72-0	2.84
	23	14.33	1,1,7-Trimethyl-4-methylenedecahydro-1H-cyclopropa[e]azulene	489-39-4	0.17
	24	14.80	(1R,3aS,8aS)-7-Isopropyl-1,4-dimethyl-1,2,3,3a,6,8a-hexahydroazulene	36577-33-0	0.14
	25	15.00	(-)-1,2,2. alpha.,3,3,4,6,7,8,8. alpha. -decahydro-2. alpha.,7,8-trimethylacenaphthylene	997220-90-9	1.50
	26	15.11	1,4,7, -Cycloundecatriene, 1,5,9,9-tetramethyl-, Z, Z, Z-	997220-60-2	0.86
	27	15.59	. alpha. -Gurjunene	489-40-7	0.62
	28	15.85	trans-. beta. -Ionone	79-77-6	2.93
	29	15.93	. beta. -Selinene	17066-67-0	2.12
	30	16.13	2-Isopropenyl-4a,8-dimethyl-1,2,3,4,4a,5,6,8a-octahydronaphthalene	207297-57-2	0.72
	**31**	**16.41**	**Naphthalene, 1,2,3,5,6,8a-hexahydro-4,7-dimethyl-1-(1-methylethyl)-, (1S-cis)-**	**483-76-1**	**9.80**
	**32**	**16.68**	**(3S,6S)-6-Isopropyl-3-methyl-2-(propan-2-ylidene)-3-vinylcyclohexanone**	**21698-46-4**	**3.69**
	33	16.79	. delta. -Cadinene	483-76-1	0.57
	34	18.14	1.beta.,4. beta.H,10. beta.H-Guaia-5,11-diene	22567-17-5	2.47
	35	18.38	Caryophyllene oxide	1139-30-6	0.20
	36	18.60	. gamma. -Gurjunenepoxide-(2)	184705-51-9	0.77
	37	18.69	. alpha. -dimethyl-. alpha. -ionone	0-00-0	0.27
	38	18.86	1H-Benzocyclohepten-7-ol, 2,3,4,4a,5,6,7,8-octahydro-1,1,4a,7-tetramethyl-, cis-	6892-80-4	0.84
	39	19.06	1.beta.,4. beta.H,10. beta.H-Guaia-5,11-diene	22567-17-5	1.51
	40	19.76	. tau. -Cadinol	5937-11-1	0.30
	41	20.00	2,6,10,10-Tetramethylbicyclo [7.2.0] undeca-1,6-diene	87-44-5	2.24
	42	20.12	. alpha. -Cadinol	481-34-5	1.87
	43	20.29	2-((2R,4aR,8aS)-4a-Methyl-8-methylenedecahydronaphthalen-2-yl) prop-2-en-1-ol	515-20-8	0.86
	44	20.86	7-Isopropenyl-1,4a-dimethyl-4,4a,5,6,7,8-hexahydro-3H-naphthalen-2-one	473-08-5	0.44
	45	20.95	alpha-Costic aldehyde	4586-01-0	0.25
	46	21.44	Humulenol-II	19888-00-7	0.19
	447	24.09	(1S,7S,8aR)-1,8a-Dimethyl-7-(prop-1-en-2-yl)-1,2,3,7,8,8a-hexahydronaphthalene	190327-38-9	0.43
	48	25.48	2-((2R,4aR,8aS)-4a-Methyl-8-methylenedecahydronaphthalen-2-yl) prop-2-en-1-ol	515-20-8	0.43
	49	27.13	Dihydrodehydrocostus lactone	4955-03-7	0.51
	**50**	**28.66**	**Dehydrocostus lactone**	**477-43-0**	**13.74**

Dose-dependent repellent effects of essential oils

In dose-response experiment, the five essential oils at a rate of 1.67µL/cm2 demonstrated repellent effects against *An. arabiensis bites*, as shown in Table 3. The essential oils obtained from both leaves and seeds of *C. macrostachyus* had the lowest ED_{50} (0.45%; 95%CI=0.39-0.51) and ED_{99} (2.48%; 95%CI=2.29-2.73) values, as well as ED_{50} (0.48%; 95%CI=0.41-0.53) and ED_{99} (2.50%; 95%CI=2.31-2.77). The oil from *J. procera* had the lowest repellent activity, with ED_{50} (0. 97%; 95% CI=0.92-1.06) and ED_{99} (4.83%; 95% CI=4.55-5.16).

Table 3. The effective dose of essential oils corresponding to 50% (ED_{50}) and 99% (ED_{99}) protection from mosquito landing against laboratory reared *Anopheles arabiensis*, 2025

Essential oils	ED_{50} (95%CI)	ED_{99} (95%CI)	X^2 (df=3)	p-value
C. macrostachyus (leaves)	0.45 (0.39-0.51)	2.48 (2.29-2.73)	6.04	0.109
C. macrostachyus (seeds)	0.48 (0.41-0.53)	2.50 (2.31-2.77)	5.94	0.101
E. kebericho	0.96 (0.94-1.02)	4.10 (3.89-4.36)	7.46	0.059
E. globulus	0.83 (0.79-0.87)	3.87 (3.64-4.14)	3.67	0.299
J. procera	0. 97 (0.92-1.06)	4.83 (4.55-5.16)	7.25	0.064

Repellency duration of the essential oils

Essential oils isolated from *C. macrostachyus* leaves and seeds provided protection for up to 180 minutes, with a percentage repellency of 87-100%. After 210 minutes, the protection decreased to 47–53%, and after 270 minutes, it reached 0 at 20% concentration. While *J. procera* essential oil provided the shortest protection, providing 93-100% protection after 30 minutes post-application and decreasing to 70-80% by 60 minutes. DEET, a standard commercial repellent, gave

significantly greater repellency, up to 93-100% for as long as 330 minutes of the test duration against *An. arabiensis* (Fig 1).

All the oils showed repellent activity against *An. arabiensis* providing 80-100% protection for 30 minutes at 10% concentration (Fig 2). Both oils from *C. macrostachyus* seeds/leaves protected for up to 150 minutes with a percentage repellency of 86-93%, and *E. kebericho* for 60 minutes with 86-93% percentage repellency, while *J. procera* oil gave the weakest protection, with 53% and lower protection for 60 minutes post application. DEET, the standard commercial repellent, provided substantially longer repellency (84-100% for up to 330 minutes post-application).

Fig 1. Percentage repellency provided by the essential oils at 20% test concentration and the standard DEET against laboratory reared *Anopheles arabiensis*, 2025

Fig 2. Percentage repellency provided the essential oils at 10% concentration and the standard DEET against laboratory reared *Anopheles arabiensis*, 2025

Comparisons of the duration of repellency among the five oils and DEET using a non-parametric Kruskal-Wallis H test indicated a significant difference ($P < 0.001$) at both 10% and 20% concentrations. A pairwise analysis of the five oils using Dunn's post hoc test, after a non-parametric Kruskal-Wallis H test, significant differences revealed that oils of both the leaves and seeds of *C. macrostachyus* as well as *E. kebericho* have a significantly greater length of protection time compared to the oils of *E. globulus* and *J. procera*. However, there was no significant difference between them. Median time of protection in minutes followed by the same letter showed no significantly different value in Dunn's post hoc test ($P > 0.05$) (Table 4).

Table 4. Median time of protection of the oils and a DEET against laboratory reared *Anopheles arabiensis*, 2025

Essential oil formulations	Median protection (minutes) at 10%.	Range (min-max)	Median protection (minutes) at 20%.	Range (min-max)
DEET (Positive Control)	390^{a}	270 – 390	362.00^{a}	330 – 390
C. macrostachyus (leaves)	152^{b}	150 – 153	211^{b}	153 – 241
C. macrostachyus (seeds)	141^{b}	93 – 153	214^{b}	151 – 270
E. kebericho	92^{b}	62 –151	151^{b}	91 – 153
E. globulus	62^{c}	31 – 91	91^{c}	33 – 152
J. procera	33^{c}	31 – 63	61^{c}	32 – 91

Duration of repellent effects from blended essential oils

All the combination of essential oils produced significant protection soon after the application. However, combination of *C. macrostachyus*+*E. kebericho* and *J. procera*+*C. macrostachyus* gave the longest mean duration of protection lasting 90 minutes with greater than 80% protection against *An. arabiensis* (Fig 3).

According to statistical comparison using a non-parametric Kruskal-Wallis H test, there was a significant difference ($P < 0.001$) in the median time of repellency between the binary combinations of oils and a DEET. In pairwise comparisons using Dunn's post hoc test, following a non-parametric Kruskal-Wallis H test, significant differences two of the six blended oils *C. macrostachyus* (seeds) + *E. kebericho* and *J. procera* + *C. macrostachyus* (seeds) showed significantly longer median times of protection. Median time of protection in minutes followed by the same letter showed no significantly different value in Dunn's post hoc test ($P > 0.05$) (Table 5).

The comparisons of protection time between the single and blended oils using a non-parametric Kruskal-Wallis H test showed that there are significant differences between them ($P < 0.001$). The result of pairwise comparisons using Dunn's post hoc test, following a non-parametric Kruskal-Wallis H test, significant differences indicated that blending with *C. macrostachyus*

(seeds) or *E. kebericho* significantly improves repellent efficacy compared to *J. procera* alone (P < 0.001), but other combinations preserve the repellent efficacy of the stronger component (P > 0.05). Median time of protection in minutes followed by the same letter showed no significantly different value in Dunn's post hoc test (P > 0.05) (Table 6).

Fig 3. Percentage repellency provided by the blended essential oils at 20% concentration and a DEET against laboratory reared *Anopheles arabiensis,* 2025

Table 5. Mean time of protection of the blended oils and DEET at 20% against laboratory reared *Anopheles arabiensis*, 2025

**Blended essential oil formulations**	**Median protection (minutes)**	**Range (min-max)**
DEET	390^{a}	330 – 390
C. macrostachyus (seeds) + *E. kebericho*	152^{b}	90 – 211
J. procera + *C. macrostachyus* (seeds)	150^{b}	61 – 153
E. globulus + *C. macrostachyus* (seeds)	93^{c}	33 – 212
E. globulus + *E. kebericho*	93^{c}	61 – 150
J. procera + *E. kebericho*	91^{c}	63 – 93
E. globulus + *J. procera*	91^{c}	32 – 150

Table 6. Comparisons of protection time between the single and blended oils at 20% against laboratory reared *Anopheles arabiensis*, 2025

**Single and blended oil formulations**	**Median protection (minutes)**	**Range (min-max)**
C. macrostachyus (seeds)	214^{a}	151 – 270
E. kebericho	151^{a}	91 – 153
E. globulus	91^{ab}	33 – 152
J. procera	61^{b}	32 – 91
C. macrostachyus (seeds) + *E. kebericho*	152^{a}	90 – 211

J. procera + *C. macrostachyus* (seeds)	150[a]	61 – 153
E. globulus + *C. macrostachyus* (seeds)	93[a]	33 – 212
E. globulus + *E. kebericho*	93[a]	61 – 150
J. procera + *E. kebericho*	91[a]	63 – 93
E. globulus + *J. procera*	91[ab]	32 – 150

Discussion

The present study evaluated the essential oils extracted from parts of selected plants such as *C. macrostachyus*, *E. kebericho*, *E. globulus,* and *J. procera* in the Ghibe valley for their repellency against *An. arabiensis* under laboratory condition. In this study, a high amount of essential oil was extracted from the leaves of *E. globulus.* Essential oil yields of fresh *E. globulus* in Ethiopia and elsewhere range from over 0.2% to 5% with varying degrees depending on location, season, and age, with younger leaves generally yielding more oil in line with the result of the current study [43]. The lowest oil was obtained from leaves of *C. macrostachyus* among the four plants, similar to the results of previous study [44]. Differences in the essential oil yields could be attributed to genetic factors, collection place, plant developmental stage, season of collection, and extraction methods [45, 46].

A total of 162 compounds were identified from the four essential oils assessed for their repellent properties in this study, using GC-MS-analysis. Of these, the most abundant compounds previously known to have repellent properties such as 1,8-Cineole, delta-3-Carene and α-Terpinyl acetate, 13-epi-manoyl oxide, -gamma-Muurolene, benzoic acid-phenylmethyl ester, and Dehydrocostus lactone were identified by the GC-MS-analysis [47, 48]. The predominant compounds such as -gamma-muurolene and benzoic acid-phenylmethyl ester, which are found in the *C. macrostachyus* seed oil, and dehydrocostus lactone in the *E. kebericho* root oil showed a

positive correlation trend with the repellent efficacy of the oils in varying strength. These findings suggest that achieving an optimal balance of these compounds is crucial for maximizing mosquito-repellent effects. All of them have been known to have insect-repellent properties, including mosquitoes, in the literature that supported the findings of the current study [47, 49, 50].

Other less abundant compounds identified from all the essential oils, like linalool and pinene which, previously reported to have repellent properties [51]. Beta- phellandrene that had previously reported as mosquito repellent properties was identified from *J. procera* oil [52]. Furthermore, in line with the previous study, all of the essential oils examined in this study are mainly composed of monoterpenes, sesquiterpenes, alcohols, and phenols that can be neurotoxic to mosquitoes [53].

The current investigation found that essential oils of the selected four plants, namely *C. macrostachyus*, *E. kebericho*, *E. globulus* and *J. procera,* have significant repellent properties with varying levels of effects against *An. arabiensis* bites. As a result, the findings of this study supported the findings of the earlier ethnobotanical study on the usage of these plants for the traditional control of mosquitoes and other arthropods in the Ghibe Valley, southwest Ethiopia [30]. The present study revealed that essential oil from the leaf and seed parts of *C. macrostachyus* showed promising repellent activity. A comparison of the five essential oils' ED_{50} values (dosage giving a 50% repellent effect) against *An. arabiensis* revealed that the repellent properties varied with dose. Among the five essential oils, the two extracted from the leaves and seeds of *C. macrostachyus* showed nearly the same repellent activities compared to each other. The ED_{50} of both oils of *C. macrostachyus* was lower compared to the five essential oils. These results demonstrated that oils from both the leaf and seed parts of *C. macrostachyus* had the strongest repelling efficacy. This may be attributed to the variations in the type and quantity of active

compounds present in the oil, which could influence repelling properties [54]. The scientific literature on the repellent efficacy of essential oils against *An. arabiensis* is often inconsistent [23]. These deviations often appear to directly correlate with the concentration of the essential oil tested and the type of solvent used to dilute the essential oil [55]. Furthermore, the chemical consistency of essential oils can be variable. Factors, such as the location, environmental conditions, specific plant species, mosquito species, and test subjects, may account for some of the inconsistencies [56]. Variation in the results of such type of repellency studies, including the current investigation, could come from differences in chemical profile, dosages, mosquito species, formulations, extraction, and test methods [52]. In some prior laboratory and field trials, the protection time was considered until mosquitoes landed, whereas in other studies, it was the time until mosquitoes bite [23, 57].

Among the five essential oils, both the leaf and seed essential oils of *C. macrostachyus* protected for more than 150 minutes at a 10% concentration and more than 180 minutes at a 20% concentration. The increased protection period of the oil at greater concentrations could be attributed to an increase in the concentration of the active ingredient contained in the oils. This demonstrates that increasing the concentration of essential oils can increase repellency, which is supported by the findings of several previous studies [33, 58]. The duration of protection exhibited by the essential oil of *C. macrostachyus* in the present study is longer than *O. suave, O. americanum, O. kilimandscharicum,* and *Lantana camara* against *An. gambiae,* which have shown less than an hour of complete protection in laboratory studies in Kenya [59]. However, the use of essential oils as insect repellents is highly limited by their fast volatility and oxidation rate [60]. The Environmental Protection Agency (EPA) of the United States has established that 120 minutes should be the standard minimum time required for a repelling effect of greater than 80% protection

in order to qualify for registration [61]. Hence, in line with this, the essential oils of both the leaves and seeds of *C. macrostachyus* provide promising protection against laboratory-reared *An. arabiensis* that meet the criteria for registration. However, field testing of these oils as much as possible in real-world conditions is required for registration. The evaluation of essential oil of *C. macrostachyus* for its repellency against *An. arabiensis* is the first in Ethiopia in spite of its wide use as herbal medicine by the indigenous people of tropical Africa [62]. A recent laboratory study in the country reported that the methanol crude extract and ethyl acetate fractions of *C. macrostachyus* leaves showed a promising larvicidal and adulticidal activity against *An. gambiae* [63].

Essential oil of *E. kebericho* protected the skin for more than 90 minutes at 20% concentrations. These findings support the traditional usage of *E. kebericho* as a mosquito repellent in the Ghibe valley, Ethiopia [30]. A previous study found that the essential oil of *E. kebericho* has strong repellent properties against non-blood-fed female *Anopheles arabiensis* at quite low concentrations. However, the duration of protection period was not specified [59]. The essential oil derived from *E. kebericho* has a longer duration of protection than oils of *O. suave, O. americanum, O. kilimandscharicum,* and *Lantana camara* against *An. gambiae* [59].

E. globulus and *J. procera* protected the skin for less than an hour in both 10% and 20% concentrations, with *E. globulus* protected for more than an hour at 20% concentration. Comparable result was reported by previous studies on essential oil of *E. globulus* in Ethiopia [64]. The complete protection time of *E. globulus* and *J. procera* was comparable *to O. suave*, *O. americanum, O. kilimandscharicum* and *Lantana camara An. gambiae* [59]. Previous similar study conducted in Ethiopia using the methanol extract of *J. procera* showed remarkable protection

against *An. arabiensis* [33]. It has also been reported that the degree of repellency could be improved significantly when the methanol extract of *J. procera* concentration is increased [33].

When comparing the repellent effects of the essential oil blends, the 1:1 binary combination of *C. macrostachyus* with *E. kebericho*, *J. procera* with *C. macrostachyus* (seeds) demonstrated a greater effectiveness than the remaining oil combinations. As a result, when two essential oils were combined, the kind, quantity, or ratio of active constituents changed [65]. This may lengthen or shorten the effective protection period against mosquito bites. When one essential oil is mixed with another, it may cause a synergistic or antagonistic reaction that change repelling properties. The result of the current study showed that blending with *C. macrostachyus* (seeds) or *E. kebericho* significantly improves repellent efficacy or possesses a strong synergistic effect compared to *J. procera* alone. This strong synergistic effect might be due to the heavy sesquiterpene lactones and esters found in *E. kebericho* and *C. macrostachyus* act as natural fixatives [66]. But other combinations preserve the repellent efficacy of the stronger component. These findings suggest that the majority of blended essential oils exhibited comparable repellency to single essential oils with slight differences. Therefore, the result implies that a 1:1 combination of most of the essential oils tested in this study did not cause a synergistic or antagonistic reaction. This lack of chemical interaction implies that the volatile compounds either target the same mosquito receptors or evaporate at their own individual, unchanged rates, resulting in no longer complete protection time (CPT) than single oils [40].

The shorter protection duration of the essential oils compared to DEET in the current study may suggest that there is faster loss of repellent activity due to faster volatilization of compounds of the essential oils [67]. To maximize the effectiveness of essential oils as repellent against mosquito bites, particular formulations might require. Adding 5% vanillin to *E. globulus* oil

increased its protection period against *Ae. albopictus* from 3-5 hours under laboratory conditions in China [68]. Citronella oil has long been used in commercial repellent formulations and is popular in India, however it is generally rated as less effective than repellents using synthetic active ingredients [69]. Despite the fact that all of the essential oils employed in this study are less effective than DEET, they are thought to be safer and can be used frequently.

Limitations of the study

The current study was limited by no bioassay was conducted with the major compounds of the essential oils to identify potent repellent chemicals against mosquitoes. Due to the small sample size, the correlation analysis investigated in this study may not accurately represent the relationships between the identified chemical compounds and mosquito-repellent effect, necessitating further research with a larger sample size. The study also did not evaluate mixtures of three or more oils, which may have limited an in-depth understanding from analyzing these aspects. Furthermore, the data did not include a safety assessment of the essential oils.

Conclusions

All of the five essential oils examined in this study had significant repellent properties against laboratory-reared *An. arabiensis* bites, but with different degrees of efficacy and shorter protection times than DEET. *C. macrostachyus* leaf and seed oils provided significantly longer protection against laboratory-reared *An. arabiensis* bites, lasting 180 minutes and 150 minutes at 20% and 10% concentrations, respectively. This meets the Environmental Protection Agency's registration criteria; thus, it should be studied further under field settings. Although plant essential oils are less effective than DEET, they are thought to be safer and can be used frequently. Evaluation of skin toxicity and the strategies for increasing the efficacy of repellents, such as purification of the active

ingredients, reduction in skin absorption, gradual release of the repellents, and increase in repellency persistence, must be also explored by future study.

Acknowledgments

The authors would like to acknowledge the Aklilu Lemma Institute of Health Research, Addis Ababa University, Ethiopia, for supporting us with field and laboratory materials. We are most grateful to the Center for Innovative Drug Development and Therapeutic Trials for Africa (CDT-Africa) for giving us permission and support for essential oil extractions. We thank Mr. Wosen Sisay and Mr. Jiregna Gemechu for their assistance during repellency test. We would like to thank Mr. Kebede Shenkute for his support in the identification of chemical compounds of essential oils at natural product chemistry laboratory, Adama Science and Technology University.

List of abbreviations

CDT-Africa	Center for Innovative Drug Development and Therapeutic Trials for Africa
ALIPB	Aklilu Lemma Institute of Pathobiology
SPSS	Statistical Package for the Social Sciences

Author Contributions

Conceptualization: Zeyede Teshome, Abebe Animut, Mirutse Giday, Esayas Aklilu.

Data curation: Zeyede Teshome.

Formal analysis: Zeyede Teshome.

Funding acquisition: Zeyede Teshome, Abebe Animut, Mirutse Giday, Esayas Aklilu.

Investigation: Zeyede Teshome, Abebe Animut, Belete Adefris Legesse, Mirutse Giday, Esayas Aklilu.

Methodology: Zeyede Teshome, Abebe Animut, Belete Adefris Legesse, Mirutse Giday, Esayas Aklilu.

Supervision: Zeyede Teshome, Abebe Animut, Mirutse Giday, Esayas Aklilu.

Validation: Zeyede Teshome, Abebe Animut, Mirutse Giday, Esayas Aklilu.

Visualization: Zeyede Teshome, Abebe Animut, Mirutse Giday, Esayas Aklilu.

Writing – original draft: Zeyede Teshome.

Writing – review & editing: Zeyede Teshome, Abebe Animut, Belete Adefris Legesse, Mirutse Giday, Esayas Aklilu.

References

1. Egwu CO, Aloke C, Chukwu J, Agwu A, Alum E, Tsamesidis I, et al. A world free of malaria: It is time for Africa to actively champion and take leadership of elimination and eradication strategies. African Health Sciences. 2022;22(4):627-40. https://doi.org/10.4314/ahs.v22i4.68 PMID: 37092107
2. Butler CD, Amegah AK, Wright CY, Bowles DC. Health, Climate and Challenges in Africa: 2024–2100. Climate Change and Global Health: Primary, Secondary and Tertiary Effects: CABI GB; 2024. p. 378-94. https://doi.org/10.1079/9781800620025.0031
3. Andrade MV, Noronha K, Guedes GR, Diniz BC, Silva VA, de Souza A, et al. Health-related quality of life due to malaria: a systematic review. Quality of Life Research. 2025;34(2):325-39. https://doi.org/10.1007/s11136-024-03822-w
4. Chen J, Tian X, Guo D, Gu H, Duan Y, Li D. Global trends and burdens of neglected tropical diseases and malaria from 1990 to 2021: a systematic analysis of the Global Burden of Disease Study 2021. BMC Public Health. 2025;25(1):1307. https://doi.org/10.1186/s12889-025-22477-x

5. Kendie FA, Hailegebriel W/kiros T, Nibret Semegn E, Ferede MW. Prevalence of Malaria among Adults in Ethiopia: A Systematic Review and Meta-Analysis. Journal of tropical medicine. 2021;2021(1):8863002. https://doi.org/10.1155/2021/8863002
6. WHO. Global Malaria Programme operational strategy 2024-2030: World Health Organization; 2024. https://www.who.int/publications/i/item/9789240090149.
7. Woldesenbet D, Tegegne Y, Mussema A, Tamene E, Mohamed K, Abebe W, et al. Can Ethiopia eliminate malaria? Malaria burden: insights from the pre-elimination era, current challenges and perspectives. Frontiers in Malaria. 2025;3:1492444. https://doi.org/10.3389/fmala.2025.1492444
8. Takken W, Charlwood D, Lindsay SW. The behaviour of adult Anopheles gambiae, sub-Saharan Africa's principal malaria vector, and its relevance to malaria control: a review. Malaria Journal. 2024;23(1):161. https://doi.org/10.1186/s12936-024-04982-3
9. Mwima R, Hui T-YJ, Nanteza A, Burt A, Kayondo JK. Potential persistence mechanisms of the major Anopheles gambiae species complex malaria vectors in sub-Saharan Africa: a narrative review. Malaria Journal. 2023;22(1):336. https://doi.org/10.1186/s12936-023-04775-0
10. Adugna F, Wale M, Nibret E. Review of Anopheles mosquito species, abundance, and distribution in Ethiopia. Journal of tropical medicine. 2021;2021(1):6726622. https://doi.org/10.1155/2021/6726622
11. Woyessa D, Yewhalaw D. Anopheles mosquito fauna, blood meal sources and transmission intensity from high and moderate malaria endemic areas of Ethiopia. Scientific Reports. 2025;15(1):10636. https://doi.org/10.1038/s41598-025-94739-y

12. Emiru T, Getachew D, Murphy M, Sedda L, Ejigu LA, Bulto MG, et al. Evidence for a role of Anopheles stephensi in the spread of drug-and diagnosis-resistant malaria in Africa. Nature medicine. 2023;29(12):3203-11. https://doi.org/10.1038/s41591-023-02641-9

13. Hawaria D, Amanuel T, Anbesie A, Zhong D, Kibret S, Lee M-C, et al. Elevated Plasmodium sporozoite infection rates in primary and secondary malaria vectors in Anopheles stephensi-infested areas of Ethiopia. Insects. 2025;16(5):462. **https://doi.org/10.3390/insects16050462**

14. Braack L, Macdonald MB. Outdoor biting by malaria vectors: what are the options for intervention? Malaria Journal. 2025. https://doi.org/10.1186/s12936-025-05689-9

15. Benelli G, Beier JC. Current vector control challenges in the fight against malaria. Acta tropica. 2017;174:91-6. https://doi.org/10.1016/j.actatropica.2017.06.028

16. Odero JI, Abong'o B, Moshi V, Ekodir S, Harvey SA, Ochomo E, et al. Early morning anopheline mosquito biting, a potential driver of malaria transmission in Busia County, western Kenya. Malaria journal. 2024;23(1):66. https://doi.org/10.1186/s12936-024-04893-3

17. Owuor KO. Resting Behaviour of African Malaria Vectors in an Era of High Indoor Insecticide Use: University of Nairobi; 2022.

18. Mukisa MC, Kassano JJ, Mwalugelo YA, Ntege C, Kahamba NF, Finda MF, et al. Analysis of the 24-h biting patterns and human exposures to malaria vectors in south-eastern Tanzania. Parasites & Vectors. 2024;17(1):445. https://doi.org/10.1186/s13071-024-06521-0

19. WHO. Global report on insecticide resistance in malaria vectors: 2010–2016. 2018. https://www.who.int/publications/i/item/9789241514057

20. Akuoko OK, Dhikrullahi SB, Hinne IA, Mohammed AR, Owusu-Asenso CM, Coleman S, et al. Biting behaviour, spatio-temporal dynamics, and the insecticide resistance status of malaria vectors in different ecological zones in Ghana. Parasites & Vectors. 2024;17(1):16. https://doi.org/10.1186/s13071-023-06065-9

21. Bedasso AH, Gutto AA, Waldetensai A, Eukubay A, Bokore GE, Kinde S, et al. Malaria vector feeding, peak biting time and resting place preference behaviors in line with Indoor based intervention tools and its implication: scenario from selected sentinel sites of Ethiopia. Heliyon. 2022;8(12). https://doi.org/10.1016/j.heliyon.2022.e12178

22. Sougoufara S, Ottih EC, Tripet F. The need for new vector control approaches targeting outdoor biting Anopheline malaria vector communities. Parasites & Vectors. 2020;13(1):295. https://doi.org/10.1186/s13071-020-04170-7

23. Asadollahi A, Khoobdel M, Zahraei-Ramazani A, Azarmi S, Mosawi SH. Effectiveness of plant-based repellents against different Anopheles species: a systematic review. Malaria journal. 2019;18(1):436. https://doi.org/10.1186/s12936-019-3064-8

24. Xue R-D, Zhao T-Y. Concepts of Best Management Practices for Intergrated Pest, Mosquito, and Vector Management. Bio-mathematics, Statistics, and Nano-Technologies: Chapman and Hall/CRC; 2023. p. 13-20. 10.1201/9781003035992-2.

25. Hazarika H, Krishnatreyya H. Technological Advancements in Mosquito Repellents: Challenges and Opportunities in Plant-Based Repellents. Acta Parasitologica. 2025;70(3):117. https://doi.org/10.1007/s11686-025-01054-7

26. Roy DN, Goswami R, Pal A. The insect repellents: A silent environmental chemical toxicant to the health. Environmental toxicology and pharmacology. 2017;50:91-102. https://doi.org/10.1016/j.etap.2017.01.019

27. Jiao X-Y, Wang C-S, Pan D, Liu P, Wang X-R, Zhang C, et al. Unveiling the potential health risks induced by photolysis of insect repellents DEET under simulated sunlight. Water Cycle. 2025;6:206-12. https://doi.org/10.1016/j.watcyc.2024.12.005

28. Yadav DK, Rathee S, Sharma V, Patil UK. A comprehensive review on insect repellent agents: medicinal plants and synthetic compounds. Anti-Inflammatory & Anti-Allergy Agents in Medicinal Chemistry. 2024. https://doi.org/10.2174/0118715230322355240903072704

29. Adimas ZT, Adimas MA, Abera BD. Plant-based bioactive compounds for grain storage: a comprehensive review. Cogent Food & Agriculture. 2024;10(1):2316152. https://doi.org/10.1080/23311932.2024.2316152

30. Teshome Z, Teka A, Animut A, Arage M, Aklilu E, Giday M. Ethnobotanical study of plants used for traditional control of mosquitoes and other arthropod pests in the Ghibe valley, southwest Ethiopia. Tropical Medicine and Health. 2025;53(1):1-27. https://doi.org/10.1186/s41182-025-00740-6

31. Pushpalatha E, Kocher DK, Prasad A, Bhowmik I, Anirudh E, Anoopkumar A, et al. Mosquito Control: Part II: Plant-Based Products or Phytochemicals. Mosquitoes of India: CRC Press; 2025. p. 170-83. 10.1201/9781003327028-15

32. Ríos J-L. Essential oils: What they are and how the terms are used and defined. Essential oils in food preservation, flavor and safety: Elsevier; 2016. p. 3-10. https://doi.org/10.1016/B978-0-12-416641-7.00001-8

33. Karunamoorthi K, Girmay A, Hayleeyesus SF. Mosquito repellent activity of essential oil of Ethiopian ethnomedicinal plant against Afro-tropical malarial vector Anopheles

arabiensis. Journal of King Saud University-Science. 2014;26(4):305-10. 10.1016/j.jksus.2014.01.001

34. Fikru S, Tolossa K, Lindemann P, Bucar F, Asres K. Larvicidal, ovicidal, and repellent activities of Leucas stachydiformis (Hochst. ex Benth.) Briq essential oil against Anopheles arabiensis. Journal of Tropical Medicine. 2024;2024(1):1051086. **https://doi.org/10.1155/2024/1051086**

35. Berhe M, Dugassa S, Shimelis S, Tekie H. Repellence and larvicidal effects of some selected plant extracts against adult Anopheles arabiensis and Aedes aegypti larvae under laboratory conditions. International Journal of Tropical Insect Science. 2021:1-8. https://doi.org/10.1007/s42690-021-00446-2

36. Adem AA, Belete A, Raorane ML, Junker BH, Neubert RH, Gebre-Mariam T. Formulation and evaluation of nanoemulgel loaded with essential oils with mosquito repellent activities. Journal of Drug Delivery Science and Technology. 2024;100:105991. 10.1016/j.jddst.2024.105991

37. Fiehn O, Kopka J, Trethewey RN, Willmitzer L. Identification of uncommon plant metabolites based on calculation of elemental compositions using gas chromatography and quadrupole mass spectrometry. Analytical chemistry. 2000;72(15):3573-80. 10.1021/ac991142i

38. WHO. Guidelines for efficacy testing of mosquito repellents for human skin. World Health Organization; 2009. https://www.who.int/publications/i/item/WHO-HTM-NTD-WHOPES-2009.4

39. Luker HA, Salas KR, Esmaeili D, Holguin FO, Bendzus-Mendoza H, Hansen IA. Repellent efficacy of 20 essential oils on Aedes aegypti mosquitoes and Ixodes scapularis ticks in

contact-repellency assays. Scientific reports. 2023;13(1):1705. https://doi.org/10.1038/s41598-023-28820-9

40. Lopez AD, Whyms S, Luker HA, Galvan CJ, Holguin FO, Hansen IA. Repellency of essential oils and plant-derived compounds against Aedes aegypti mosquitoes. Insects. 2025;16(1):51. **https://doi.org/10.3390/insects16010051**

41. Trongtokit Y, Rongsriyam Y, Komalamisra N, Apiwathnasorn C. Comparative repellency of 38 essential oils against mosquito bites. Phytotherapy Research: An International Journal Devoted to Pharmacological and Toxicological Evaluation of Natural Product Derivatives. 2005;19(4):303-9. **https://doi.org/10.1002/ptr.1637**

42. Yap H, Jahangir K, Chong A, Adanan C, Chong N, Malik Y, et al. Field efficacy of a new repellent, KBR 3023, against Aedes albopictus (SKUSE) and Culex quinquefasciatus (SAY) in a tropical environment. Journal of vector ecology: journal of the Society for Vector Ecology. 1998;23(1):62-8.

43. Nigussie G, Werede Y. Chemical profile and antimicrobial activity of essential oil of Eucalyptus globulus leaves from Haramaya campus and Entoto park, Ethiopia. Chemistry J. 2020;6:187-98.

44. Tariku Y, Hymete A, Hailu A, Rohloff J. Constituents, antileishmanial activity and toxicity profile of volatile oil from berries of Croton macrostachyus. Natural product communications. 2010;5(6):1934578X1000500635.

45. Mehalaine S, Chenchouni H. Quantifying how climatic factors influence essential oil yield in wild-growing plants. Arabian Journal of Geosciences. 2021;14(13):1257. https://doi.org/10.1007/s12517-021-07582-6

46. Dhotre I. A comprehensive review on progression and innovations in microwave assisted extraction technology for essential oils. Journal of Chemical Technology & Biotechnology. 2025;100(5):894-907.

47. Abdo BM, Asfaw BT, Choudhary MI. Bioassay-guided isolation of dehydrocostus lactone from Echinops kebericho as potential biopesticide against Aedes aegypti. Natural Product Research. 2025:1-7. https://doi.org/10.1080/14786419.2025.2486341

48. Klocke JA, Darlington MV, Balandrin MF. 1, 8-Cineole (Eucalyptol), a mosquito feeding and ovipositional repellent from volatile oil of Hemizonia fitchii (Asteraceae). Journal of chemical ecology. 1987;13(12):2131-41. https://doi.org/10.1007/BF01012562

49. Kamaraj C, Satish Kumar RC, Al-Ghanim KA, Nicoletti M, Sathiyamoorthy V, Sarvesh S, et al. Novel essential oils blend as a repellent and toxic agent against disease-transmitting mosquitoes. Toxics. 2023;11(6):517. **https://doi.org/10.3390/toxics11060517**

50. Feng Y, Chen J, Zhang A. Commercially available natural benzyl esters and their synthetic analogs exhibit different toxicities against insect pests. Scientific Reports. 2018;8(1):7902.

51. Tkachenko K, Varfolomeeva E. Prospects for the Use of Essential Oils as Repellants and/or Insecticides. Tropical Journal of Natural Product Research. 2022;6(6).

52. Jaenson TG, Pålsson K, Borg-Karlson A-K. Evaluation of extracts and oils of mosquito (Diptera: Culicidae) repellent plants from Sweden and Guinea-Bissau. Journal of medical entomology. 2006;43(1):113-9. https://doi.org/10.1093/jmedent/43.1.113

53. Wojtunik-Kulesza KA. Toxicity of selected monoterpenes and essential oils rich in these compounds. Molecules. 2022;27(5):1716. **https://doi.org/10.3390/molecules27051716**

54. Mapossa AB, Focke WW, Tewo RK, Androsch R, Kruger T. Mosquito-repellent controlled-release formulations for fighting infectious diseases. Malaria journal. 2021;20(1):165.

55. Krzyżowski M, Baran B, Łozowski B, Francikowski J. The role of dilution mediums in studies of fumigant insecticidal activity of essential oils. Journal of Pest Science. 2020;93(4):1119-24. https://doi.org/10.1007/s10340-020-01241-7

56. Mugao L. Factors influencing yield, chemical composition and efficacy of essential oils. Int J Multidiscip Res Growth Eval. 2024;5(4):169-78. https://doi.org/10.54660/.IJMRGE.2024.5.4.169-178

57. Soonwera M, Phasomkusolsil S. Efficacy of Thai herbal essential oils as green repellent against mosquito vectors. Acta tropica. 2015;142:127-30. https://doi.org/10.1016/j.actatropica.2014.11.010

58. Islam M, Hasan MM, Xiong W, Zhang S, Lei C. Fumigant and repellent activities of essential oil from Coriandrum sativum (L.)(Apiaceae) against red flour beetle Tribolium castaneum (Herbst)(Coleoptera: Tenebrionidae). Journal of Pest Science. 2009;82(2):171-7. https://doi.org/10.1007/s10340-008-0236-7

59. Seyoum A, Killeen GF, Kabiru EW, Knols BG, Hassanali A. Field efficacy of thermally expelled or live potted repellent plants against African malaria vectors in western Kenya. Tropical Medicine & International Health. 2003;8(11):1005-11.

60. Nerio LS, Olivero-Verbel J, Stashenko E. Repellent activity of essential oils: a review. Bioresource technology. 2010;101(1):372-8. 10.1016/j.biortech.2009.07.048

61. Novak RJ, Gerberg EJ. Natural-based repellent products: efficacy for military and general public uses. Journal of the American Mosquito Control Association. 2005;21(sp1):7-11.

62. Maroyi A. Ethnopharmacological uses, phytochemistry, and pharmacological properties of Croton macrostachyus Hochst. Ex Delile: a comprehensive review. Evidence-Based Complementary and Alternative Medicine. 2017;2017(1):1694671.

63. Tadesse S, Abay SM, Makonnen E, Ejigu A, Asemamaw Y, Haileselassie W, et al. Larvicidal and adulticidal effects of Ethiopian medicinal plants against Anopheles gambiae (Diptera: Culicidae). Malaria Journal. 2025;24(1):246. https://doi.org/10.1186/s12936-025-05443-1

64. Wano M. Evaluation of Essential oils of some local plants for their repellency against Anopheles arabiensis and Aedes aegypti. Addis Ababa University. 2006.

65. Hategekimana A, Erler F. Comparative repellent activity of single, binary and ternary combinations of plant essential oils and their major components against Sitophilus oryzae L.(Coleoptera: Curculionidae). Journal of Plant Diseases and Protection. 2020;127(6):873-81. https://doi.org/10.1007/s41348-020-00353-7

66. Lee MY. Essential oils as repellents against arthropods. BioMed research international. 2018;2018(1):6860271. **https://doi.org/10.1155/2018/6860271**

67. Farina P, Conti B. Liabilities of essential oils as insect repellents. Current Opinion in Environmental Science & Health. 2024;40:100564.

68. Wu W, Yang Y, Feng Y, Ren X, Li Y, Li W, et al. Study of the repellent activity of 60 essential oils and their main constituents against Aedes albopictus, and nano-formulation development. Insects. 2022;13(12):1077.

69. Sharma R, Rao R, Kumar S, Mahant S, Khatkar S. Therapeutic potential of citronella essential oil: a review. Current Drug Discovery Technologies. 2019;16(4):330-9. https://doi.org/10.2174/1570163815666180718095041

Supporting information

A representative total ion chromatogram (TIC) displaying the chemical profile of essential oils extracted by hydrodistillation. Major peaks are labeled based on the compounds listed in Table 2.

S1 file. GC-MS Chromatogram of essential oil of *Eucalyptus globulus*

S2 file. GC-MS Chromatogram of essential oil of *Juniperus procera*

S3 file. GC-MS Chromatogram of essential oil of *Croton macrostachyus*

S4 file. GC-MS Chromatogram of essential oil of *Echinops kebericho*

S5 file. Minimal datasets. Raw underlying data points for dose response study, and statistical analyses used to generate Table 3.

S6 file. Minimal datasets. Raw underlying data points for complete protection time study, and statistical analyses used to generate Figure 2 & Table 4.

S7 file. Minimal datasets. Raw underlying data points for complete protection time study, and statistical analyses used to generate Figure 1, Table 5 & 6.

S8 file. Minimal datasets. Raw underlying data points for correlation analysis between most abundant chemical compounds and repellent efficacy of essential oils.

Click here to access/download;Figure;Fig3.eps.pdf

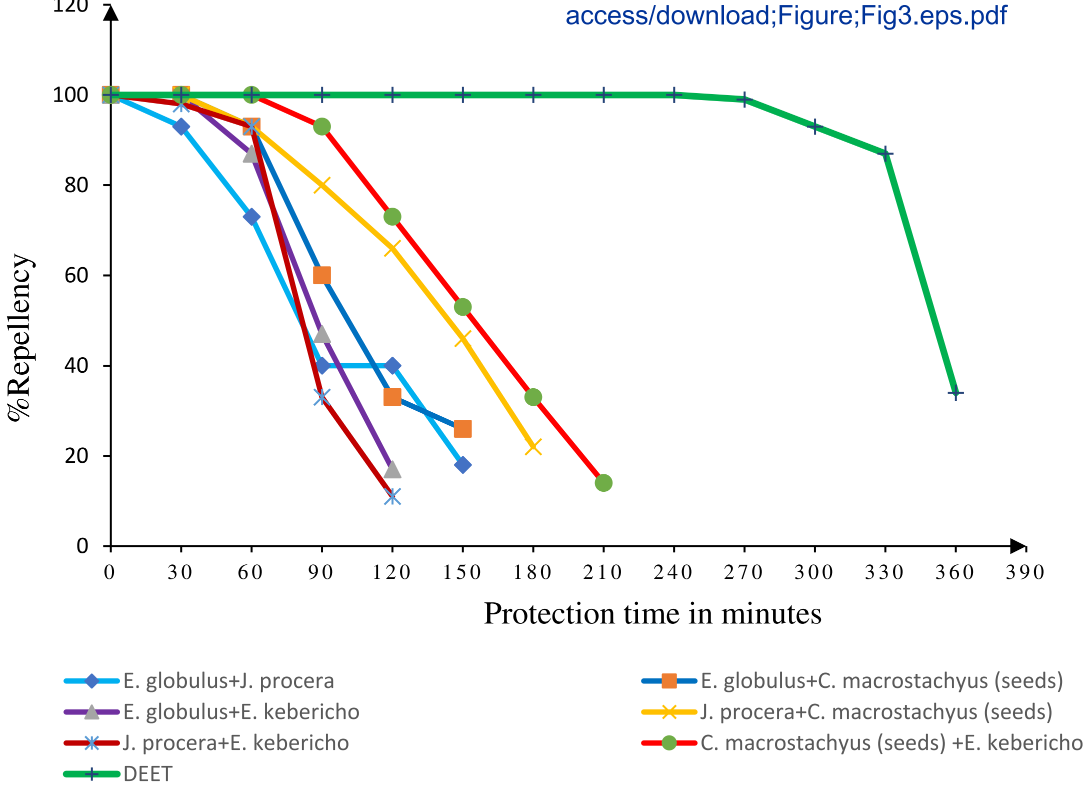

Click here to access/download;Figure;Fig1.eps.pdf

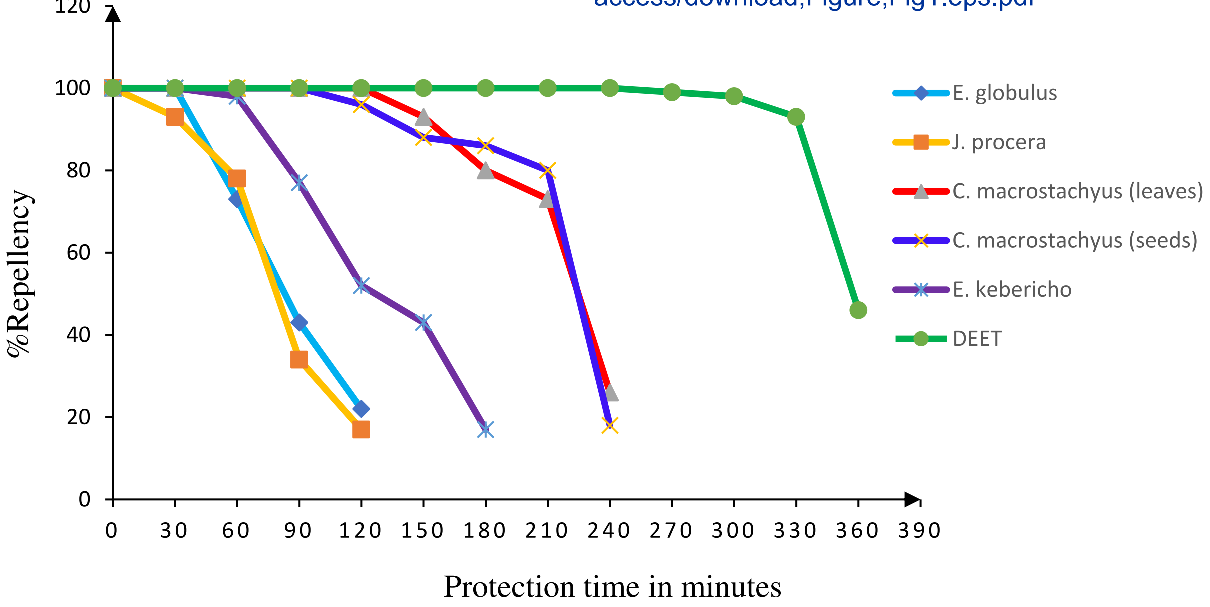

Click here to access/download;Figure;Fig2.eps.pdf

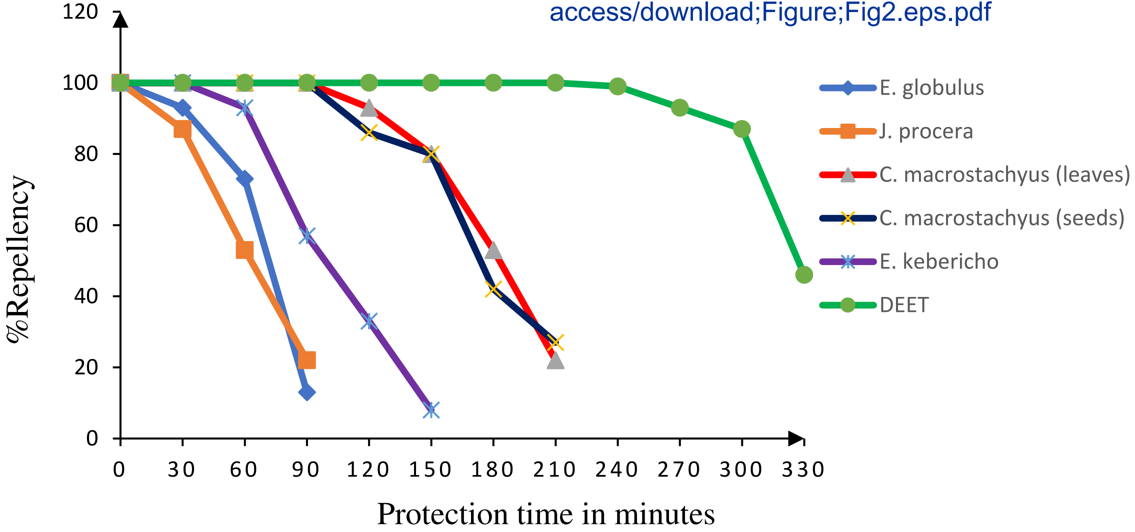

Click here to access/download
Supporting Information
S1_File.pdf.pdf

Click here to access/download
Supporting Information
S2_File.pdf.pdf

Click here to access/download
Supporting Information
S3_File.pdf.pdf

Click here to access/download
Supporting Information
S4_File.pdf.pdf

Click here to access/download
Supporting Information
S5_file. excel.xlsx

Click here to access/download
Supporting Information
S6_file.excel.xlsx

Click here to access/download
Supporting Information
S7_file.excel.xlsx

Click here to access/download

Supporting Information

S8_file.excel.xlsx

~~Repulsiveness~~ Repellency of selected essential oils against *Anopheles arabiensis* (Diptera: Culicidae) under laboratory condition

Formatted: Font: 18 pt

Zeyede Teshome[1,2*], Abebe Animut[2], Belete Adefris Legesse[3], Mirutse Giday[2], Esayas Aklilu[2]

[1]Department of Biology, Woldia University, P.O. box 50, Woldia, Ethiopia.

[2]Aklilu Lemma Institute of Health Research, Addis Ababa University, P O Box 1176, Addis Ababa, Ethiopia.

[3]Center for Innovative Drug Development and Therapeutic Trials for Africa (CDT-Africa), Addis Ababa University, P O Box 1176, Addis Ababa, Ethiopia.

*Correspondence:

Zeyede Teshome

zeyedetw21@gmail.com (Z.T.)

Abstract

Formatted: Font: 18 pt

The use of repellent provides human protection from mosquitoes and, hence, mosquito-borne diseases in light of the widespread shift in biting patterns. Plant-based repellents have traditionally been used to protect people from mosquitos. The current study~~,~~ aimed to assess the repellent effects of five essential oils against laboratory-reared *Anopheles arabiensis* bites, the major malaria vector in Ethiopia. Ethnobotanically investigated medicinal plants from the Ghibe Valley, southwest Ethiopia, namely *Croton macrostachyus*, *Echinops kebericho*, *Eucalyptus globulus* and *Juniperus procera* were used for extraction of essential oils. Essential oils were

extracted from the fresh plant parts using hydro-distillation methods. The chemical compositions of the essential oils were analyzed by Gas Chromatography-Mass Spectrometry (GC-MS). Laboratory arm-in-cage bioassays with human volunteers were conducted to estimate the effective dosage and evaluate the complete protection time at fixed concentrations of 10% and 20%. The data was analyzed by probit plane regression for dosage estimation, Kaplan–Meier and Kruskal-Wallis H tests for protection time, and Spearman's rank correlation coefficient (ρ) to correlate chemical compounds with repellent efficacy. There were 162 chemical compounds identified, including benzoic acid-phenylmethyl ester (ρ = 0.775) and -gamma-Muurolene (ρ = 0.316) which, were most abundant compounds and showed a positive correlation trend with repellency and are known to have insect repellent properties. The findings revealed that *C. macrostachyus* leaves and seed oils have a significantly longer protection time ($P < 0.001$), achieving 180 minutes with a mean protection of 85-100%. Blending *J. procera* oil with *C. macrostachyus* (seeds) or *E. kebericho* resulted in a significant synergistic repellent effect ($P < 0.001$). Other oil combinations maintained the repellent efficacy of the stronger component. The protection duration of *C. macrostachyus* leaf and seed oils meets the Environmental Protection Agency's registration criteria. However, further study is required under field conditions as well as a skin toxicity assessment.

Keywords: Essential oils, Ethiopia, Malaria, Medicinal plants, Mosquitoes, Repellency

Introduction

Formatted: Font: 18 pt

Malaria causes significant public health challenge despite decades of control efforts. It infected over 280 million people and claimed 600,000 lives in 2024 of which over 90% of the cases and deaths were from Africa (1, 2). About 75% of the population lives in high-endemic areas, 18% in

epidemic-prone and only 7% in low- or malaria-free (3, 4). The disease is the top cause of morbidity and mortality in Ethiopia (5, 6, 7).

Over 70 *Anopheles* species transmit malaria of which *Anopheles gambiae* s.s. and *An. arabiensis* are the most effective vectors, while *An. funestus*, *An. bwamnbe*, *An. merus*, and *An. melas* are involved in localized transmission in sub-Saharan Africa (8, 9). The predominant and most widespread vector in Ethiopia is *An. arabiensis* while *An. nili*, *An. pharoensis*, and *An. funestus* serve as secondary roles in limited settings (10, 11). *An. stephensi*, reported recently, is also a potential vector (12, 13).

Vector control remained the primary strategy to combat malaria (14, 15). The use of long-lasting insecticide-treated nets and indoor residual insecticide spraying hasve been effective to control malaria in indoor settings (14, 16, 17). However, these strategies are being challenged by vectors resistance to insecticides (18, 19), shift in biting from late-night to early-night and indoor to outdoor (20, 21). This entails the need for alternative control tools including mosquito repellents to suppress vectors regardless of their biting patterns (18, 22).

The use of repellents has been acknowledged in vector management (23, 24, 25). Repellents received substantial attention in recent years, largely because of increasing behavioral changes of mosquitoes and outdoor malaria transmission (22). Commercial repellents made from synthetic chemicals like N-diethyl-metatoluamide (DEET), allethrin, N-diethyl mendelic acid amide, and dimethyl phthalate are commonly used for protecting against mosquito bites (23). However, repeated application of synthetic repellents induces potential health concerns in humans disrupt natural ecosystems and poses ecotoxicological risks (26, 27). Thus, safe, economical and environment friendly repellent, including from plants, remains a priority (26, 28).`

The oils, smokes, tars, etc. have been employed to repel mosquitoes in Ethiopia, including the Ghibe valley, Ethiopia (29, 30). Plant based repellents received increased interest in recent years due to their concentrated bioactive phytochemicals that are both safe and biodegradable (26, 31). Essential oils of plants are complex mixtures of aromatic and chemically pure compounds that convey the unique fragrance and properties of plants that vary greatly by genetics, climate, rainfall and location (32). Previous studies in Ethiopia demonstrated essential oils of different plants with repellent properties against malaria vectors (33, 34, 35, 36). There are ethnobotanically claimed medicinal plants used to control mosquitoes and other insects in the Ghibe valley, southwest Ethiopia, previously identified and documented by our ethnobotanical survey (30). However, the repellency of essential oils derived from such traditionally/ethnobotanically claimed medicinal plants against *An. arabiensis,* the major malaria vector in Ethiopia remains scarce.~~,~~ The current study investigated the ~~repulsive~~ repellent properties of essential oils extracted from *Croton macrostachyus* Hochst. ex Delile, *Echinops kebericho* Mesfin, *Eucalyptus globulus* Labill. and *Juniperus procera* Hochst. ex Endl. against laboratory reared female *An. arabiensis* bite. Additionally, the chemical composition of the oils was assessed to better understand their efficacy and potential applications in mosquito repellency.

Materials and methods

Formatted: Font: 18 pt

Plant selection and collection

Formatted: Font: 16 pt

Plants selected on the basis of our previous ethnobotanical survey between March and October, 2024 in Deri Saja Zuria district, Enor, Misha, and Sekoru in the Ghibe Valley, southwest Ethiopia were evaluated (30). The plants were *C. ~~roton~~ macrostachyus* ~~Hochst. ex Delile~~, *E. ~~chinops~~*

kebericho ~~Mesfin~~, *E. ~~ucalyptus~~ globulus* ~~Labill.~~ and *J. ~~uniperus~~ procera* ~~Hochst. ex Endl~~. Plant parts were carefully collected in accordance with the local species protection and IUCN regulations, with less affected the wild populations in the areas between April and November 2025. Accordingly, fully developed leaves and seeds of *C. macrostachyus* ~~and~~ along with leaves of *E. globulus*, were collected from ~~its~~ their natural habitat in the Enor district.~~,~~ ~~leaves~~ Leaves of ~~*Juniperus*~~ *J. procera* were gathered from natural habitats in the Misha district, while roots of *E. kebericho* were harvested from local farmers in the Deri Saja Zuria district, where they are cultivated and prepared for the local market.

Essential oil extraction from plant parts

Formatted: Font: 16 pt

Essential oils were extracted by hydro-distillation processes using a Clevenger apparatus at natural product laboratory of CDT Africa, Addis Aba~~e~~ba University, using two distillation flasks with a capacity of 600 grams. Fresh plant parts (1200 g each) were cut into small pieces and placed in a distillation flask (600 g per flask) with enough water (3000 mL per flask) added. The distillation chamber was heated at about 80-100°C~~^{0}c~~ and allowed to boil for more than three hours. The distillate was collected in a separating funnel in which the aqueous portion was separated from the volatile oil. The water layer was slowly drawn off until only the oil layer would remain. The oil was collected in a 10ml glass bottle and stored at 4°C~~^{0}C~~ until it used for the chemical analysis and repellency test against *Anopheles arabiensis* bite.

Gas chromatography/mass spectrometry (GC/MS) analysis

Formatted: Font: 16 pt

The essential oils of *E. globulus, J. procera, C. macrostachyus* and *E. kebericho* were analyzed using GC-MS at the laboratory of natural product chemistry, Adama Science and Technology University. GC-MS analysis was performed using a GC (7890B, Agilent Technologies, China)

coupled with an MS (5977B Network, Agilent Technologies and USA). The GC had an HP-5MS column (non-polar column, Agilent Technologies), 30 m × 250 µm internal diameter and 0.25 µm film thickness. The carrier gas was helium flowing at a rate of 1 mL/minute. 1µL sample was injected, and the injector temperature was adjusted to 250°C and the injection mode was split mode with a split ratio of 50:1. The initial oven temperature was programmed from 50˚C, held for 1 minute. It was raised to 120˚C at 10˚C/minute and then ramped by 4˚C/minute to reach 220˚C, and finally raised to 280 with the rate of 20˚C/minute at this temperature held for 10 minutes. Mass spectra were recorded in EI mode at 70 eV, scanning the 45-550 m/z range. Percentage of each compound was calculated by area of a specific compound divided by the sum area of all identified compounds and multiplied by 100 (37).

Ethics approval and consent to participate

Formatted: Font: 16 pt

The Institutional Research Ethics Review Committee (ALIPB-IRERC) of Aklilu Lemma Institute of Health Research, Addis Ababa University, approved the study and issued an ethical clearance certificate (reference number ALIPB-IRERC/130/2016/24). Written permission letters for field access to all plant collection sites were obtained from the Institute of Public Health in the Central Ethiopia Regional State, as well as from the health offices of each study district. Volunteers provided written informed consent on 13 June 2025 after receiving explanations of the procedures and benefits of the study. This consent was obtained prior to conducting each laboratory test of repellency using the arm-in-cage method, which took place from June 15 to November 30, 2025.

Evaluating repellency of essential oils

Formatted: Font: 16 pt

The repellency of essential oils derived from various plant parts, namely *C. macrostachyus* (leaves and seeds), *E. kebericho* (roots), *E. globulus* (leaves) and *J. procera* (leaves) was evaluated against a laboratory colony of female *An. arabiensis*, which was maintained at the Aklilu Lemma Institute of Health Research, Addis Ababa University following the WHO guidelines (38). Each essential oil was diluted in ethanol to prepare a range of serial dilutions at various concentrations. Thus, five different v/v concentrations, such as 10 µL oil in 1mL ethanol (1%), 25 µL oil per1 mL ethanol (2.5%), 50 mL oil per 1 mL ethanol (5%), 75 µL oil per 1mL ethanol (7.5%) and 100 µL oil per 1 mL ethanol (10%) were prepared for the dose response test. Other Additional concentrations, such as 100 µL oil per 1 mL ethanol (10%) and 200 µL oil per 1 mL of ethanol (20%), were prepared to evaluate the protection duration of each essential oil. Additionally, a total of six binary combinations of the essential oils were created, including *globulus* + *J. procera, E. globulus* + *C. macrostachyus* (seeds), *E. globulus* + *E. kebericho, J. procera* + *C. macrostachyus* (seeds), *J. procera* + *E. kebericho*, and *C. macrostachyus* (seeds) + *E. kebericho*, all mixed in a 1:1 v/v ratio. The required concentration of 200 µL of oil per 1 mL of ethanol (20% v/v) for each combination was then prepared to evaluate the effects of the blended oils.

The test mosquitos were reared, maintained and tested in a separate room under optimal conditions. Mosquitoes were maintained at 27 ± 2°C^{0}C, 60 to 70% relative humidity, and had constant access to a 10% sugar solution. Nulliparous female mosquitoes were starved for 12 hours before the repellent test began. Standardized cages (30cm × 30cm × 30cm) were used for daytime testing in a dark room.

Five healthy adult human volunteers (>30 years old) with no history of allergic reaction to mosquito bites were selected to participate in the experiment. Participants were encouraged to

avoid alcohol, tobacco, and any scented products for at least 12 hours before the experiment. The amount of each test oil applied on the arm was determined from the surface area of the arm (in cm^2) of skin is then calculated according to the formula (38). Area = ½ ($C_w + C_e$) D_{we} where, C_w is the circumference of the wrist in cm, C_e is the elbow-cubital fossa circumference in cm, and D_{we} is the distance in between Ce and Cw. Accordingly, the calculated surface of the arm of the exposed volunteers were as follows (V1= 562.5 cm^2, V2= 516 cm^2, V3= 588 cm^2, V4= 572.5 cm^2, and V5= 546.8 cm^2).

Volunteers were required to clean their hands and arms thoroughly followed by drying. A surgical glove was then worn on the hand to prevent biting on the untreated hand. The test area of the exposed volunteer (the inner part of the forearm between the wrist and elbow) was washed with unscented soap and ethanol. Each test consisted of two parts: the right arm was treated with the essential oils, while the left arm served as the control. Each volunteer was exposed to each essential oil once per day. ~~Between~~ During the time intervals between ~~the~~ exposure periods, volunteers were instructed not to rub, touch, or wet the treated arms and were restricted to stay in ~~a~~ controlled room to minimize the loss of the oils from the treated forearms.

Estimation of effective dose: In this bioassay, a range of doses (1-10%) of all essential oils was tested to determine the effective dosage (ED_{50} and ED_{99}). One hundred nulliparous female mosquitoes (5-7 days old) were placed in a cage and allowed to acclimatize for approximately 2 hours. Their biting readiness was then tested by exposing only an ethanol-treated forearm (control arm) for up to 30 seconds. When at least 10 mosquitoes landed on the control arm within 30 seconds, the test continued. The control forearm was removed, and the desired concentration of the oils, with an amount of 1.67 µL/cm^2, was applied uniformly on the treatment arms of the volunteers. ~~The control forearm was removed and the desired~~

Formatted: Font: 14 pt

concentration of oil (0.86-0.98mL) was applied uniformly on treatment arms of the respective volunteers. The successive doses of oils were tested one after the other starting from the lowest concentration to the highest, until no landing was recorded. The repellent dose for each test was calculated as the sum of the doses applied, resulting in a cumulative dose for each test (38). To prevent the effect of evaporation, the interval between applications was minimum and exposure of each dose lasting 30 seconds. Each test concentration was repeated 3 times per volunteer in different days using fresh mosquitoes. The number of counts of mosquito landing was recorded and used in data analysis.

Duration of mosquito landing protection: In this bioassay, due to the highly volatile nature of the essential oils and vary significantly in their calculated ED_{99} values, they were applied a fixed concentration at 10%, which exceeded the minimum dosage needed for achieving 99% repellency (ED_{99}) of all the five oils (39, 40, 41). In addition, a 20% concentration, comparable to the concentration of standard DEET (positive control) was used for comparison (38). These two doses were selected to establish a baseline for all five essential oils tested in this study, as well as providing sufficient chemical reservoir on the skin for measuring protection time. Two hundred nulliparous female mosquitoes (5-7 days old) were placed into a cage for treatment, and rested for about 2 hours to acclimatize. An additional 200 nulliparous females were placed in a separate cage for the control group to assess the duration of protection provided by each essential oil and their 1:1 combination. After the application of each desired oil concentration (1.67 µL/cm²), both the treatment and control arms were exposed after 30 minutes, and this exposure continued at specified time intervals. After 30 minutes post application of each of the desired oil concentrations(0.86-0.98mL) on the treatment arm and the same volume of DEET (positive control) on the control arm, The exposure was made for 3 minutes, then

Formatted: Font: 14 pt

withdrawn for 30 minutes, and then exposed again. Any mosquito landing and biting were counted and scored. The experiment was stopped when the second mosquitoes landed on the test area and recorded as the complete-protection time. The testing period lasted up to 6 hours, depending on the efficacy. Three replicates were conducted for each test of concentrations with an identical test on different days using fresh mosquitoes.

Statistical analysis

Formatted: Font: 16 pt

The Statistical Package for the Social Sciences (SPSS) computer software version 25 was used for analyzing the data. The median effective dosage (ED_{50}) and ED_{99} of the essential oils were determined by probit plane regression analysis. Effectiveness of the test was determined by comparing the 95% confidence intervals of the ED_{50} and ED_{99} values. The complete protection time (CPT) for a given dose was estimated from the time elapsed up to the first confirmed mosquito landing and/or probing within 30 minutes. The median CPT and its confidence interval were estimated using the Kaplan–Meier survivor function procedure. After a non-parametric Kruskal-Wallis H test of significant differences, Dunn's post hoc test was conducted to identify specific differences in the median protection time for each oil, as well as between the protection times of single and combined oils. Spearman's rank correlation coefficient (ρ) was also used to analyze the relationship between chemical composition and repellent effect of the oils using most abundant compounds (each >10%). The strength of the relationship was assessed based on the magnitude of ρ, where values close to 1.0 indicate a positive correlation trend and values near -1.0 indicate a negative correlation trend. The percentage of repellency was calculated by the formula (42).

$$\%\ \text{Repellency} = \frac{C - T}{C} x100$$

where C = number of mosquitoes landing on control arm; and T = number of mosquitoes landing on the treated forearm.

Results

Formatted: Font: 18 pt

Yield of essential oils extracted from plant parts

Formatted: Font: 16 pt

The yields of essential oils extracted from plant~~s~~ parts are ~~given~~ presented in Table 1. The highest yield of essential oil was obtained ~~the~~from the leaves of ~~*Eucalyptus*~~ *E. globulus*, at ~~(~~1.5%~~;~~ (18 mL ~~1200~~per 1200 g) followed by the leaves of ~~*Juniperus*~~ *J. procera*, at ~~(~~0.23%~~;~~ (2.75 mL per 1200 g) and the seeds of *C. macrostachyus*, at ~~(~~0.18%~~;~~ (2.2 mL /1200 g). The lowest volume was obtained from the leaves of *C. macrostachyus*, yielding 0.07% (0.25 mL per 1200 g).

Chemical composition of essential oils

Formatted: Font: 16 pt

The results of the chromatographic analyses of the essential oils, including the compound retention time (RT), compound name, registry number (CAS), and percentage of compound content, are presented in Table 2. A total of 162 chemical compounds were identified ~~from~~ in the essential oils. The essential oils are mainly composed of monoterpenes, sesquiterpenes, alcohols and phenols.

In the essential oil of *E. globulus*, 28 compounds representing 100% of the total mixture were identified. The major constituents of this essential oil are 1,8-Cineole (42.50%) followed by delta-3-Carene (16.85%) and α-Terpinyl acetate (13.63). All three of these most abundant chemical compounds derived from *E. globulus* have been known for insect-repellent properties. Spearman's rank correlation analysis revealed the following correlation coefficients for these

compounds: 1,8-Cineole (ρ = -0.258), Delta-3-Carene (ρ = -1.00), and α-Terpinyl Acetate (ρ = -0.258).

A total of fifty-two compounds, accounting for 98.23% of the total composition of *J. procera* essential oil, were identified. It mainly contained delta-3-Carene (32.04%) followed by 13-epi-manoyl oxide (11.10%). The correlation coefficient for 13-epi-manoyl oxide is (ρ = -0.775). This compound is a sesquiterpene and there are indications that some sesquiterpenes can exhibit repellent properties against mosquitoes and other insects.

The essential oil extracted from the seeds of *C. macrostachyus* contained a total of 32 compounds, accounting for 92.11% of the overall composition. The predominant compound identified was -gamma-Muurolene followed by Benzoic acid-phenylmethyl ester, which comprised 29.33% and 13.24% of the total mixture, respectively. Both of them have been known to have insect-repellent properties, including mosquitoes. Spearman's rank correlation analysis revealed the correlation coefficients for -gamma-Muurolene (ρ = 0.316) and Benzoic acid-phenylmethyl ester (ρ = 0.775).

A total of 50 compounds, representing 99% of the total composition of essential oil extracted from *E. kebericho*, were identified. The most abundant compound identified from this oil was Dehydrocostus lactone (13.74%) with Spearman's rank correlation coefficients of (ρ =0.258). It is known for mosquito repellent activity.

Table 1. Amount of oil extracted from parts of plants that occur in Ghibe valley, southwest Ethiopia, 2025

Scientific name	Family name	Local name (Language)	Location coordinates	Parts used	Frequency (%, n=361)	Voucher number	Yield (%)
Croton macrostachyus	Euphorbiaceae	Wanshehna (Guragegna)	8.87868N 37.2312E	Leaves<br>Seeds	81	Z-13-2024	0.07<br>0.18
Echinops kebericho	Asteraceae	Kebercho (Amharic)	7.55885N 37.2803E	Root	58	Z-37-2024	0.17
Eucalyptus globulus	Myrtaceae	Nech Bahir Zaf (Amharic)	8.87925N 37.2384E	Leaves	56	Z-03-2024	1.5
Juniperus procera	Cuperssaceae	Yehabesh Tsid (Amharic)	7.69839N 37.7314E	Leaves	63	Z-09-2024	0.23

Formatted: Font: Bold

Formatted: Font: Bold

Formatted: Font: Not Italic

Formatted: Font: Not Italic

Formatted: Font: Not Italic

Formatted: Font: Not Italic

Table 2. Chemical composition of the essential oils, 2025

Formatted: Font: Bold

Essential oils	NO	RT	Compound name	CAS	Content (%)
E. globulus	**1**	**5.42**	**delta-3-Carene**	**13466-78-9**	**16.85**
	2	6.05	2(10)-Pinene	127-91-3	0.50
	3	6.22	1,6-Octadiene, 7-methyl-3-methylene-	123-35-3	1.33
	4	6.47	1,3-Cyclohexadiene, 2-methyl-5-(1-methylethyl)-	99-83-2	0.32
	5	6.78	Benzene, 1-methyl-4-(1-methylethyl)-	25155-15-1	0.22
	6	6.85	D-Limonene	5989-27-5	2.50
	**7**	**7.00**	**1,8-cineole**	**470-82-6**	**42.50**
	8	7.32	1,4-Cyclohexadiene, 1-methyl-4-(1-methylethyl)-	99-85-4	0.36
	9	7.77	4-Isopropylidene-1-cyclohexene	586-62-9	0.55
	10	9.05	Bicyclo [2.2.1] heptane, 2-chloro-1,7,7-trimethyl-, (1R-endo)-	30462-53-4	0.90
	11	9.24	Terpinen-4-ol	562-74-3	1.20
	12	9.48	α-Terpineol	98-55-5	2.57
	13	10.56	Lemonol	106-24-1	0.63
	14	12.43	2-Oxabicyclo [2.2.2] octan-6-ol, 1,3,3-trimethyl-, acetate	57709-95-2	0.24
	**15**	**12.64**	**α-Terpinyl acetate**	**80-26-2**	**13.63**
	16	13.26	Lavandulol acetate	25905-14-0	1.34
	17	14.04	. alpha. -Gurjunene	489-40-7	0.94
	18	14.75	Aromandendrene	489-39-4	2.93
	19	15.9	(-)-Dehydroaromadendrene	997220-88-5	0.56
	20	16.08	(+)-Leden	997221-46-4	0.90
	21	16.69	Nootkatene	5090-61-9	0.29
	22	17.91	(-)-Globulol	489-41-8	0.61
	23	18.17	(+)-Spathulenol	6750-60-3	0.31
	**24**	**18.38**	**Globulol**	**489-41-8**	**6.06**
	25	19.3	β-Eudesmol	473-15-4	0.93
	26	19.44	Aromandendrene	489-39-4	0.29
	27	19.73	Tau-Cadinol acetate	149197-48-8	0.27
	28	20.08	α-Eudesmol	473-16-5	0.28
J. procera	1	5.264	1,3-Cyclohexadiene, 2-methyl-5-(1-methylethyl)-	99-83-2	0.40
	2	5.594	(+)-Camphene	5794-03-6	2.52
	3	5.952	β-Cymene	535-77-3	0.32

	**4**	**6.056**	**2(10)-Pinene**	**127-91-3**	**4.66**
	**5**	**6.217**	**β-Myrcene**	**123-35-3**	**3.49**
	**6**	**6.616**	**delta-3-Carene**	**13466-78-9**	**32.04**
	7	6.789	β-Phellandrene	555-10-2	0.64
	**8**	**6.864**	**4(10)-Thujene**	**3387-41-5**	**3.31**
	9	7.297	γ-Terpinene	99-85-4	0.22
	**10**	**7.777**	**4-Isopropylidene-1-cyclohexene**	**586-62-9**	**6.23**
	11	7.915	Linalool	78-70-6	2.15
	12	8.528	trans-Verbenol	1820-09-3	0.54
	13	8.718	1,7,7-trimethylbicyclo [2.2.1] heptan-2-one	76-22-2	0.62
	14	9.042	(-)-(Z)-Verbenol	18881-04-4	0.21
	15	9.232	Terpinen-4-ol	562-74-3	0.80
	16	9.457	α-Terpineol	98-55-5	0.43
	17	9.798	cis-Carveol	1197-06-4	0.29
	18	10.65	D-Dihydrocarvone	7764-50-3	0.19
	**19**	**11.12**	**1,5-Cyclooctadiene, 3-(1-methyl-2-propenyl)-**	**16538-88-8**	**3.11**
	20	11.26	1,7,7-Trimethylbicyclo [2.2.1] hept-2-yl acetate	5655-61-8	1.30
	21	11.82	2,4-Decadienal, (E, E)-	25152-84-5	0.17
	22	12.08	Methyl 11,12-tetradecadienoate	997340-45-0	0.18
	23	14.29	Isocaryophyllene	87-44-5	2.64
	**24**	**15.09**	**1,4,7, -Cycloundecatriene, 1,5,9,9-tetramethyl-, Z, Z, Z-**	**997220-60-2**	**3.00**
	25	15.59	. gamma. -Muurolene	30021-74-0	0.56
	26	15.87	. beta. -Selinene	17066-67-0	0.18
	27	16.04	Aromandendrene	489-39-4	0.21
	28	16.53	γ-Cadinene	39029-41-9	0.31
	29	16.64	α-Copaen-11-ol	41370-56-3	0.18
	30	16.73	. delta. -Cadinene	483-76-1	0.88
	31	17.08	Guaia-6,9-diene	36577-33-0	0.35
	32	17.39	Elemol	639-99-6	1.24
	33	17.54	(1R,7S, E)-7-Isopropyl-4,10-dimethylenecyclodec-5-enol	81968-62-9	0.20
	34	17.62	2-((2R,4aR,8aS)-4a-Methyl-8-methylenedecahydronaphthalen-2-yl) prop-2-en-1-ol	515-20-8	0.18
	35	18.32	Caryophyllene oxide	1139-30-6	0.62
	36	18.98	(1R,3E,7E,11R)-1,5,5,8-Tetramethyl-12-oxabicyclo [9.1.0] dodeca-3,7-diene	19888-34-7	0.46
	37	19.5	gamma-eudesmol	1209-71-8	0.58

	38	20	beta-eudesmol	473-15-4	0.16
	39	20.07	alpha-eudesmol	473-16-5	1.90
	40	20.79	. gamma. -Cadinene	30021-74-0	0.36
	41	23.49	8.alpha.,11-Elemadiol	64373-81-5	2.63
	**42**	**28.37**	**13-epi-manoyl oxide**	**596-84-9**	**11.10**
	43	29.78	Dehydroabietane	19407-28-4	0.18
	44	30.37	decahydrophenanthrene	35241-40-8	1.78
	45	30.9	Kolavelool	19941-81-2	0.54
	46	31.71	(+)-Cembrene	1898-13-1	0.51
	47	32.63	Sandaracopimaral	3855-14-9	0.31
	48	34.15	Gestonorone -H2O P898	996002-07-5	0.52
	49	34.28	Pregn-16-en-20-one, 3-(acetyloxy)-, (3. beta.,5. beta.)-	2601-07-2	0.23
	50	34.45	Abieta-7,13-dien-18-al	6704-50-3	1.38
	51	34.62	trans-Totarol, Podocarpa-8,11,13-trien-13-ol, 14-isopropyl-	511-15-9	1.04
	52	34.82	Ferruginol	514-62-5	0.18
C. macrostachyus (seeds)	1	6.05	2(10)-Pinene	127-91-3	0.85
	2	7.916	1,6-Octadien-3-ol, 3,7-dimethyl-	78-70-6	2.45
	3	12.35	Cyclohexene, 4-ethenyl-4-methyl-3-(1-methylethenyl)-1-(1-methylethyl)-, (3R-trans)-	20307-84-0	0.72
	4	12.62	. alpha. -Cubebene	17699-14-8	1.37
	5	12.74	Aciphyllene	87745-31-1	1.00
	6	13.27	Copaene	3856-25-5	0.47
	**7**	**13.58**	**Aromadendrene**	**489-39-4**	**4.13**
	8	13.85	(1S,5S)-2-Methyl-5-((R)-6-methylhept-5-en-2-yl) bicyclo[3.1.0]hex-2-ene	159407-35-9	0.48
	9	14.34	1,1,7-Trimethyl-4-methylenedecahydro-1H-cyclopropa[e]azulene	489-39-4	0.37
	**10**	**14.5**	**beta-Copaene**	**18252-44-3**	**6.25**
	11	14.8	(1R,3aS,8aS)-7-Isopropyl-1,4-dimethyl-1,2,3,3a,6,8a-hexahydroazulene	36577-33-0	0.63
	12	15.11	. beta. -Bisabolene	495-61-4	0.47
	**13**	**15.91**	**(-)-Germacrene D**	**23986-74-5**	**4.68**
	**14**	**16.17**	**. gamma. -Muurolene**	**30021-74-0**	**29.33**
	15	16.26	. alpha. -Muurolene	10208-80-7	2.45
	16	16.37	. alpha. -Farnesene isomer [3,7,11-Trimethyl-1,3,6,10-dodecatetraene]	502-61-4	2.46
	**17**	**16.64**	**gamma-Cadinene**	**39029-41-9**	**4.32**
	**18**	**16.88**	**. delta. -Cadinene**	**483-76-1**	**4.15**
	19	17.05	Naphthalene, 1,2,3,4,4a,7-hexahydro-1,6-dimethyl-4-(1-methylethyl)-	16728-99-7	0.42

	20	17.17	alpha-cadinene	24406-05-1	1.36
	21	17.73	1,6,10-Dodecatrien-3-ol, 3,7,11-trimethyl-, [S-(Z)]-	7212-44-4	2.33
	22	18.14	Cubebol	23445-02-5	1.35
	23	18.39	(1R,7S, E)-7-Isopropyl-4,10-dimethylenecyclodec-5-enol	81968-62-9	0.54
	24	19.02	(1S,2E,6E,10R)-3,7,11,11-Tetramethylbicyclo [8.1.0] undeca-2,6-diene	24703-35-3	0.31
	25	19.77	. tau. -Cadinol	5937-11-1	1.21
	26	20.11	. tau. -Muurolol	19912-62-0	1.52
	27	20.83	. gamma. -Cadinene	30021-74-0	1.86
	28	21.45	Tridecanal	10486-19-8	0.31
	**29**	**23.00**	**Benzoic acid- phenylmethyl ester**	**120-51-4**	**13.24**
	30	24.00	Hexadecanal	629-80-1	0.37
	31	25.58	Cyclohexadecane P489	295-65-8	0.39
	32	28.15	Trachylobane	5282-35-9	0.32
E. kebericho	1	5.26	1,3-Cyclohexadiene, 2-methyl-5-(1-methylethyl)-	99-83-2	0.70
	**2**	**5.40**	**delta-3-Carene**	**13466-78-9**	**4.56**
	**3**	**6.08**	**2(10)-Pinene**	**127-91-3**	**4.20**
	4	6.21	1,6-Octadiene, 7-methyl-3-methylene-	123-35-3	0.18
	5	6.77	Benzene, 1-methyl-4-(1-methylethyl)-	25155-15-1	0.15
	6	6.86	2,6-Octadien-1-ol, 3,7-dimethyl-, acetate, (E)-	141-12-8	2.01
	7	7.30	1,4-Cyclohexadiene, 1-methyl-4-(1-methylethyl)-	99-85-4	0.55
	8	7.77	4-Isopropylidene-1-cyclohexene	586-62-9	0.19
	9	7.91	Bicyclo[2.2.1]heptane, 2,2-dimethyl-3-methylene-	79-92-5	0.22
	10	8.70	Verbenol P294	1845-30-3	0.59
	11	9.04	p-Mentha-1,5-dien-8-ol	1686-20-0	0.41
	12	9.24	3-Cyclohexen-1-ol, 4-methyl-1-(1-methylethyl)-	562-74-3	0.39
	13	11.28	1,7,7-Trimethylbicyclo [2.2.1] hept-2-yl acetate	5655-61-8	1.95
	14	12.14	Silphiperfol-5-ene	138752-24-6	0.85
	15	12.34	Longifolene-(V4)	61262-67-7	3.52
	16	12.61	Silphinene	74284-57-4	0.17
	17	13.06	(+)-Cycloisosativene	22469-52-9	2.08
	**18**	**13.31**	**Pethylbrene**	**442900-37-0**	**8.89**
	**19**	**13.47**	**Modephene**	**68269-87-4**	**8.59**
	20	13.63	(1R,3aS,5aS,8aR)-1,3a,4,5a-Tetramethyl-1,2,3,3a,5a,6,7,8-octahydrocyclopenta[c]pentalene	65372-78-3	0.54

	**21**	**13.79**	**Benzene, 1,2-dimethoxy-4-(2-propenyl)-**	**93-15-2**	**6.12**
	22	14.10	(1R,3aS,5aS,8aR)-1,3a,5a-Trimethyl-4-methylenedecahydrocyclopenta[c]pentalene	71596-72-0	2.84
	23	14.33	1,1,7-Trimethyl-4-methylenedecahydro-1H-cyclopropa[e]azulene	489-39-4	0.17
	24	14.80	(1R,3aS,8aS)-7-Isopropyl-1,4-dimethyl-1,2,3,3a,6,8a-hexahydroazulene	36577-33-0	0.14
	25	15.00	(-)-1,2,2. alpha.,3,3,4,6,7,8,8. alpha. -decahydro-2. alpha.,7,8-trimethylacenaphthylene	997220-90-9	1.50
	26	15.11	1,4,7, -Cycloundecatriene, 1,5,9,9-tetramethyl-, Z, Z, Z-	997220-60-2	0.86
	27	15.59	. alpha. -Gurjunene	489-40-7	0.62
	28	15.85	trans-. beta. -Ionone	79-77-6	2.93
	29	15.93	. beta. -Selinene	17066-67-0	2.12
	30	16.13	2-Isopropenyl-4a,8-dimethyl-1,2,3,4,4a,5,6,8a-octahydronaphthalene	207297-57-2	0.72
	**31**	**16.41**	**Naphthalene, 1,2,3,5,6,8a-hexahydro-4,7-dimethyl-1-(1-methylethyl)-, (1S-cis)-**	**483-76-1**	**9.80**
	**32**	**16.68**	**(3S,6S)-6-Isopropyl-3-methyl-2-(propan-2-ylidene)-3-vinylcyclohexanone**	**21698-46-4**	**3.69**
	33	16.79	. delta. -Cadinene	483-76-1	0.57
	34	18.14	1.beta.,4. beta.H,10. beta.H-Guaia-5,11-diene	22567-17-5	2.47
	35	18.38	Caryophyllene oxide	1139-30-6	0.20
	36	18.60	. gamma. -Gurjunenepoxide-(2)	184705-51-9	0.77
	37	18.69	. alpha. -dimethyl-. alpha. -ionone	0-00-0	0.27
	38	18.86	1H-Benzocyclohepten-7-ol, 2,3,4,4a,5,6,7,8-octahydro-1,1,4a,7-tetramethyl-, cis-	6892-80-4	0.84
	39	19.06	1.beta.,4. beta.H,10. beta.H-Guaia-5,11-diene	22567-17-5	1.51
	40	19.76	. tau. -Cadinol	5937-11-1	0.30
	41	20.00	2,6,10,10-Tetramethylbicyclo [7.2.0] undeca-1,6-diene	87-44-5	2.24
	42	20.12	. alpha. -Cadinol	481-34-5	1.87
	43	20.29	2-((2R,4aR,8aS)-4a-Methyl-8-methylenedecahydronaphthalen-2-yl) prop-2-en-1-ol	515-20-8	0.86
	44	20.86	7-Isopropenyl-1,4a-dimethyl-4,4a,5,6,7,8-hexahydro-3H-naphthalen-2-one	473-08-5	0.44
	45	20.95	alpha-Costic aldehyde	4586-01-0	0.25
	46	21.44	Humulenol-II	19888-00-7	0.19
	447	24.09	(1S,7S,8aR)-1,8a-Dimethyl-7-(prop-1-en-2-yl)-1,2,3,7,8,8a-hexahydronaphthalene	190327-38-9	0.43
	48	25.48	2-((2R,4aR,8aS)-4a-Methyl-8-methylenedecahydronaphthalen-2-yl) prop-2-en-1-ol	515-20-8	0.43
	49	27.13	Dihydrodehydrocostus lactone	4955-03-7	0.51
	**50**	**28.66**	**Dehydrocostus lactone**	**477-43-0**	**13.74**

Repellency of essential oils against *Anopheles arabiensis*

In dose-response experiment, the five essential oils at a rate of 1.67μL/cm2 demonstrated repellent effects against *An. arabiensis bites*, as shown in Table 3. The essential oils obtained from both leaves and seeds of *C. macrostachyus* had the lowest ED_{50} (0.45%; 95%CI=0.39-0.51) and ED_{99} (2.48%; 95%CI=2.29-2.73) values, as well as ED_{50} (0.48%; 95%CI=0.41-0.53) and ED_{99} (2.50%; 95%CI=2.31-2.77). The oil from *J. procera* had the lowest repellent activity, with ED_{50} (0. 97%; 95% CI=0.92-1.06) and ED_{99} (4.83%; 95% CI=4.55-5.16).

Table 3. The effective dose of essential oils corresponding to 50% (ED_{50}) and 99% (ED_{99}) protection from mosquito landing against laboratory reared *Anopheles arabiensis*, 2025

Essential oils	ED_{50} (95%CI)	ED_{99} (95%CI)	X^2 (df=3)	p-value
C. macrostachyus (leaves)	0.45 (0.39-0.51)	2.48 (2.29-2.73)	6.04	0.109
C. macrostachyus (seeds)	0.48 (0.41-0.53)	2.50 (2.31-2.77)	5.94	0.101
E. kebericho	0.96 (0.94-1.02)	4.10 (3.89-4.36)	7.46	0.059
E. globulus	0.83 (0.79-0.87)	3.87 (3.64-4.14)	3.67	0.299
J. procera	0. 97 (0.92-1.06)	4.83 (4.55-5.16)	7.25	0.064

Repellency duration of the essential oils

Essential oils isolated from *C. macrostachyus* leaves and seeds provided protection for up to 180 minutes, with a percentage repellency of 87-100%. After 210 minutes, the protection decreased to 47–53%, and after 270 minutes, it reached 0 at 20% concentration. While *J. procera* essential oil provided the shortest protection, providing 93-100% protection after 30 minutes post-application and decreasing to 70-80% by 60 minutes. DEET, a standard commercial repellent,

Formatted: Font: 16 pt

Formatted: Font: Bold

Formatted: Font: Bold

Formatted: Font: 16 pt

gave significantly greater repellency, up to 93-100% for as long as 330 minutes of the test duration against *An. arabiensis* (Fig 1).

All the oils showed repellent activity against *An. arabiensis* providing 80-100% protection for 30 minutes at 10% concentration (Fig 2). Both oils from *C. macrostachyus* seeds/leaves protected for up to 150 minutes with a percentage repellency of 86-93%, and *E. kebericho* for 60 minutes with 86-93% percentage repellency, while *J. procera* oil gave the weakest protection, with 53% and lower protection for 60 minutes post application. DEET, the standard commercial repellent, provided substantially longer repellency (84-100% for up to 330 minutes post-application).

~~Figure~~ Fig. 1. Percentage repellency provided by the essential oils at 20% test concentration and the standard DEET against laboratory reared *Anopheles arabiensis*, 2025

~~Figure~~ Fig. 2. Percentage repellency provided the essential oils at 10% concentration and the standard DEET against laboratory reared *Anopheles arabiensis*, 2025

Comparisons of the duration of repellency among the five oils and DEET using a non-parametric Kruskal-Wallis H test indicated a significant difference (P < 0.001) at both 10% and 20% concentrations. A pairwise analysis of the five oils using Dunn's post hoc test, after a non-parametric Kruskal-Wallis H test, significant differences revealed that oils of both the leaves and seeds of *C. macrostachyus* as well as *E. kebericho* have a significantly greater length of protection time compared to the oils of *E. globulus* and *J. procera*. However, there was no significant difference between them. ~~Mean~~ Median time of protection in minutes followed by the same letter ~~shows~~ showed no significantly different value in Dunn's post hoc test (P > 0.05) (Table 4).

Formatted: Font: Bold

Formatted: Font: Bold

Formatted: Font: Bold

Formatted: Font: Bold

Table 4. Median time of protection of the oils and a DEET against laboratory reared *Anopheles arabiensis*, 2025

Essential oil formulations	Median protection (minutes) at 10%.	Range (min-max)	Median protection (minutes) at 20%.	Range (min-max)
DEET (Positive Control)	390^{a}	270 – 390	362.00^{a}	330 – 390
C. macrostachyus (leaves)	152^{b}	150 – 153	211^{b}	153 – 241
C. macrostachyus (seeds)	141^{b}	93 – 153	214^{b}	151 – 270
E. kebericho	92^{b}	62 –151	151^{b}	91 – 153
E. globulus	62^{c}	31 – 91	91^{c}	33 – 152
J. procera	33^{c}	31 – 63	61^{c}	32 – 91

Duration of repellent effects from blended essential oils

All the combination of essential oils produced significant protection soon after the application. However, combination of *C. macrostachyus*+*E. kebericho* and *J. procera*+*C. macrostachyus* gave the longest mean duration of protection lasting 90 minutes with greater than 80% protection against *An. arabiensis* (Fig 3).

According to statistical comparison using a non-parametric Kruskal-Wallis H test, there was a significant difference ($P < 0.001$) in the median time of repellency between the binary combinations of oils and a DEET. In pairwise comparisons using Dunn's post hoc test, following a non-parametric Kruskal-Wallis H test, significant differences two of the six blended oils *C. macrostachyus* (seeds) + *E. kebericho* and *J. procera* + *C. macrostachyus* (seeds) showed significantly longer median times of protection. Median time of protection in minutes followed by the same letter shows showed no significantly different value in Dunn's post hoc test ($P > 0.05$) (Table 5).

The comparisons of protection time between the single and blended oils using a non-parametric Kruskal-Wallis H test showed that there are significant differences between them ($P < 0.001$). The result of pairwise comparisons using Dunn's post hoc test, following a non-parametric Kruskal-Wallis H test, significant differences indicated that blending with *C. macrostachyus* (seeds) or *E. kebericho* significantly improves repellent efficacy compared to *J. procera* alone ($P < 0.001$), but other combinations preserve the repellent efficacy of the stronger component ($P > 0.05$). Median time of protection in minutes followed by the same letter showed no significantly different value in Dunn's post hoc test ($P > 0.05$) (Table 6).

~~Figure~~ **Fig. 3. Percentage repellency provided by the blended essential oils at 20% concentration and a DEET against laboratory reared *Anopheles arabiensis*, 2025**

Table 5. Mean time of protection of the blended oils and DEET at 20% against laboratory reared *Anopheles arabiensis*, 2025

Blended essential oil formulations	Median protection (minutes)	Range (min-max)
DEET	390[a]	330 – 390
C. macrostachyus (seeds) + *E. kebericho*	152[b]	90 – 211
J. procera + *C. macrostachyus* (seeds)	150[b]	61 – 153
E. globulus + *C. macrostachyus* (seeds)	93[c]	33 – 212
E. globulus + *E. kebericho*	93[c]	61 – 150
J. procera + *E. kebericho*	91[c]	63 – 93
E. globulus + *J. procera*	91[c]	32 – 150

Formatted: Font: Bold

Formatted: Font: Bold

Formatted: Font: Bold

Formatted: Font: Bold

Table 6. Comparisons of protection time between the single and blended oils at 20% against laboratory reared *Anopheles arabiensis*, 2025

Single and blended oil formulations	Median protection (minutes)	Range (min-max)
C. macrostachyus (seeds)	214[a]	151 – 270
E. kebericho	151[a]	91 – 153
E. globulus	91[ab]	33 – 152
J. procera	61[b]	32 – 91
C. macrostachyus (seeds) + *E. kebericho*	152[a]	90 – 211
J. procera + *C. macrostachyus* (seeds)	150[a]	61 – 153
E. globulus + *C. macrostachyus* (seeds)	93[a]	33 – 212
E. globulus + *E. kebericho*	93[a]	61 – 150
J. procera + *E. kebericho*	91[a]	63 – 93
E. globulus + *J. procera*	91[ab]	32 – 150

Discussion

The present study evaluated the essential oils extracted from parts of selected plants such as ~~*Croton*~~ *C. macrostachyus*, ~~*Echinops*~~ *E. kebericho*, ~~*Eucalyptus*~~ *E. globulus*, and ~~*Juniperus*~~ *J. procera* in the Ghibe valley for their repellency against *An. arabiensis* under laboratory condition. In this study, a high amount of essential oil was extracted from the leaves of ~~*Eucalyptus*~~ *E. globulus.* Essential oil yields of fresh *E. globulus* in Ethiopia and elsewhere range from over 0.2% to 5% with varying degrees depending on location, season, and age, with younger leaves generally yielding more oil in line~~d~~ with the result of the current study (43). The lowest oil was obtained from leaves of *C. macrostachyus* among the four plants, similar to the

Formatted: Font: Bold

Formatted: Font: Bold

Formatted: Font: 18 pt

results of previous study (44). Differences in the essential oil yields could be attributed to genetic factors, collection place, plant developmental stage, season of collection, and extraction methods (45, 46).

A total of 162 compounds were identified from the four essential oils assessed for their repellent properties in this study, using GC-MS-analysis. Of these, the most abundant compounds previously known to have repellent properties such as 1,8-Cineole, delta-3-Carene and α-Terpinyl acetate, 13-epi-manoyl oxide, -gamma-Muurolene, benzoic acid-phenylmethyl ester, and Dehydrocostus lactone were identified by the GC-MS-analysis (47, 48). The predominant compounds such as -gamma-muurolene and benzoic acid-phenylmethyl ester, which are found in the *C. macrostachyus* seed oil, and dehydrocostus lactone in the *E. kebericho* root oil showed a positive correlation trend with the repellent efficacy of the oils in varying strength. These findings suggest that achieving an optimal balance of these compounds is crucial for maximizing mosquito-repellent effects. All of them have been known to have insect-repellent properties, including mosquitoes, in the literature that supported the findings of the current study (47, 49, 50).

Other less abundant compounds identified from all the essential oils, like linalool and pinene which, previously reported to have repellent properties (51). Beta- phellandrene that had previously reported as mosquito repellent properties was identified from *J. procera* oil (52). Furthermore, in lined with the previous study, all of the essential oils examined in this study are mainly composed of monoterpenes, sesquiterpenes, alcohols, and phenols that can be neurotoxic to mosquitoes (53).

The current investigation found that essential oils of the selected four plants, namely *C. macrostachyus*, *E. kebericho*, *E. globulus* and *J. procera*, have significant repellent properties

with varying levels of effects against *An. arabiensis* bites. As a result, the findings of this study supported the findings of the earlier ethnobotanical study on the usage of these plants for the traditional control of mosquitoes and other arthropods in the Ghibe valley, southwest Ethiopia (30). The present study revealed that essential oil from the leaf and seed parts of *C. macrostachyus* showed promising repellent activity. A comparison of the five essential oils' ED_{50} values (dosage giving a 50% repellent effect) against *An. arabiensis* revealed that the repellent properties varied with dose. Among the five essential oils, the two extracted from the leaves and seeds of *C. macrostachyus* showed nearly the same repellent activities compared to each other. The ED_{50} of both oils of *C. macrostachyus* ~~were~~ was lower compared to the five essential oils. ~~This~~ These results demonstrated that oils from both the leaf and seed parts of *C. macrostachyus* had the strongest repelling efficacy. This may be attributed to the variations in the type and quantity of active compounds present in the oil, which could influence repelling properties (54). The scientific literature on the repellent efficacy of essential oils against *An. arabiensis* is often inconsistent (23). These deviations often appear to directly correlate with the concentration of the essential oil tested~~,~~ and the type of solvent used to dilute the essential oil (55). ~~Also~~Furthermore, the chemical consistency of essential oils can be variable. Factors, such as the location, environmental conditions, ~~and~~ specific plant species, mosquito species, and test subjects, may account for some of the inconsistencies (56). Variation in the results of such type of repellency studies, including the current investigation, could come from differences in chemical profile, dosages, mosquito species, formulations, extraction, and test methods (52). In some prior laboratory and field trials, the protection time was considered until mosquitoes landed, whereas in other studies, it was the time until mosquitoes bite (23, 57).

Among the five essential oils, both the leaf and seed essential oils of *C. macrostachyus* protected for more than 150 minutes at a 10% concentration and more than 180 minutes at a 20% concentration. The increased protection period of the oil at greater concentrations could be attributed to an increase in the concentration of the active ingredient contained in the oils. This demonstrates that increasing the concentration of essential oils can increase repellency, which is supported by the findings of several previous studies (33, 58). The duration of protection exhibited by the essential oil of *C. macrostachyus* in the present study is longer than *O. suave, O. americanum, O. kilimandscharicum*, and *Lantana camara* against *An. gambiae*, which have shown less than an hour of complete protection in laboratory studies in Kenya (59). However, the use of essential oils as insect repellents is highly limited by their fast volatility and oxidation rate (60). The Environmental Protection Agency (EPA) of the United States has established that 120 minutes should be the standard minimum time required for a repelling effect of greater than 80% protection in order to qualify for registration (61). Hence, in line with this, the essential oils of both the leaves and seeds of *C. macrostachyus* provide promising protection against laboratory-reared *An. arabiensis* that meet the criteria for registration. However, field testing of these oils as much as possible in real-world conditions is required for registration. The evaluation of essential oil of *C. macrostachyus* for its repellency against *An. arabiensis* is the first in Ethiopia in spite of its wide use as herbal medicine by the indigenous people of tropical Africa (62). ~~Recent~~ A recent laboratory study in the country reported that the methanol crude extract and ethyl acetate fractions of *C. macrostachyus* leaves showed a promising larvicidal and adulticidal activity against *An. gambiae* (63).

Essential oil of *E. kebericho* protected the skin for more than 90 minutes at 20% concentrations. These findings support the traditional usage of *E. kebericho* as a mosquito

repellent in the Ghibe valley, Ethiopia (30). A previous study found that the essential oil of *E. kebericho* has strong repellent properties against non-blood-fed female *Anopheles arabiensis* at quite low concentrations. However, the duration of protection period was not specified (Jemberie et al., 2016). The essential oil derived from *E. kebericho* has a longer duration of protection than oils of *O. suave, O. americanum, O. kilimandscharicum*, and *Lantana camara* against *An. gambiae* (59).

E. globulus and *J. procera* protected the skin for less than an hour in both 10% and 20% concentrations, with *E. globulus* protected for more than an hour at 20% concentration. Comparable result was reported by previous studies on essential oil of *E. globulus* in Ethiopia (64). The complete protection time of *E. globulus* and *J. procera* was comparable *to O. suave, O. americanum, O. kilimandscharicum* and *Lantana camara An. gambiae* (59). ~~In contrary to the present findings, previous~~ Previous similar study conducted in Ethiopia using the methanol extract of *J. procera* showed remarkable protection against *An. arabiensis* (33). It has also been ~~also~~ reported that the degree of repellency could be improved significantly when the methanol extract of *J. procera* concentration is increased (33).

When comparing the repellent effects of the essential oil blends, the 1:1 binary combination of *C. macrostachyus* with *E. kebericho*, *J. procera* with *C. macrostachyus* (seeds) demonstrated a greater effectiveness than the remaining oil combinations. As a result, when two essential oils were combined, the kind, quantity, or ratio of active constituents changed (65). This may lengthen or shorten the effective protection period against mosquito bites. When one essential oil is mixed with another, it may cause a synergistic or antagonistic reaction that change repelling properties. The result of the current study showed that blending with *C. macrostachyus* (seeds) or *E. kebericho* significantly improves repellent efficacy or possesses a strong synergistic effect

compared to *J. procera* alone. This strong synergistic effect might be due to the heavy sesquiterpene lactones and esters found in *E. kebericho* and *C. macrostachyus* act as natural fixatives (66). But other combinations preserve the repellent efficacy of the stronger component. These findings suggest that the majority of blended essential oils exhibited comparable repellency to single essential oils with slight differences. Therefore, the result implies that a 1:1 combination of most of the essential oils tested in this study did not cause a synergistic or antagonistic reaction. This lack of chemical interaction implies that the volatile compounds either target the same mosquito receptors or evaporate at their own individual, unchanged rates, resulting in no longer complete protection time (CPT) than single oils [63].

The shorter protection duration of the essential oils compared to DEET in the current study may suggest that there is faster loss of repellent activity due to faster volatilization of compounds of the essential oils (67). To maximize the effectiveness of essential oils as repellent against mosquito bites, particular formulations might require. Adding 5% vanillin to *E. globulus* oil increased its protection period against *Ae. albopictus* from 3-5 hours under laboratory conditions in China (68). Citronella oil has long been used in commercial repellent formulations and is popular in India, however it is generally rated as less effective than repellents using synthetic active ingredients (69). Despite the fact that all of the essential oils employed in this study are less effective than DEET, they are thought to be safer and can be used frequently.

Limitations of the study

Formatted: Font: 16 pt

The current study was limited by no bioassay was conducted with the major compounds of the essential oils to identify potent repellent chemicals against mosquitoes. Due to the small sample size, the correlation analysis investigated in this study may not accurately represent the relationships between the identified chemical compounds and mosquito-repellent effect,

necessitating further research with a larger sample size. The study also did not evaluate mixtures of three or more oils, which may have limited an in-depth understanding from analyzing these aspects. Furthermore, the data did not include a safety assessment of the essential oils.

Conclusions

All of the five essential oils examined in this study had significant repellent properties against laboratory-reared *An. arabiensis* bites, but with different degrees of efficacy and shorter protection times than DEET. ~~*Croton*~~ *C. macrostachyus* leaf and seed oils provided significantly longer protection against laboratory-reared *An. arabiensis* bites, lasting 180 minutes and 150 minutes at 20% and 10% concentrations, respectively. This meets the Environmental Protection Agency's registration criteria; thus, it should be studied further under field settings. Although plant essential oils are less effective than DEET, they are thought to be safer and can be used frequently. Evaluation of skin toxicity and the strategies for increasing the efficacy of repellents, such as purification of the active ingredients, reduction in skin absorption, gradual release of the repellents, and increase in repellency persistence, must be also explored by future study.

Acknowledgments

The authors would like to acknowledge the Aklilu Lemma Institute of Health Research, Addis Ababa University, Ethiopia, for supporting us with field and laboratory materials. We are most grateful to the Center for Innovative Drug Development and Therapeutic Trials for Africa (CDT-Africa) for giving us permission and support for essential oil extractions. We thank Mr. Wosen Sisay and Mr. Jiregna Gemechu for their assistance during repellency test. We would like to thank Mr. Kebede Shenkute for his support in the identification of chemical compounds of essential oils at natural product chemistry laboratory, Adama Science and Technology University.

Formatted: Font: 18 pt

Formatted: Font: 18 pt

List of abbreviations

Formatted: Font: 18 pt

ALIPB	Aklilu Lemma Institute of Pathobiology
CDT-Africa	Center for Innovative Drug Development and Therapeutic Trials for Africa
SPSS	Statistical Package for the Social Sciences

Author Contributions

Conceptualization: Zeyede Teshome, Abebe Animut, Mirutse Giday, Esayas Aklilu.

Data curation: Zeyede Teshome.

Formal analysis: Zeyede Teshome.

Funding acquisition: Zeyede Teshome, Abebe Animut, Mirutse Giday, Esayas Aklilu.

Investigation: Zeyede Teshome, Abebe Animut, Belete Adefris Legesse, Mirutse Giday, Esayas Aklilu.

Methodology: Zeyede Teshome, Abebe Animut, Belete Adefris Legesse, Mirutse Giday, Esayas Aklilu.

Supervision: Zeyede Teshome, Abebe Animut, Mirutse Giday, Esayas Aklilu.

Validation: Zeyede Teshome, Abebe Animut, Mirutse Giday, Esayas Aklilu.

Visualization: Zeyede Teshome, Abebe Animut, Mirutse Giday, Esayas Aklilu.

Writing – original draft: Zeyede Teshome.

Writing – review & editing: Zeyede Teshome, Abebe Animut, Belete Adefris Legesse, Mirutse Giday, Esayas Aklilu.

REFERENCES

1. Egwu CO, Aloke C, Chukwu J, Agwu A, Alum E, Tsamesidis I, et al. A world free of malaria: It is time for Africa to actively champion and take leadership of elimination and eradication strategies. African Health Sciences. 2022;22(4):627-40.
2. Butler CD, Amegah AK, Wright CY, Bowles DC. Health, Climate and Challenges in Africa: 2024–2100. Climate Change and Global Health: Primary, Secondary and Tertiary Effects: CABI GB; 2024. p. 378-94.
3. Andrade MV, Noronha K, Guedes GR, Diniz BC, Silva VA, de Souza A, et al. Health-related quality of life due to malaria: a systematic review. Quality of Life Research. 2025;34(2):325-39.
4. Chen J, Tian X, Guo D, Gu H, Duan Y, Li D. Global trends and burdens of neglected tropical diseases and malaria from 1990 to 2021: a systematic analysis of the Global Burden of Disease Study 2021. BMC Public Health. 2025;25(1):1307.
5. Kendie FA, Hailegebriel W/kiros T, Nibret Semegn E, Ferede MW. Prevalence of Malaria among Adults in Ethiopia: A Systematic Review and Meta-Analysis. Journal of tropical medicine. 2021;2021(1):8863002.
6. Organization WH. Global Malaria Programme operational strategy 2024-2030: World Health Organization; 2024.
7. Woldesenbet D, Tegegne Y, Mussema A, Tamene E, Mohamed K, Abebe W, et al. Can Ethiopia eliminate malaria? Malaria burden: insights from the pre-elimination era, current challenges and perspectives. Frontiers in Malaria. 2025;3:1492444.
8. Takken W, Charlwood D, Lindsay SW. The behaviour of adult Anopheles gambiae, sub-Saharan Africa’s principal malaria vector, and its relevance to malaria control: a review. Malaria Journal. 2024;23(1):161.

Formatted: Indent: Left: 0", Hanging: 0.5", Line spacing: Double

Formatted: Font: (Default) Times New Roman, 12 pt

9. Mwima R, Hui T-YJ, Nanteza A, Burt A, Kayondo JK. Potential persistence mechanisms of the major Anopheles gambiae species complex malaria vectors in sub-Saharan Africa: a narrative review. Malaria Journal. 2023;22(1):336.

10. Adugna F, Wale M, Nibret E. Review of Anopheles mosquito species, abundance, and distribution in Ethiopia. Journal of tropical medicine. 2021;2021(1):6726622.

11. Woyessa D, Yewhalaw D. Anopheles mosquito fauna, blood meal sources and transmission intensity from high and moderate malaria endemic areas of Ethiopia. Scientific Reports. 2025;15(1):10636.

12. Emiru T, Getachew D, Murphy M, Sedda L, Ejigu LA, Bulto MG, et al. Evidence for a role of Anopheles stephensi in the spread of drug-and diagnosis-resistant malaria in Africa. Nature medicine. 2023;29(12):3203-11.

13. Hawaria D, Amanuel T, Anbesie A, Zhong D, Kibret S, Lee M-C, et al. Elevated Plasmodium Sporozoite Infection Rates in Primary and Secondary Malaria Vectors in Anopheles stephensi-Infested Areas of Ethiopia. Insects. 2025;16(5):462.

14. Braack L, Macdonald MB. Outdoor biting by malaria vectors: what are the options for intervention? Malaria Journal. 2025.

15. Benelli G, Beier JC. Current vector control challenges in the fight against malaria. Acta tropica. 2017;174:91-6.

16. Odero JI, Abong'o B, Moshi V, Ekodir S, Harvey SA, Ochomo E, et al. Early morning anopheline mosquito biting, a potential driver of malaria transmission in Busia County, western Kenya. Malaria journal. 2024;23(1):66.

17. Owuor KO. Resting Behaviour of African Malaria Vectors in an Era of High Indoor Insecticide Use: University of Nairobi; 2022.

Formatted: Font: (Default) Times New Roman, 12 pt, Font color: Red

Formatted: Font: (Default) Times New Roman, 12 pt

18. Mukisa MC, Kassano JJ, Mwalugelo YA, Ntege C, Kahamba NF, Finda MF, et al. Analysis of the 24-h biting patterns and human exposures to malaria vectors in south-eastern Tanzania. Parasites & Vectors. 2024;17(1):445.

19. Organization WH. Global report on insecticide resistance in malaria vectors: 2010–2016. 2018.

20. Akuoko OK, Dhikrullahi SB, Hinne IA, Mohammed AR, Owusu-Asenso CM, Coleman S, et al. Biting behaviour, spatio-temporal dynamics, and the insecticide resistance status of malaria vectors in different ecological zones in Ghana. Parasites & Vectors. 2024;17(1):16.

21. Bedasso AH, Gutto AA, Waldetensai A, Eukubay A, Bokore GE, Kinde S, et al. Malaria vector feeding, peak biting time and resting place preference behaviors in line with Indoor based intervention tools and its implication: scenario from selected sentinel sites of Ethiopia. Heliyon. 2022;8(12).

22. Sougoufara S, Ottih EC, Tripet F. The need for new vector control approaches targeting outdoor biting Anopheline malaria vector communities. Parasites & Vectors. 2020;13(1):295.

23. Asadollahi A, Khoobdel M, Zahraei-Ramazani A, Azarmi S, Mosawi SH. Effectiveness of plant-based repellents against different Anopheles species: a systematic review. Malaria journal. 2019;18(1):436.

24. Xue R-D, Zhao T-Y. Concepts of Best Management Practices for Intergrated Pest, Mosquito, and Vector Management. Bio-mathematics, Statistics, and Nano-Technologies: Chapman and Hall/CRC; 2023. p. 13-20.

25. Jiao X-Y, Wang C-S, Pan D, Liu P, Wang X-R, Zhang C, et al. Unveiling the potential health risks induced by photolysis of insect repellents DEET under simulated sunlight. Water Cycle. 2025;6:206-12.

26. Hazarika H, Krishnatreyya H. Technological Advancements in Mosquito Repellents: Challenges and Opportunities in Plant-Based Repellents. Acta Parasitologica. 2025;70(3):117.

27. Roy DN, Goswami R, Pal A. The insect repellents: A silent environmental chemical toxicant to the health. Environmental toxicology and pharmacology. 2017;50:91-102.

28. Yadav DK, Rathee S, Sharma V, Patil UK. A comprehensive review on insect repellent agents: medicinal plants and synthetic compounds. Anti-Inflammatory & Anti-Allergy Agents in Medicinal Chemistry. 2024.

29. Adimas ZT, Adimas MA, Abera BD. Plant-based bioactive compounds for grain storage: a comprehensive review. Cogent Food & Agriculture. 2024;10(1):2316152.

30. Teshome Z, Teka A, Animut A, Arage M, Aklilu E, Giday M. Ethnobotanical study of plants used for traditional control of mosquitoes and other arthropod pests in the Ghibe valley, southwest Ethiopia. Tropical Medicine and Health. 2025;53(1):1-27.

31. Pushpalatha E, Kocher DK, Prasad A, Bhowmik I, Anirudh E, Anoopkumar A, et al. Mosquito Control: Part II: Plant-Based Products or Phytochemicals. Mosquitoes of India: CRC Press; 2025. p. 170-83.

32. Ríos J-L. Essential oils: What they are and how the terms are used and defined. Essential oils in food preservation, flavor and safety: Elsevier; 2016. p. 3-10.

Formatted: Font: (Default) Times New Roman, 12 pt, Font color: Red

Formatted: Font: (Default) Times New Roman, 12 pt

33. Karunamoorthi K, Girmay A, Hayleeyesus SF. Mosquito repellent activity of essential oil of Ethiopian ethnomedicinal plant against Afro-tropical malarial vector Anopheles arabiensis. Journal of King Saud University-Science. 2014;26(4):305-10.

34. Fikru S, Tolossa K, Lindemann P, Bucar F, Asres K. Larvicidal, ovicidal, and repellent activities of Leucas stachydiformis (Hochst. ex Benth.) Briq essential oil against Anopheles arabiensis. Journal of Tropical Medicine. 2024;2024(1):1051086.

35. Berhe M, Dugassa S, Shimelis S, Tekie H. Repellence and larvicidal effects of some selected plant extracts against adult Anopheles arabiensis and Aedes aegypti larvae under laboratory conditions. International Journal of Tropical Insect Science. 2021:1-8.

36. Adem AA, Belete A, Raorane ML, Junker BH, Neubert RH, Gebre-Mariam T. Formulation and evaluation of nanoemulgel loaded with essential oils with mosquito repellent activities. Journal of Drug Delivery Science and Technology. 2024;100:105991.

37. Fiehn O, Kopka J, Trethewey RN, Willmitzer L. Identification of uncommon plant metabolites based on calculation of elemental compositions using gas chromatography and quadrupole mass spectrometry. Analytical chemistry. 2000;72(15):3573-80.

38. Organization WH. Guidelines for efficacy testing of mosquito repellents for human skin. World Health Organization; 2009.

39. Lopez AD, Whyms S, Luker HA, Galvan CJ, Holguin FO, Hansen IA. Repellency of essential oils and plant-derived compounds against Aedes aegypti mosquitoes. Insects. 2025;16(1):51.

40. Luker HA, Salas KR, Esmaeili D, Holguin FO, Bendzus-Mendoza H, Hansen IA. Repellent efficacy of 20 essential oils on Aedes aegypti mosquitoes and Ixodes scapularis ticks in contact-repellency assays. Scientific reports. 2023;13(1):1705.

Formatted: Font: (Default) Times New Roman, 12 pt, Font color: Red

41. Trongtokit Y, Rongsriyam Y, Komalamisra N, Apiwathnasorn C. Comparative repellency of 38 essential oils against mosquito bites. Phytotherapy Research: An International Journal Devoted to Pharmacological and Toxicological Evaluation of Natural Product Derivatives. 2005;19(4):303-9.

42. Yap H, Jahangir K, Chong A, Adanan C, Chong N, Malik Y, et al. Field efficacy of a new repellent, KBR 3023, against Aedes albopictus (SKUSE) and Culex quinquefasciatus (SAY) in a tropical environment. Journal of vector ecology: journal of the Society for Vector Ecology. 1998;23(1):62-8.

43. Nigussie G, Werede Y. Chemical profile and antimicrobial activity of essential oil of Eucalyptus globulus leaves from Haramaya campus and Entoto park, Ethiopia. Chemistry J. 2020;6:187-98.

44. Tariku Y, Hymete A, Hailu A, Rohloff J. Constituents, antileishmanial activity and toxicity profile of volatile oil from berries of Croton macrostachyus. Natural product communications. 2010;5(6):1934578X1000500635.

45. Mehalaine S, Chenchouni H. Quantifying how climatic factors influence essential oil yield in wild-growing plants. Arabian Journal of Geosciences. 2021;14(13):1257.

46. Dhotre I. A comprehensive review on progression and innovations in microwave assisted extraction technology for essential oils. Journal of Chemical Technology & Biotechnology. 2025;100(5):894-907.

47. Abdo BM, Asfaw BT, Choudhary MI. Bioassay-guided isolation of dehydrocostus lactone from Echinops kebericho as potential biopesticide against Aedes aegypti. Natural Product Research. 2025:1-7.

Formatted: Font: (Default) Times New Roman, 12 pt

Formatted: Font: (Default) Times New Roman, 12 pt, Font color: Red

48. Klocke JA, Darlington MV, Balandrin MF. 1, 8-Cineole (Eucalyptol), a mosquito feeding and ovipositional repellent from volatile oil of Hemizonia fitchii (Asteraceae). Journal of chemical ecology. 1987;13(12):2131-41.

49. Feng Y, Chen J, Zhang A. Commercially available natural benzyl esters and their synthetic analogs exhibit different toxicities against insect pests. Scientific Reports. 2018;8(1):7902.

50. Kamaraj C, Satish Kumar RC, Al-Ghanim KA, Nicoletti M, Sathiyamoorthy V, Sarvesh S, et al. Novel essential oils blend as a repellent and toxic agent against disease-transmitting mosquitoes. Toxics. 2023;11(6):517.

51. Tkachenko K, Varfolomeeva E. Prospects for the Use of Essential Oils as Repellants and/or Insecticides. Tropical Journal of Natural Product Research. 2022;6(6).

52. Jaenson TG, Pålsson K, Borg-Karlson A-K. Evaluation of extracts and oils of mosquito (Diptera: Culicidae) repellent plants from Sweden and Guinea-Bissau. Journal of medical entomology. 2006;43(1):113-9.

53. Wojtunik-Kulesza KA. Toxicity of selected monoterpenes and essential oils rich in these compounds. Molecules. 2022;27(5):1716.

54. Mapossa AB, Focke WW, Tewo RK, Androsch R, Kruger T. Mosquito-repellent controlled-release formulations for fighting infectious diseases. Malaria journal. 2021;20(1):165.

55. Krzyżowski M, Baran B, Łozowski B, Francikowski J. The role of dilution mediums in studies of fumigant insecticidal activity of essential oils. Journal of Pest Science. 2020;93(4):1119-24.

Formatted: Font: (Default) Times New Roman, 12 pt

56. Mugao L. Factors influencing yield, chemical composition and efficacy of essential oils. Int J Multidiscip Res Growth Eval. 2024;5(4):169-78.

57. Soonwera M, Phasomkusolsil S. Efficacy of Thai herbal essential oils as green repellent against mosquito vectors. Acta tropica. 2015;142:127-30.

58. Islam M, Hasan MM, Xiong W, Zhang S, Lei C. Fumigant and repellent activities of essential oil from Coriandrum sativum (L.)(Apiaceae) against red flour beetle Tribolium castaneum (Herbst)(Coleoptera: Tenebrionidae). Journal of Pest Science. 2009;82(2):171-7.

59. Seyoum A, Killeen GF, Kabiru EW, Knols BG, Hassanali A. Field efficacy of thermally expelled or live potted repellent plants against African malaria vectors in western Kenya. Tropical Medicine & International Health. 2003;8(11):1005-11.

60. Nerio LS, Olivero-Verbel J, Stashenko E. Repellent activity of essential oils: a review. Bioresource technology. 2010;101(1):372-8.

61. Novak RJ, Gerberg EJ. Natural-based repellent products: efficacy for military and general public uses. Journal of the American Mosquito Control Association. 2005;21(sp1):7-11.

62. Maroyi A. Ethnopharmacological uses, phytochemistry, and pharmacological properties of Croton macrostachyus Hochst. Ex Delile: a comprehensive review. Evidence-Based Complementary and Alternative Medicine. 2017;2017(1):1694671.

63. Tadesse S, Abay SM, Makonnen E, Ejigu A, Asemamaw Y, Haileselassie W, et al. Larvicidal and adulticidal effects of Ethiopian medicinal plants against Anopheles gambiae (Diptera: Culicidae). Malaria Journal. 2025;24(1):246.

64. Wano M. Evaluation of Essential oils of some local plants for their repellency against Anopheles arabiensis and Aedes aegypti. Addis Ababa University. 2006.

Formatted: Font: (Default) Times New Roman, 12 pt, Font color: Red

Formatted: Font: (Default) Times New Roman, 12 pt

65. Hategekimana A, Erler F. Comparative repellent activity of single, binary and ternary combinations of plant essential oils and their major components against Sitophilus oryzae L.(Coleoptera: Curculionidae). Journal of Plant Diseases and Protection. 2020;127(6):873-81~~.~~

66. Lee MY. Essential oils as repellents against arthropods. BioMed research international. 2018;2018(1):686027~~1.~~

~~67~~. Farina P, Conti B. Liabilities of essential oils as insect repellents. Current Opinion in Environmental Science & Health. 2024;40:100564.

Wu W, Yang Y, Feng Y, Ren X, Li Y, Li W, et al. Study of the repellent activity of 60 essential oils and their main constituents against Aedes albopictus, and nano-formulation development. Insects. 2022;13(12):1077.

Sharma R, Rao R, Kumar S, Mahant S, Khatkar S. Therapeutic potential of citronella essential oil: a review. Current Drug Discovery Technologies. 2019;16(4):330-9.

Supporting files

A representative total ion chromatogram (TIC) displaying the chemical profile of essential oils extracted by hydrodistillation. Major peaks are labeled based on the compounds listed in Table 2.

S1 file. GC-MS Chromatogram of essential oil of *Eucalyptus globulus*

S2 file. GC-MS Chromatogram of essential oil of *Juniperus procera*

S3 file. GC-MS Chromatogram of essential oil of *Croton macrostachyus*

S4 file. GC-MS Chromatogram of essential oil of *Echinops kebericho*

Formatted: Font: (Default) Times New Roman, 12 pt, Font color: Red

Formatted: Font: (Default) Times New Roman, 12 pt

Formatted: Font: (Default) Times New Roman, 12 pt, Font color: Red

Formatted: Font: (Default) Times New Roman, 12 pt, Font color: Red

Formatted: Line spacing: Double

Formatted: Font: (Default) Times New Roman, 12 pt

Formatted: Indent: Hanging: 0.5", Line spacing: Double

Formatted: Font: 18 pt

Formatted: Indent: First line: 0"

Formatted: Font: (Default) Times New Roman, 12 pt

Formatted: Indent: Hanging: 0.5", Line spacing: Double

S5 file. Minimal datasets. Raw underlying data points for dose response study, and statistical analyses used to generate Table 3.

S6 file. Minimal datasets. Raw underlying data points for complete protection time study, and statistical analyses used to generate Figure 2 & Table 4.

S7 file. Minimal datasets. Raw underlying data points for complete protection time study, and statistical analyses used to generate Figure 1, Table 5 & 6.

S8 file. Minimal datasets. Raw underlying data points for correlation analysis between most abundant chemical compounds and repellent efficacy of essential oils.

A point-by-point response to comments of academic editor and the reviewers on our manuscript submission

Dear Prof. Mansureh Ghavam (Academic Editor),

We appreciate the constructive feedbacks provided by the academic editor and the two reviewers. We addressed the points raised and believe the changes improved the manuscript. The manuscript is revised and updated in response to the comments given by the academic editor and the two reviewers. The files were prepared based on the PLOS ONE's style and uploaded along with the revised manuscript.

Point-by-point responses to the comments by the academic editor in the editor's letter

Comment 1: Please include the following items when submitting your revised manuscript:

- A letter that responds to each point raised by the academic editor and reviewer(s). You should upload this letter as a separate file labeled 'Response to Reviewers'.
- A marked-up copy of your manuscript that highlights changes made to the original version. You should upload this as a separate file labeled 'Revised Manuscript with Track Changes'.
- An unmarked version of your revised paper without tracked changes. You should upload this as a separate file labeled 'Manuscript'.

Response: Thank you for the remark. **The following changes are made**

- A point-by-point response is given to each point raised by the academic editor and reviewer(s) as a letter and uploaded in a separate file.
- A marked-up copy of our manuscript that highlights changes made to the original version is uploaded as a separate file labeled "Revised Manuscript with Track Changes"
- Unmarked version of our revised manuscript without tracked changes is uploaded as a separate file labeled "Manuscript".

Comment 2: If you would like to make changes to your financial disclosure, please include your updated statement in your cover letter.

Response: Thank you for the remark. An updated statement of our financial disclosure was included in the cover letter and uploaded.

Comment 3: Guidelines for resubmitting your figure files are available below the reviewer comments at the end of this letter.

Response: Thank you for your remark. Figure files were updated according to the guidelines and uploaded.

Comment 4: If applicable, we recommend that you deposit your laboratory protocols in protocols.io to enhance the reproducibility of your results. Protocols.io assigns your protocol its own identifier (DOI) so that it can be cited independently in the future.

Response: Thank you for the recommendations. We used publicly available standardized WHO protocol and cited it in methods section **(page 6, lines 128)**. We believe this ensures the reproducibility our results.

Comment 5: Please ensure that your manuscript meets PLOS ONE's style requirements, including those for file naming.

Response: We examined the PLOS ONE style requirements and the formatting sample given. We have modified our paper to meet all style requirements.

Comment 6: In your Methods section, please provide additional information regarding the permits you obtained for the work. Please ensure you have included the full name of the authority that approved the field site access and, if no permits were required, a brief statement explaining why.

Response: Thank you for the comment. Additional information including the full name of the authority that approved the field site access is provided in method section of the revised manuscript **(page 6, lines 118-123)** after the comments.

Comment 7: We note that you have provided funding information that is not currently declared in your Funding Statement. However, funding information should not appear in the Acknowledgments section or other areas of your manuscript. We will only publish funding information present in the Funding Statement section of the online submission form.

Please remove any funding-related text from the manuscript and let us know how you would like to update your Funding Statement.

Response: We thank you for the correction. We have made the suggested correction and provided the funding information in the Funding Statement Section of the online submission form removed all funding information from the revised manuscript and our amended funding information was provided in our revised cover letter to include the correct grant information and specified the role of the funders as required following the comments.

Comment 8: Please state what role the funders took in the study. If the funders had no role, please state: "The funders had no role in study design, data collection and analysis, decision to publish, or preparation of the manuscript."

Response: Thank you for the comment. The role of the funder is corrected in the our revised cover letter after the comment.

Comment 9: We note that one or more of the authors is affiliated with the funding organization, indicating the funder may have had some role in the design, data collection, analysis or preparation of your manuscript for publication; in other words, the funder played an indirect role through the participation of the co-authors. If the funding organization did not play a role in the study design, data collection and analysis, decision to publish, or preparation of the manuscript and only provided financial support in the form of authors' salaries and/or research materials, please do the following:

1. Review your statements relating to the author contributions, and ensure you have specifically and accurately indicated the role(s) that these authors had in your study. These amendments should be made in the online form.
2. Confirm in your cover letter that you agree with the following statement, and we will change the online submission form on your behalf:

“The funder provided support in the form of salaries for authors [insert relevant initials], but did not have any additional role in the study design, data collection and analysis, decision to publish, or preparation of the manuscript. The specific roles of these authors are articulated in the ‘author contributions’ section.

Response: Thank you for the comments. Amendment on the funding statement was made in the revised cover letter and we have confirmed that we agree with these statements relating to the author contributions.

Comment 10: If the reviewer comments include a recommendation to cite specific previously published works, please review and evaluate these publications to determine whether they are relevant and should be cited. There is no requirement to cite these works unless the editor has indicated otherwise.

Response: We thank you for the guidance regarding additional citations. No comments were made by the reviewers to cite previously published works and hence no additional citations were made in the revised manuscript.

Additional Editor Comments:

Authors are requested to consider all the comments of the reviewers and prepare a point-by-point response. In addition, the following should be noted:

Comment 11: Clarify the doses applied in the first series of experiments and how the results of this bioassay were used in the second experiment for the duration of protection.

Response: We thank you for the opportunity to clarify our experimental design. In the revised manuscript, we have clarified the rationale behind our dosing strategies for both experimental phases. In the first phase, a dose-response bioassay was conducted using essential oil concentrations from 1% to 10% to establish the median (ED_{50}) and 99% (ED_{99}) effective doses. Because the calculated ED_{99} values for the oils fell below a 10% concentration, this specific range provided the logical baseline for selecting the target doses applied in the second experiment. As a result, the second series of experiments employed a fixed concentration of 10% for all essential oils whose calculated ED_{99} values fell below this limit and a 20% concentration of each oil for comparisons. These specific doses were selected to establish a uniform baseline benchmark across all formulations, while ensuring an adequate chemical reservoir on the skin surface to accurately measure the maximum duration of repellency. The materials and methods section **(page 8, lines 177-188)** of the revised manuscript has now been updated in response to the comments to reflect these changes.

Comment 12: The repellent properties of any of the compounds identified in the essential oils should be presented. The correlation between the chemical component and the mosquito repellent effect was not investigated.

Response: We appreciate for this critical comment. Correlation analysis was performed between the chemical component and mosquito repellent effect of on the most abundant compounds (>10%) and incorporated in the revised manuscript **(page 11, line 228-241).** We have also revised the discussion section **(Page 23, Lines 332-344)** in the revised manuscript to incorporate the review of the existing literature on the known repellent compounds identified in our GC-MS analysis following the feedback.

Comment 13: Finally, a data analysis should be performed to determine the potential synergistic effects of the essential oils.

Response: We thank you very much for this important comment. We accepted the comment and performed statistical comparison between the effect of individual oils and their respective blends. A non-parametric Kruskal-Wallis H test followed by Dunn's post hoc test was computed to compare the protection times across all treatment groups. This analysis allows us to determine if the blends provided significantly higher protection than the individual oils from which they were composed. We incorporated these changes in the results section **(Page 21, Lines 303-310 and Table 6)** of the revised manuscript. Corresponding, revision is made in the discussion section to explain synergistic effects **(Page 27, Lines 410-426)**.

Comment 14: It is also recommended that the writing be professionally improved in terms of English and nativeness.

Response: Thank you very much for this critical and helpful recommendation. We have accepted the feedback and **tried to edit** the text so that it is grammatically correct and professionally sound.

Point-by-Point responses to the reviewer's comments

Comments to the Author

1. Is the manuscript technically sound, and do the data support the conclusions?

Reviewer #1: Yes

Reviewer #2: Partly

2. Has the statistical analysis been performed appropriately and rigorously?

Reviewer #1: Yes

Reviewer #2: Yes

3. Have the authors made all data underlying the findings in their manuscript fully available?

Reviewer #1: Yes

Reviewer #2: Yes

4. Is the manuscript presented in an intelligible fashion and written in standard English?

Reviewer #1: No

Reviewer #2: Yes

Reviewer #1

General comment: The authors investigated the mosquito repellent effects of five essential oils that extracted from Croton macrostachyus, Echinops Kebericho, Eucalyptus globulus, and Juniperus procera against Anopheles arabiensis. The experimental results showed that all the five essential oils exhibit significant repellent properties. However, only the leaf and seed oils from C. macrostachyus meet the criteria by Environmental Protection Agency's registration. This research is interesting to the readerships of the PLoS One.

Response: We thank the reviewer for their positive assessment regarding the relevance of our work to the PLOS readership. We are encouraged by their interest in our findings.

Specific comments

Comment 1: Some specific comments are as follows: The English writing need to be improved.

Response: We appreciate for the constructive feedback. The manuscript has now been revisited to improve its readability, clarity, grammar and flow of idea. We have rephrased.

Comment 2: The chemical component of the five essential oils were analysed based on GC-MS. However, the correlations between the chemical component and the mosquito repellent effect were not investigated.

Response: Thank you very much for the constructive comment. While our study did not involve testing individual constituents, we incorporated correlation analysis results between most abundant chemical compounds and mosquito repellents effect **(page 11, line 228-241)** and made

corresponding discussion points in the discussion section after the comment **(Page 23, Lines 332-344).**

Comment 3: Rewrite the abstract.

Response: Thank you very much for the constructive comment. The abstract is re-written in response to the comments.

Comment 4: Vector graphics should be provided in Figures 1 to 3.

Response: Thank you for the comment. Vector graphics is depicted in figures 1 to 3 in the revised manuscript.

Comment 5: Revise the unit symbol of temperature to ℃.

Response: Thank you for the correction. The unit symbol of temperature has now been revised to ℃.

Comment 6: Use abbreviation for Latin names of the species after they are full named in the manuscript.

Response: Thank you for the feedback. Latin names of the species after they are full named are abbreviated in the revised version of the manuscript.

Reviewer #2

Dear authors, this paper deals with the determination of repellent properties of essential oils from 5 plant species against Anopheles arabiensis. Herein you will find my comments for your consideration.

Comment 1: Line 59: Do EOS really disrupt natural ecosystems? Please clarify how.

Response: Thank you for relevant question. We have now clarified the concern in the revised manuscript with relevant citation.

Comment 2: The induction of insecticide resistance is not relevant for repellents, so please remove it.

Response: We accepted the suggestion and removed the mention of insecticide resistance to maintain the focus on repellents.

Comment 3: Here it would be better to find some references for the potential of negative effects of synthetic repellents to humans.

Response: Thank you for the comment. We included additional reference indicating potential human health risks, including skin irritation in the revised version of the manuscript **(page 3, line 60-61).**

Comment 4: Line 73: use “repellent” not “repulsive”. Also, in the scope you have to mention the tested plant species.

Response: Thank you so much for the suggestion. The word “repulsive” is replaced by “repellent” in the revised manuscript.

Comment 5: Lines 160-169: The applied doses should be clarified. It seems that you applied one dose of each essential oil in µL per cm2. Is the range 0.86-0.98 µL referring to the amount of applied EO per cm2 due to differences in the skin area among the volunteers?

Response: We appreciate the reviewer's critical insight. “Because the skin surface area of the forearms differed among the participants, we have calculated the specific area for each volunteer and adjusted the volume of the repellent accordingly. The approach has now been revised and changes highlighted **(Page 8, lines 168-169)** in the revised manuscript following the comment.

Comment 4: What was the time interval between the application of cumulative doses? This may have affected the repellent potency due to evaporation. Hence, the repellent result may be different between one high dose and cumulative low doses that give the same high dose.

Response: Thank you so much for your concern. We followed the WHO (2009) dose-response bioassay recommendations, which involved applying incremental cumulative doses to the same forearm. To reduce the influence of evaporation on the results, the time between applications was kept to a minimum, with the exposure period for each dose lasting only 30 seconds, and the response was recorded immediately after each application to ensure accuracy. We have now addressed this in method section of the revised manuscript **(page 8, lines 173-174).**

Comment 5: The exposure time of treated arm to mosquitoes for the estimation of effective dose is not clarified.

Response: Thank you for your remark. The statement "Based on the WHO (2009) guidelines for dose-response bioassays, the exposure time for each incremental dose was 30 seconds" **(Page 8, lines 174).**

Comment 6: Why did you use 100 mosquitoes for the estimation of effective dose and 200 mosquitoes for the duration of protection?

Response: Thank you for the insight. The number of mosquitoes used in each test was based on the WHO guidelines (WHO, 2009). We included reference of the guideline in the methods section of the revised manuscript.

Comment 7: Line 187: In the statistical analysis the post hoc test should be clarified.

Response: Thank you for your feedback. The phrase "The Dunn's post hoc test" is added in the revised version of the manuscript **(page 9, line 203-205).**

Comment 8: A major limitation of this study is that no bioassay was conducted with the major compounds of the oils to identify potent repellent chemicals against mosquitoes and explain the action of the essential oil.

Response: We appreciate the reviewer's concern. However, the study assessed efficacy of the whole essential oil and synergistic blends of oils for practical application as a natural repellent. We attempted to describe limitation to highlight future research on bioactivity of individual compounds.

Comment 9: Table 3: The expression of the dose rate in µL/cm2 should be provided in the legend. Please confirm if this is the correct dose rate expression.
Response: Thank you for your insight. The caption of Table 3 is revised as "1.67 µL/cm^2" in the revised version of the manuscript to ensure clarity and reproducibility. We also confirmed that µL/cm^2 is the correct expression for the dose rate in this study.

Comment 10: Table 4 & 5. Remove the columns with P<0.001. The reference in the main text is sufficient. The applied concentrations should be clarified in the legends of the tables.

Response: Thank you for the suggestion. the suggested revision is made in the tables 4-6. Furthermore, the legends of tables 4 & 5 are revised in line with the applied concentrations (10% and 20%) and dosage rate (µL/cm²).

Comment 11: For me it is not clear how the results from the first trial for the estimation of effective dose are correlated with the next trial for the estimation of protection time in terms of applied doses.

Response: We appreciate to the reviewer for the opportunity to further explain the link between the two experiments. The first trial (effective dose estimation) served as the potency baseline to inform the second trial (protection duration). The first trial determined that the **ED_{99}** (the minimum dose for near-total protection at the moment of application). However, because essential oils are naturally volatile and quickly evaporate over time, this minimum dose should be slightly higher than ED_{99} to be adequate for measuring the maximum protection duration. As a result, for the second trial, we normally used a fixed concentration of 10% for all oils. Furthermore, doubling (20%) concentrations were used to confirm, how the concentration affects the duration of protection, as well as to compare to the standard 20% DEET employed as a positive control in accordance with the WHO guideline. The materials and methods section **(page 8, lines 177-188)** of the revised manuscript has now been updated to reflect the changes made for the clarity.

Comment 12: Normally, the most potent doses from the first trial should have been used for the protection time assays. However, for the protection time you tested 10% and 20% concentrations for all EOs no matter what the ED99 was estimated from the first bioassay.

Response: We thank the reviewer for these important methodological points. While using specific ED99 values is a common approach, we selected a fixed concentrations at 10 and 20% for the duration of protection bioassays in this investigation for the following key assumptions:

- Very high volatility nature of essential oils: It is necessitated to be optimized the minimum effective dosage to ensure sufficient concentrations for assessing long-term protection duration.

- Standardization and Comparability: In the current study, the experimental design incorporated a standardized head-to-head comparison to assess the residual efficacy and stability by testing all essential oils and their blends at the same concentrations. This approach minimizes confounding factors, ensuring that the observed protection time is a direct representation of the oils' inherent persistence rather than variations in initial dosage.
- Translational relevance: The selection of these specific concentrations was intended to bridge the gap between laboratory screening and practical application.

Comment 13: Lines 356-358: You mention the comparison between single and blended EOs, but no statistical comparison is made in this perspective. I would suggest elaborating more on your data by comparing the single and blended EOs to identify potential synergistic effects and support the relevant discussion.

Response: Thank you so much for this constructive suggestion. We have accepted the comment and appropriate statistical comparison has now been conducted and presented in **Table 6** of the revised manuscript after the comment.
