# Supplementary material for: Repellency and chemical composition of essential oils from four medicinal plants against Anopheles arabiensis (Diptera: Culicidae) under laboratory conditions
Source: PLoS One. 2026 Sep 15;21(9):e0358040. doi: 10.1371/journal.pone.0358040 (PMC13577348; doi:10.1371/journal.pone.0358040)
Supplement: Supplementary file 5 [file pone.0358040.s009.docx]

3 June 2026

Dear Author

The revised manuscript has improved considerably and addresses most of the concerns raised during the initial review. The study investigates the repellency of selected essential oils against Anopheles arabiensis, an important malaria vector, and provides valuable information on plant-based mosquito repellents with potential applications in vector control.

The inclusion of GC-MS analysis, dose-response assessment, complete protection time evaluation, and investigation of blended oil effects strengthens the scientific merit of the work. The revised statistical analyses are more appropriate and clearly presented, and the discussion has been improved by relating the findings to existing literature.

However, a few minor issues should still be considered before publication:

1. The manuscript would benefit from additional clarification regarding the practical implications of laboratory-based protection times under field conditions.
2. The discussion of the correlation between chemical constituents and repellency should be interpreted cautiously, as correlation does not necessarily imply causation.
3. Some language and grammatical issues remain throughout the manuscript and should be corrected through careful proofreading.
4. Future studies should include skin safety/toxicity assessments and field validation to support the development of these oils as commercial repellents.

Subject to minor editorial revisions, I recommend the manuscript for publication.

Sd/

ASHISH VALA
