# Supplementary material for: Repellency and chemical composition of essential oils from four medicinal plants against Anopheles arabiensis (Diptera: Culicidae) under laboratory conditions
Source: PLoS One. 2026 Sep 15;21(9):e0358040. doi: 10.1371/journal.pone.0358040 (PMC13577348; doi:10.1371/journal.pone.0358040)
Supplement: Supplementary file 6 [file pone.0358040.s011.docx]

**A point-by-point response to comments of academic editor and the reviewers on our manuscript submission**

**Dear Prof. Mansureh Ghavam (Academic Editor),**

We appreciate the constructive feedbacks provided by the academic editor and the two reviewers. We addressed the points raised and believe the changes improved the manuscript. The manuscript is revised and updated in response to the comments given by the academic editor and the two reviewers. The files were prepared based on the PLOS ONE's style and uploaded along with the revised manuscript.

**Point-by-point responses to the comments by the academic editor in the editor’s letter**

**Comment 1:** Please include the following items when submitting your revised manuscript:

- A letter that responds to each point raised by the academic editor and reviewer(s). You should upload this letter as a separate file labeled 'Response to Reviewers'.
- A marked-up copy of your manuscript that highlights changes made to the original version. You should upload this as a separate file labeled 'Revised Manuscript with Track Changes'.
- An unmarked version of your revised paper without tracked changes. You should upload this as a separate file labeled 'Manuscript'.

**Response:** Thank you for the remark. **The following changes are made**

- A point-by-point response is given to each point raised by the academic editor and reviewer(s) as a letter and uploaded in a separate file.
- A marked-up copy of our manuscript that highlights changes made to the original version is uploaded as a separate file labeled “Revised Manuscript with Track Changes”
- Unmarked version of our revised manuscript without tracked changes is uploaded as a separate file labeled “Manuscript”.

**Comment 2:** If you would like to make changes to your financial disclosure, please include your updated statement in your cover letter.

**Response:** Thank you for the remark. An updated statement of our financial disclosure was included in the cover letter and uploaded.

**Comment 3:** Guidelines for resubmitting your figure files are available below the reviewer comments at the end of this letter.

**Response:** Thank you for your remark. Figure files were updated according to the guidelines and uploaded.

**Comment 4:** If applicable, we recommend that you deposit your laboratory protocols in [protocols.io](http://protocols.io/) to enhance the reproducibility of your results. Protocols.io assigns your protocol its own identifier (DOI) so that it can be cited independently in the future.

**Response:** Thank you for the recommendations. We used publicly available standardized WHO protocol and cited it in methods section **(page 6, lines 128)**. We believe this ensures the reproducibility our results.

**Comment 5:** Please ensure that your manuscript meets PLOS ONE's style requirements, including those for file naming.

**Response:** We examined the PLOS ONE style requirements and the formatting sample given. We have modified our paper to meet all style requirements.

**Comment 6:** In your Methods section, please provide additional information regarding the permits you obtained for the work. Please ensure you have included the full name of the authority that approved the field site access and, if no permits were required, a brief statement explaining why.

**Response:** Thank you for the comment. Additional information including the full name of the authority that approved the field site access is provided in method section of the revised manuscript **(page 6, lines 118-123)** after the comments.

**Comment 7:** We note that you have provided funding information that is not currently declared in your Funding Statement. However, funding information should not appear in the Acknowledgments section or other areas of your manuscript. We will only publish funding information present in the Funding Statement section of the online submission form. 
Please remove any funding-related text from the manuscript and let us know how you would like to update your Funding Statement.

**Response:** We thank you for the correction. We have made the suggested correction and provided the funding information in the Funding Statement Section of the online submission form removed all funding information from the revised manuscript and our amended funding information was provided in our revised cover letter to include the correct grant information and specified the role of the funders as required following the comments.

**Comment 8:** Please state what role the funders took in the study.  If the funders had no role, please state: "The funders had no role in study design, data collection and analysis, decision to publish, or preparation of the manuscript."

**Response:** Thank you for the comment. The role of the funder is corrected in the our revised cover letter after the comment.

**Comment 9:** We note that one or more of the authors is affiliated with the funding organization, indicating the funder may have had some role in the design, data collection, analysis or preparation of your manuscript for publication; in other words, the funder played an indirect role through the participation of the co-authors. If the funding organization did not play a role in the study design, data collection and analysis, decision to publish, or preparation of the manuscript and only provided financial support in the form of authors' salaries and/or research materials, please do the following:
1. Review your statements relating to the author contributions, and ensure you have specifically and accurately indicated the role(s) that these authors had in your study. These amendments should be made in the online form.

2. Confirm in your cover letter that you agree with the following statement, and we will change the online submission form on your behalf:

“The funder provided support in the form of salaries for authors [insert relevant initials], but did not have any additional role in the study design, data collection and analysis, decision to publish, or preparation of the manuscript. The specific roles of these authors are articulated in the ‘author contributions’ section.

**Response:** Thank you for the comments. Amendment on the funding statement was made in the revised cover letter and we have confirmed that we agree with these statements relating to the author contributions.

**Comment 10:** If the reviewer comments include a recommendation to cite specific previously published works, please review and evaluate these publications to determine whether they are relevant and should be cited. There is no requirement to cite these works unless the editor has indicated otherwise.

**Response:** We thank you for the guidance regarding additional citations. No comments were made by the reviewers to cite previously published works and hence no additional citations were made in the revised manuscript.

**Additional Editor Comments:**

Authors are requested to consider all the comments of the reviewers and prepare a point-by-point response. In addition, the following should be noted:

**Comment 11:** Clarify the doses applied in the first series of experiments and how the results of this bioassay were used in the second experiment for the duration of protection.

**Response:** We thank you for the opportunity to clarify our experimental design. In the revised manuscript, we have clarified the rationale behind our dosing strategies for both experimental phases. In the first phase, a dose-response bioassay was conducted using essential oil concentrations from 1% to 10% to establish the median (ED_50_) and 99% (ED_99_) effective doses. Because the calculated ED_99_ values for the oils fell below a 10% concentration, this specific range provided the logical baseline for selecting the target doses applied in the second experiment. As a result, the second series of experiments employed a fixed concentration of 10% for all essential oils whose calculated ED_99_ values fell below this limit and a 20% concentration of each oil for comparisons. These specific doses were selected to establish a uniform baseline benchmark across all formulations, while ensuring an adequate chemical reservoir on the skin surface to accurately measure the maximum duration of repellency. The materials and methods section **(page 8, lines 177-188)** of the revised manuscript has now been updated in response to the comments to reflect these changes.

**Comment 12:** The repellent properties of any of the compounds identified in the essential oils should be presented. The correlation between the chemical component and the mosquito repellent effect was not investigated.

**Response:** We appreciate for this critical comment. Correlation analysis was performed between the chemical component and mosquito repellent effect of on the most abundant compounds (>10%) and incorporated in the revised manuscript **(page 11, line 228-241).** We have also revised the discussion section **(Page 23, Lines 332-344)** in the revised manuscript to incorporate the review of the existing literature on the known repellent compounds identified in our GC-MS analysis following the feedback.

**Comment 13:** Finally, a data analysis should be performed to determine the potential synergistic effects of the essential oils.

**Response:** We thank you very much for this important comment. We accepted the comment and performed statistical comparison between the effect of individual oils and their respective blends. A non-parametric Kruskal-Wallis H test followed by Dunn's post hoc test was computed to compare the protection times across all treatment groups. This analysis allows us to determine if the blends provided significantly higher protection than the individual oils from which they were composed. We incorporated these changes in the results section **(Page 21, Lines 303-310 and Table 6)** of the revised manuscript. Corresponding, revision is made in the discussion section to explain synergistic effects **(Page 27, Lines 410-426)**.

**Comment 14:** It is also recommended that the writing be professionally improved in terms of English and nativeness.

**Response:** Thank you very much for this critical and helpful recommendation. We have accepted the feedback and **tried to edit** the text so that it is grammatically correct and professionally sound.

**Point-by-Point responses to the reviewer’s comments**

**Comments to the Author**
1. Is the manuscript technically sound, and do the data support the conclusions?

Reviewer #1: Yes

Reviewer #2: Partly

2. Has the statistical analysis been performed appropriately and rigorously?

Reviewer #1: Yes

Reviewer #2: Yes

3. Have the authors made all data underlying the findings in their manuscript fully available?

Reviewer #1: Yes

Reviewer #2: Yes

4. Is the manuscript presented in an intelligible fashion and written in standard English?

Reviewer #1: No

Reviewer #2: Yes

**Reviewer #1**

**General comment:** The authors investigated the mosquito repellent effects of five essential oils that extracted from Croton macrostachyus, Echinops Kebericho, Eucalyptus globulus, and Juniperus procera against Anopheles arabiensis. The experimental results showed that all the five essential oils exhibit significant repellent properties. However, only the leaf and seed oils from C. macrostachyus meet the criteria by Environmental Protection Agency's registration. This research is interesting to the readerships of the PLoS One.

**Response:** We thank the reviewer for their positive assessment regarding the relevance of our work to the PLOS readership. We are encouraged by their interest in our findings.

**Specific comments**

**Comment 1:** Some specific comments are as follows: The English writing need to be improved.

**Response:** We appreciate for the constructive feedback. The manuscript has now been revisited to improve its readability, clarity, grammar and flow of idea. We have rephrased.

**Comment 2:** The chemical component of the five essential oils were analysed based on GC-MS. However, the correlations between the chemical component and the mosquito repellent effect were not investigated.

**Response:** Thank you very much for the constructive comment. While our study did not involve testing individual constituents, we incorporated correlation analysis results between most abundant chemical compounds and mosquito repellents effect **(page 11, line 228-241)** and made corresponding discussion points in the discussion section after the comment **(Page 23, Lines 332-344).**

**Comment 3:** Rewrite the abstract.

**Response:** Thank you very much for the constructive comment. The abstract is re-written in response to the comments.

**Comment 4:** Vector graphics should be provided in Figures 1 to 3.

**Response:** Thank you for the comment. Vector graphics is depicted in figures 1 to 3 in the revised manuscript.

**Comment 5:** Revise the unit symbol of temperature to ℃.

**Response:** Thank you for the correction. The unit symbol of temperature has now been revised to ℃.

**Comment 6:** Use abbreviation for Latin names of the species after they are full named in the manuscript.

**Response:** Thank you for the feedback. Latin names of the species after they are full named are abbreviated in the revised version of the manuscript.

**Reviewer #2**

Dear authors, this paper deals with the determination of repellent properties of essential oils from 5 plant species against Anopheles arabiensis. Herein you will find my comments for your consideration.

**Comment 1:** Line 59: Do EOS really disrupt natural ecosystems? Please clarify how.

**Response:** Thank you for relevant question. We have now clarified the concern in the revised manuscript with relevant citation.

**Comment 2:** The induction of insecticide resistance is not relevant for repellents, so please remove it.

**Response:** We accepted the suggestion and removed the mention of insecticide resistance to maintain the focus on repellents.

**Comment 3:** Here it would be better to find some references for the potential of negative effects of synthetic repellents to humans.

**Response:** Thank you for the comment. We included additional reference indicating potential human health risks, including skin irritation in the revised version of the manuscript **(page 3, line 60-61).**

**Comment 4:** Line 73: use “repellent” not “repulsive”. Also, in the scope you have to mention the tested plant species.

**Response:** Thank you so much for the suggestion. The word “repulsive” is replaced by “repellent” in the revised manuscript.

**Comment 5:** Lines 160-169: The applied doses should be clarified. It seems that you applied one dose of each essential oil in μL per cm2. Is the range 0.86-0.98 μL referring to the amount of applied EO per cm2 due to differences in the skin area among the volunteers?

**Response:** We appreciate the reviewer's critical insight. “Because the skin surface area of the forearms differed among the participants, we have calculated the specific area for each volunteer and adjusted the volume of the repellent accordingly. The approach has now been revised and changes highlighted **(Page 8, lines 168-169)** in the revised manuscript following the comment.

**Comment 4:** What was the time interval between the application of cumulative doses? This may have affected the repellent potency due to evaporation. Hence, the repellent result may be different between one high dose and cumulative low doses that give the same high dose.

**Response:** Thank you so much for your concern. We followed the WHO (2009) dose-response bioassay recommendations, which involved applying incremental cumulative doses to the same forearm. To reduce the influence of evaporation on the results, the time between applications was kept to a minimum, with the exposure period for each dose lasting only 30 seconds, and the response was recorded immediately after each application to ensure accuracy. We have now addressed this in method section of the revised manuscript **(page 8, lines 173-174).**

**Comment 5:** The exposure time of treated arm to mosquitoes for the estimation of effective dose is not clarified.

**Response:** Thank you for your remark. The statement “Based on the WHO (2009) guidelines for dose-response bioassays, the exposure time for each incremental dose was 30 seconds” **(Page 8, lines 174).**

**Comment 6:** Why did you use 100 mosquitoes for the estimation of effective dose and 200 mosquitoes for the duration of protection?

**Response:** Thank you for the insight. The number of mosquitoes used in each test was based on the WHO guidelines (WHO, 2009). We included reference of the guideline in the methods section of the revised manuscript.

**Comment 7:** Line 187: In the statistical analysis the post hoc test should be clarified.

**Response:** Thank you for your feedback. The phrase “The Dunn’s post hoc test” is added in the revised version of the manuscript **(page 9, line 203-205).**

**Comment 8:** A major limitation of this study is that no bioassay was conducted with the major compounds of the oils to identify potent repellent chemicals against mosquitoes and explain the action of the essential oil.

**Response:** We appreciate the reviewer's concern. However, the study assessed efficacy of the whole essential oil and synergistic blends of oils for practical application as a natural repellent. We attempted to describe limitation to highlight future research on bioactivity of individual compounds.

**Comment 9:** Table 3: The expression of the dose rate in μL/cm2 should be provided in the legend. Please confirm if this is the correct dose rate expression.

**Response:** Thank you for your insight. The caption of Table 3 is revised as “1.67 μL/cm^2”^ in the revised version of the manuscript to ensure clarity and reproducibility. We also confirmed that μL/cm^2^ is the correct expression for the dose rate in this study.

**Comment 10:** Table 4 & 5. Remove the columns with P<0.001. The reference in the main text is sufficient. The applied concentrations should be clarified in the legends of the tables.

**Response:** Thank you for the suggestion. the suggested revision is made in the tables 4-6. Furthermore, the legends of tables 4 & 5 are revised in line with the applied concentrations (10% and 20%) and dosage rate (μL/cm²).

**Comment 11:** For me it is not clear how the results from the first trial for the estimation of effective dose are correlated with the next trial for the estimation of protection time in terms of applied doses.

**Response:** We appreciate to the reviewer for the opportunity to further explain the link between the two experiments. The first trial (effective dose estimation) served as the potency baseline to inform the second trial (protection duration). The first trial determined that the **ED_99_** (the minimum dose for near-total protection at the moment of application). However, because essential oils are naturally volatile and quickly evaporate over time, this minimum dose should be slightly higher than ED_99_ to be adequate for measuring the maximum protection duration. As a result, for the second trial, we normally used a fixed concentration of 10% for all oils. Furthermore, doubling (20%) concentrations were used to confirm, how the concentration affects the duration of protection, as well as to compare to the standard 20% DEET employed as a positive control in accordance with the WHO guideline. The materials and methods section **(page 8, lines 177-188)** of the revised manuscript has now been updated to reflect the changes made for the clarity.

**Comment 12:** Normally, the most potent doses from the first trial should have been used for the protection time assays. However, for the protection time you tested 10% and 20% concentrations for all EOs no matter what the ED99 was estimated from the first bioassay.

**Response:** We thank the reviewer for these important methodological points. While using specific ED99 values is a common approach, we selected a fixed concentrations at 10 and 20% for the duration of protection bioassays in this investigation for the following key assumptions:

- Very high volatility nature of essential oils: It is necessitated to be optimized the minimum effective dosage to ensure sufficient concentrations for assessing long-term protection duration.
- Standardization and Comparability:  In the current study, the experimental design incorporated a standardized head-to-head comparison to assess the residual efficacy and stability by testing all essential oils and their blends at the same concentrations. This approach minimizes confounding factors, ensuring that the observed protection time is a direct representation of the oils' inherent persistence rather than variations in initial dosage.
- Translational relevance: The selection of these specific concentrations was intended to bridge the gap between laboratory screening and practical application.

**Comment 13:** Lines 356-358: You mention the comparison between single and blended EOs, but no statistical comparison is made in this perspective. I would suggest elaborating more on your data by comparing the single and blended EOs to identify potential synergistic effects and support the relevant discussion.

**Response:** Thank you so much for this constructive suggestion. We have accepted the comment and appropriate statistical comparison has now been conducted and presented in **Table 6** of the revised manuscript after the comment.
