# Supplementary material for: Repellency and chemical composition of essential oils from four medicinal plants against Anopheles arabiensis (Diptera: Culicidae) under laboratory conditions
Source: PLoS One. 2026 Sep 15;21(9):e0358040. doi: 10.1371/journal.pone.0358040 (PMC13577348; doi:10.1371/journal.pone.0358040)
Supplement: Supplementary file 7 [file pone.0358040.s012.docx]

**A point-by-point response to comments of academic editor and the reviewers on our revised manuscript submission**

**Dear Prof. Mansureh Ghavam (Academic Editor),**

We appreciate the constructive feedbacks provided by the academic editor and the two reviewers on our revised manuscript. We addressed the points raised and believe the changes further improved the manuscript. The manuscript is revised and updated in response to the comments given by the academic editor and the two reviewers.

**Point-by-point responses to the comments by the academic editor in the editor’s letter**

**Comment 1:** Please include the following items when submitting your revised manuscript:

- A letter that responds to each point raised by the academic editor and reviewer(s). You should upload this letter as a separate file labeled 'Response to Reviewers'.
- A marked-up copy of your manuscript that highlights changes made to the original version. You should upload this as a separate file labeled 'Revised Manuscript with Track Changes'.
- An unmarked version of your revised paper without tracked changes. You should upload this as a separate file labeled 'Manuscript'.

**Response:** Thank you for the remark. **The following changes are made**

- A point-by-point response is given to each point raised by the academic editor and reviewer(s) as a letter and uploaded in a separate file.
- A marked-up copy of our revised manuscript that highlights changes made to the previous version is uploaded as a separate file labeled “Revised Manuscript with Track Changes”
- Unmarked version of our revised manuscript without tracked changes is uploaded as a separate file labeled “Manuscript”.

**Comment 2:** If you would like to make changes to your financial disclosure, please include your updated statement in your cover letter.

**Response:** Thank you for the remark. No changes were made on our financial disclosure in the latest version of the manuscript.

**Comment 3:** Guidelines for resubmitting your figure files are available below the reviewer comments at the end of this letter.

**Response:** Thank you for your remark. Figure files were prepared according to the guidelines.

**Comment 4:** If applicable, we recommend that you deposit your laboratory protocols in [protocols.io](http://protocols.io/) to enhance the reproducibility of your results. Protocols.io assigns your protocol its own identifier (DOI) so that it can be cited independently in the future.

**Response:** Thank you for the recommendations. We used publicly available standardized WHO protocol to ensures the reproducibility of results.

**Comment 5:** If the reviewer comments include a recommendation to cite specific previously published works, please review and evaluate these publications to determine whether they are relevant and should be cited. There is no requirement to cite these works unless the editor has indicated otherwise.

**Response:** Thank you for the guidance. No comments were made by the reviewers to cite specific previously published works.

**Point-by-Point responses to the reviewer’s comments in the editor’s letter and revised manuscript**

**Comments to the Author**

1. If the authors have adequately addressed your comments raised in a previous round of review and you feel that this manuscript is now acceptable for publication, you may indicate that here to bypass the “Comments to the Author” section, enter your conflict-of-interest statement in the “Confidential to Editor” section, and submit your "Accept" recommendation.

Reviewer #3: All comments have been addressed

Reviewer #4: All comments have been addressed

2. Is the manuscript technically sound, and do the data support the conclusions?

Reviewer #3: Yes

Reviewer #4: Partly

3. Has the statistical analysis been performed appropriately and rigorously?

Reviewer #3: Yes

Reviewer #4: I Don't Know

4. Have the authors made all data underlying the findings in their manuscript fully available?

Reviewer #3: Yes

Reviewer #4: Yes

5. Is the manuscript presented in an intelligible fashion and written in standard English?

Reviewer #3: Yes

Reviewer #4: Yes

6. Review Comments to the Author

Please use the space provided to explain your answers to the questions above. You may also include additional comments for the author, including concerns about dual publication, research ethics, or publication ethics. (Please upload your review as an attachment if it exceeds 20,000 characters)

**Reviewer #3:** 3 June 2026

Dear Author,

The revised manuscript has improved considerably and addresses most of the concerns raised during the initial review. The study investigates the repellency of selected essential oils against *Anopheles arabiensis*, an important malaria vector, and provides valuable information on plant-based mosquito repellents with potential applications in vector control.

The inclusion of GC-MS analysis, dose-response assessment, complete protection time evaluation, and investigation of blended oil effects strengthens the scientific merit of the work. The revised statistical analyses are more appropriate and clearly presented, and the discussion has been improved by relating the findings to existing literature.

However, a few minor issues should still be considered before publication:

**Comment 1**. The manuscript would benefit from additional clarification regarding the practical implications of laboratory-based protection times under field conditions.

**Response:** Thank you for the constructive suggestion. We agree that laboratory-derived complete protection times (CPT) are optimal, highly controlled settings that do not accurately replicate the complicated dynamics of field conditions. As a result, our laboratory CPT values serve as a benchmark for ranking potential of the oils. Following the feedback, we further clarified this reasoning in a discussion section of the updated version of the manuscript.

**Comment 2**. The discussion of the correlation between chemical constituents and repellency should be interpreted cautiously, as correlation does not necessarily imply causation.

**Response:** Thank you for your critical comment. In response to the feedback, we accepted and the discussion section has been amended in the revised manuscript (page 26, line 371-374).

**Comment 3**. Some language and grammatical issues remain throughout the manuscript and should be corrected through careful proofreading.

**Response:** Thank you for your remark. We accepted the comment, and the revised manuscript has now been checked line by line and edited for grammatical errors and sentence structure.

**Comment 4**. Future studies should include skin safety/toxicity assessments and field validation to support the development of these oils as commercial repellents.

**Response:** Thank you for your critical suggestion. We have changed the conclusion section of the revised manuscript to mention that future studies must include skin toxicity assessment and field validation in response to the feedback.

**Comment 5.** Subject to minor editorial revisions, I recommend the manuscript for publication.

**Response:** We appreciate the constructive comments and for recommending our manuscript for publication. We handled all adjustments to ensure that the text has now been fully refined in the revised version of the paper.

**Reviewer #4:**

**Comment 1.** This manuscript is about the potential of selected essential oil (EO) from different plant species and the effectiveness of these EO to repel mosquitoes. This is the second manuscript that get reviewed. Overall, the manuscript writing flow is good and easy to follow. Nevertheless, there are few issues that author needs to address.

**Response:** We thank the reviewer for their positive assessment of our manuscript's flow and clarity. We attempted to address each of the specific points noted below in order to further improve the overall clarity of the text in the revised manuscript following the feedback.

**Comment 2**. The title can be further refined to include a complex system too such as food because later part of the manuscript interaction in a complex system was highlighted.

**Response:** We appreciate the reviewer's constructive feedback. The title has now been updated in response to the comment in the revised version of the manuscript.

**Comment 3**. The manuscript is written in a good flow but needs to be further improved.

Response: We thank the reviewer for their positive feedbacks. The revised manuscript has now been thoroughly edited and further improved for clarity and language.

**Comment 4**. The abstract needs to further improve. The current abstract too lengthy and contains unnecessary information.

**Response:** We thank the reviewer for providing valuable feedback. The abstract has now been revised to remove unnecessary information and concentrate on the core methodology and key findings.

**The detailed comment is in the manuscript or specific comment section below.**

**Comment 5**. The introduction background is good. However, the author should highlight the current successful EO used commercially for insect repellant. There are many effective EO that are commercially available or proven to be a potent insect repellent such as lemon grass.

**Response:** We thank the reviewer for these critical comments. We have revised the introduction section to highlight some successful plant-based repellents.

**Comment 6**. The problem statement is fine.

**Response:** We appreciate the reviewer for evaluating and confirming the clarity of the problem statement.

**Comment 7**. The objective needs to be clear and detail. The optimization of effective dose first determines, followed by trial on human subject.

**Response:** We appreciate the reviewer's critical comment. The objective section in the revised version of the manuscript has been amended in response to the feedback to clearly present the two-phase experiments, indicating that the effective dosage was determined first, followed by protection time test on human subjects.

**Comment 8**. The methodology section needs a proper explanation of the design. The sample of 5 people is not enough for scientific work. Nevertheless, the replication and repetition must be robust to support the findings.

**Response:** Thank you for this critical concern regarding the sample size. We agree that larger sample sizes are required to maximize statistical power. We have revised the methods section in the latest version of the revised manuscript to clarify this sample size in response to the feedback. According to WHO 2009 guidelines, sample sizes must allow for statistical analysis while ethically minimizing human exposure to mosquito bites and unrefined formulations until final product safety profiles are developed. This preliminary screening laboratory work employed a rigorous repeated-measures approach, using a limited five human volunteers including evaluation of numerous concentrations and exposure windows on the same subjects, resulting in a high-density matrix of intra-subject data points. This data density provided the statistical power necessary to confidently determine both dose-response curves (effective doses) and complete protection time.

**Comment 9**. The effective dosage and the applicable dosage are two different values and not interconnected to each other. This makes the result of the effective dosage irrelevant for the application.

**Response:** We thank the reviewer and accept the comment that effective dosage and applicable dosage are distinct values. However, using a standardized, higher concentration in Phase two experiment was a deliberate methodological choice, as this sequential approach follows standard WHO guidelines. Because essential oils are highly volatile, measuring complete protection time at that immediate baseline threshold would lead to rapid volatilization and instant failure. Therefore, a standardized fixed concentration was deliberately chosen for phase two to ensure a uniform chemical reservoir on the skin, enabling a fair comparative assessment of the oils' duration of protection. We have clarified this in the revised manuscript.

**Comment 10**. In the discussion, the reason for these differences was due to high volatility of the compounds. Is there any measurement to measure the rate of volatility of the EO applied at the effective dosage?

**Response:** Thank you for this insightful question. In this specific study, we did not directly measure the physical evaporation or volatilization rates. However, the high volatility and rapid evaporation nature of essential oils are thoroughly documented in literature. A longevity study is mathematically unfeasible without a fixative. We have included this in the discussion section of revised manuscript to support the justification.

**Comment 11**. The discussion is fit and explain the result. Suggest relating with other EO that known to have insect repellency such as peppermint EO, clove EO and etc.

**Response:** We thank the reviewer for the positive assessment. We accept the suggestion and have revised the discussion section in the revised manuscript to contextualize our findings alongside other well-known, potent insect-repellent essential oils to provide a broader comparative perspective (page 28, line 413-418).

**Comment 12**. Conclusion is concise but can be further improved. Highlight the major compounds that found in the EO extracted that potentially contribute to the repellency from mosquitoes.

**Response:** We thank the reviewer for this constructive suggestion. We have revised and the major chemical compounds has now been highlighted in the conclusion section of the revised manuscript.

**Specific comment**s
**Comment 13**. Line 24-26; Please remove this from abstract. It should be highlight in the methodology statistical analysis.

**Response:** We thank the reviewer for this correction. We removed the statistical details from the abstract following the feedback.

**Comment 14**. Line 19- I don’t notice any specific pathway that been highlighted in the manuscript. Do you mean redox pathway?

**Response:** We thank the reviewer for pointing this out. This study focuses on the behavioral screening of repellent efficacy including complete protection time and landing inhibition and did not investigate underlying physiological mechanisms, such as the redox pathway.

**Comment 15**. Line 101-114; State how the compound was identified?

**Response:** We thank the reviewer for this request. We have updated the GC-MS analysis subsection in the Methodology (page 6 and line 124-131) in response to the comment.

**Comment 16**. which library and software used for the identification?

**Response:** We thank for the comment. We have added the specific details to the methodology following the feedback.

**Comment 17**. Any internal standard used since the quantification based on area under the curve?

**Response:** We thank the reviewer for this question. There was no internal standard employed; instead, quantification was done using relative peak area percentage normalization, which expresses the area of each individual peak as a direct proportion of the total combined area of all detected peaks.

**Comment 18**. Line 131-132; Additional for 10% too? I think this is a typing error. The 10% already mentioned. delete the words and remain the 20% only.

**Response:** Thank you for the comment. The redundant 10% concentration in the amended manuscript has now been deleted.

**Comment 19**. Line 142; state the unit as (w/v) or (v/v)

**Response:** We thank you for pointing this out. We have corrected the concentration units.

**Comment 20**. Line 145; this is very low number of samples for human test

**Response:** We thank for this note. As detailed in our response to the previous comment, the methodology section has been revised to reflect these changes (page 8 and line 164-169).

**Comment 21**. Line 149; Please write down the formula for the calculation. Highlight this and make this as equation 1.

**Response:** Thank you for the comment. The formula has now been formatted and highlighted as Equation 1 (page 8 and line 173-178).

**Comment 22**. Line 162-169; Is this the same mosquito or a different group of mosquitos? I mean the same 100 mosquito? Why this is not done simultaneously?

**Response:** We thank the reviewer for this important question. Fresh, separate 100 host-seeking female mosquitoes were used for each distinct treatment. Moreover, the bioassays were conducted sequentially rather than simultaneously on a human subject to prevent the cross-contamination.

**Comment 23**. Line 179; state the concentration of DEET use as positive control.

**Response:** We thank for the comment. We have updated the text in response to the comment.

**Comment 24**. Line 189-190; Please rephrase this. Do you mean mosquito landed after the 30 minutes rest?

**Response:** Thank you. We have rephrased the statement to clarify methods.

**Comment 25**. Line 209; label this formula as equation 2.

**Response:** We thank you and have updated the text in response to the comment.

**Comment 26**. Line 218; Insert Table 1 at line 218.

**Response:** We thank the reviewer and have moved Table 1 to the proper location following the comment.

**Comment 27**. Line 231 & 237; use symbol for delta & use symbol for gamma

**Response:** We thank you, and the spelled-out names have been replaced with symbols.

**Comment 28**. Line 257-258; Based on the data in Table 3, they are not significant value. What are these significant values (p-values) representing? Is this comparing between ED50& ED99 or between the sample? It was not mentioned in the paragraph.

**Response:** We thank the reviewer for pointing out this lack of clarity. The non-significant p values listed in Table 3 represent the Pearson chi-square goodness-of-fit test for the probit regression model, rather than a direct comparison between ED_50_ and ED_99_. (page 21, line 280).

**Comment 29**. Line 286, 310, 312; Table 4, Table 5 and Table 6, Statistical indicator explanation in each table. a stand for what? significant different? They should mention different small letter mean there are statistical differences.

**Response:** We thank the reviewer for making this correction. Tables 4, 5, and 6 in the revised manuscript has been now included footnote for the statistical indicators.

**Comment 30**. Line 327 & 329; Please use symbol for gamma.

**Response:** We thank the reviewer and have updated the text in response to the comment.

**Comment 31**. Line 338-340; The reference focuses on the toxicity of these compounds on human. The right paper to cite is on the effect of insects. Either this is also toxic to insect or it just repels the mosquitoes due to the smell. Suggest finding a more relevant reference.

**Response:** We appreciate and accept the critical correction. We revised the references with more relevant material.

**Comment 32**. Line 362-364; What is the difference between these approaches?

**Response:** We thank the reviewer. First landing: The protection period stops the instant a mosquito arrives on the treated skin, even if it does not bite. First bite/probe: Protection only ends when a mosquito probes or feeds on the skin. The current study employed mosquito landing as a failure criterion since it is the safest and most rigorous baseline for complete protection time (CPT), while ethically protecting volunteers from unnecessary bites. A clarifying sentence has been included into the revised methods section in the latest version of the manuscript.

**Comment 33**. Line 642-643; Reference 53, as mentioned earlier, please find a relevant reference for the effect of toxicity on insects.

**Response:** We appreciate the reviewer's recommendation. Reference 53 and the associated information has been deleted from the revised manuscript.

**Comment 34**. Line 664-665; In the text, this reference state EPA standard but this reference is not from EPA. This is secondary source citation. Suggest to cite both references.

**Response:** We appreciate the reviewer for pointing out this citation concern. We have accepted the comment and the amended manuscript has been updated following the feedback.
