# Supplementary material for: Repellency and chemical composition of essential oils from four medicinal plants against Anopheles arabiensis (Diptera: Culicidae) under laboratory conditions
Source: PLoS One. 2026 Sep 15;21(9):e0358040. doi: 10.1371/journal.pone.0358040 (PMC13577348; doi:10.1371/journal.pone.0358040)
Supplement: Supplementary file 8 [file pone.0358040.s013.docx]

**A point-by-point response to comments of academic editor and the reviewers on our revised manuscript submission**

**Dear Prof. Mansureh Ghavam (Academic Editor),**

We appreciate the constructive feedback provided by the academic editor and three reviewers on our revised manuscript. We addressed the points raised and believe the changes significantly improved the current version of the revised manuscript.

**Point-by-point responses to the comments by the academic editor in the editor’s letter**

**Comment 1:** Please include the following items when submitting your revised manuscript:

- A letter that responds to each point raised by the academic editor and reviewer(s). You should upload this letter as a separate file labeled 'Response to Reviewers'.
- A marked-up copy of your manuscript that highlights changes made to the original version. You should upload this as a separate file labeled 'Revised Manuscript with Track Changes'.
- An unmarked version of your revised paper, without tracked changes. You should upload this as a separate file labeled 'Manuscript'.

**Response:** Thank you for the remark. **The following changes are made**

- A point-by-point response is given to each point raised by the academic editor and reviewer(s) as a letter and uploaded in a separate file.
- A marked-up copy of our revised manuscript that highlights changes made to the previous version is uploaded as a separate file labeled “Revised Manuscript with Track Changes”
- An unmarked version of our revised manuscript without tracked changes is uploaded as a separate file labeled “Manuscript”.

**Comment 2:** If you would like to make changes to your financial disclosure, please include your updated statement in your cover letter.

**Response:** Thank you for the remark. No changes were made to our financial disclosure in the latest version of the revised manuscript.

**Comment 3:** Guidelines for resubmitting your figure files are available below the reviewer comments at the end of this letter.

**Response:** Thank you for your remark. Figure files were prepared according to the guidelines.

**Comment 4:** If applicable, we recommend that you deposit your laboratory protocols in [protocols.io](http://protocols.io/) to enhance the reproducibility of your results. Protocols.io assigns your protocol its own identifier (DOI) so that it can be cited independently in the future.

**Response:** Thank you for the recommendations. We used a publicly available standardized WHO protocol to ensure the reproducibility of results.

**Comment 5:** If the reviewer comments include a recommendation to cite specific previously published works, please review and evaluate these publications to determine whether they are relevant and should be cited. There is no requirement to cite these works unless the editor has indicated otherwise.

**Response:** Thank you for the guidance. The reviewers did not request citations for specific previously published works.

**Point-by-Point responses to the reviewer’s comments in the editor’s letter and revised manuscript**

**Comments to the Author**

**1**. If the authors have adequately addressed your comments raised in a previous round of review and you feel that this manuscript is now acceptable for publication, you may indicate that here to bypass the “Comments to the Author” section, enter your conflict-of-interest statement in the “Confidential to Editor” section, and submit your "Accept" recommendation.

Reviewer #5: All comments have been addressed

Reviewer #6: All comments have been addressed

Reviewer #7: All comments have been addressed

**2.** Is the manuscript technically sound, and do the data support the conclusions?

The manuscript must describe a technically sound piece of scientific research with data that supports the conclusions. Experiments must have been conducted rigorously, with appropriate controls, replication, and sample sizes. The conclusions must be drawn appropriately based on the data presented.

Reviewer #5: Yes

Reviewer #6: Partly

Reviewer #7: Yes

3. Has the statistical analysis been performed appropriately and rigorously?

Reviewer #5: Yes

Reviewer #6: No

Reviewer #7: Yes

4. Have the authors made all data underlying the findings in their manuscript fully available?

Reviewer #5: Yes

Reviewer #6: Yes

Reviewer #7: Yes

5. Is the manuscript presented in an intelligible fashion and written in standard English?

PLOS ONE does not copyedit accepted manuscripts, so the language in submitted articles must be clear, correct, and unambiguous. Any typographical or grammatical errors should be corrected at revision, so please note any specific errors here.

Reviewer #5: Yes

Reviewer #6: No

Reviewer #7: Yes

6. **Review Comments to the Author**

Please use the space provided to explain your answers to the questions above. You may also include additional comments for the author, including concerns about dual publication, research ethics, or publication ethics. (Please upload your review as an attachment if it exceeds 20,000 characters)

**Reviewer #5:**

**Comment 1**: Although the study addresses a local issue, the knowledge generated can be applied to other regions with similar characteristics. The analysis is robust and holds both local and global relevance. Additionally, the authors have successfully addressed the feedback from previous reviews. My only remaining suggestion is to polish the English phrasing.

**Response:** Thank you for helpful recommendation. We have accepted the feedback and tried to edit the text throughout the manuscript so that it is grammatically correct and professionally sound.

**Reviewer #6:**

The manuscript investigates the chemical composition and mosquito repellent efficacy of essential oils extracted from Croton macrostachyus, Echinops kebericho, Eucalyptus globulus, and Juniperus procera against laboratory-reared Anopheles arabiensis. The authors characterized the essential oils using GC-MS and evaluated their repellent activities through WHO-based arm-in-cage bioassays involving human volunteers. Effective doses (ED50 and ED99), complete protection time (CPT), and the efficacy of binary oil combinations were also assessed. The topic is relevant because vector behavioral changes and increasing insecticide resistance necessitate the development of alternative mosquito control strategies. Furthermore, the investigation of Ethiopian medicinal plants represents a valuable contribution to ethnopharmacology and botanical insecticide research. The manuscript contains interesting experimental data and addresses an important public health issue. Although the manuscript has been significantly improved following the authors' revisions, several important points still require further clarification in order to enhance its scientific quality. Several methodological, analytical, statistical, and interpretative weaknesses considerably reduce the scientific robustness of the study. In its present form, several conclusions are insufficiently supported by the data.
Overall, the manuscript has scientific merit but requires substantial revision before it can be considered for publication.

**Majors Comment
Comment 1**. Line 57, The Introduction mentions insecticide resistance but does not explain why repellents remain effective despite physiological resistance mechanisms. The authors should clearly distinguish between: insecticidal resistance, behavioral avoidance, host-seeking inhibition, repellency mechanisms. This distinction is important for readers unfamiliar with vector biology.

**Response:** We thank the reviewer for this insightful comment. A concise explanation has now been added to the introduction section **(Lines 54–57)** after the comment in the revised manuscript.

**Comment 2.** Lines 165-170, Only five volunteers participated in the bioassays. The authors state that this sample size provides sufficient statistical power. However, no sample size calculation or power analysis is presented. This claim should either be justified statistically or removed.

**Response:** We appreciate the reviewer for this critical comment. Several overstated statistical phrasings in the manuscript have been now revised to ensure accuracy in response to the feedback. Accordingly, we have removed the claim concerning statistical power. Instead, the text has now been revised to clarify that we utilized a repeated-measures design consisting of five participants (*n* = 5) evaluated across three independent replicates, yielding a total of 15 observational assessments **(Line 166-169)** in the revised manuscript. This approach aligns with a recent methodological study by Moreno-Gomez et al (2025), which investigated the sample size issue in arm-in-cage repellent screening. Their evaluation demonstrated that using repeated measure design (2 times repeated) with 5 subjects produced complete protection time estimate statistically equivalent to the 10-subject design (p > 0.05) due to comparable intra-individual and inter-individual variability. The design may optimize resource efficiency approach during the screening phase by taking the same person variability provides a statistically rigorous approach while responsibly minimizing human participant exposure. Similarly, the current preliminary screening test provides a strong foundation with recommendation for future validation study should employ larger sample size with statistical power calculation basis. We explicitly articulate this methodological justification in the revised manuscript. Additionally, we have made the findings or conclusions more cautious throughout the discussion section. The broader generalizations have now been reframed as localized laboratory insights, accurately aligning the conclusions with the screening scope. We have also included the study limitation to address the small sample sizes in this preliminary laboratory screenings.

**Comment 3**. Lines 187, 195-196, The cumulative dose procedure requires further explanation. Please clarify: why cumulative dosing was preferred, whether previous doses influenced subsequent responses, how evaporation between successive applications was controlled.

**Response:** We thank the reviewer for this methodological question. We have updated the materials and methods section **(Lines 193–201)** to clarify the details of the cumulative dosing procedure. Following WHO recommendations, cumulative dosing was used to determine a clear dose-response curve. To prevent evaporation effects, successive doses were applied at fixed time intervals. Evaporation was controlled by performing all tests under constant, controlled room conditions and volunteers were restricted inside the testing room for the entire duration of the experiment to ensure a stable physiological condition.

**Comment 4**. Lines 231-238, The authors correlate the abundance of individual compounds with repellent activity. This approach is problematic because essential oils are complex multicomponent mixtures exhibiting numerous synergistic and antagonistic interactions. Correlation alone cannot establish that individual compounds are responsible for biological activity. The Discussion should acknowledge this limitation.

Response: Thank you for this critical comment. We accepted the comment and removed any texts associating individual compounds with the observed repellency, following the feedback.

**Comment 5.** Lines 267-268, Statements such as « Both compounds are known to have insect repellent properties » are too strong because these compounds were not individually tested in this study. The wording should be changed to « may contribute » or « have previously been reported » instead of implying causality.

**Response:** Thank you for the suggestion. We have corrected the text as suggested to ensure the claim remains cautious and scientifically accurate in the discussion section. Specifically, we removed the interpretation text from the results section and repositioned it within the discussion section in, where it can be contextualized more appropriately in the revised manuscript.

**Comment 6.** Lines 248-274, The GC-MS identification relies exclusively on NIST and Wiley spectral libraries. No Linear Retention Index (LRI) or Kovats Retention Index (RI) values are reported. For essential oil characterization, spectral matching alone is generally insufficient for reliable compound identification. The authors should report experimental retention indices together with literature values whenever possible.

**Response:** We thank for the critical reviewer’s concern. We have re-evaluated our GC-MS raw data and calculated the experimental Retention Index (RI) values for the identified chemical compounds using a standard series of n-alkanes run under the same chromatographic conditions. We have also revised Tables [2-5] to include two separate columns presenting literature retention index (RI_Lit_) and experimental retention index (RI_Exp_) in response to the feedback.

**Comment 7.** Table 2, Several reported compounds appear unusual or unexpected in essential oils, including steroid-like molecules such as Pregn-16-en-20-one and Gestonorone derivatives. These identifications should be carefully verified because they may represent false-positive library matches.

**Response:** Thank you for the valuable observation. We have carefully re-examined the entire chemical profile. All redundant entries, as well as unusual or unexpected compounds including the mentioned have been removed. The finalized chemical compound list (tables 2-5) has been thoroughly verified against standard libraries to ensure accuracy and remove any false-positive matches in response to the feedback.

**Comment 8**. Table 3, Line 286, Only ED50 and ED99 are reported. The probit regression parameters should also include: Regression slope, Confidence limits, and χ² values with interpretation.

**Response:** We thank and agree with the suggestion that reporting the full set of probit regression parameters is essential to establish the mathematical validity and goodness-of-fit of our dose-response curves. We have thoroughly updated our results section and added a two new separate column (Table 6) presenting the regression slopes and Wald Z-statistic with p-value.

**Comment 9**. The manuscript does not sufficiently explain why binary combinations of essential oils were investigated. The hypothesis that blending oils may improve efficacy should be supported by previous literature.

**Response:** Thank you for the critical observation. The rationale for using blends and the hypothesis that blending essential oils may enhance efficacy based on synergistic interactions. We have revised the introduction and discussion section to clarify this hypothesis and support with literature **(line 80-81)**.

**Comment 10**. The negative control consists of ethanol. Please justify why ethanol was selected instead of another carrier commonly used in topical repellent formulations.

**Response:** Thank you. Ethanol was selected as the negative control in accordance with the WHO guidelines because ethanol evaporates completely and rapidly from the skin allowing for an accurate measurement of baseline mosquito attraction.

**Comment 11**. Dose Selection, the rationale for selecting concentrations of 10 % and 20 % should be explained. Were these concentrations based on previous studies or preliminary experiments?

**Response:** We thank for this constructive comment. We have updated the materials and methods section in the revised manuscript **(Lines 207–212)** to clarify our rationale for dose selection.

The selection of the 10 % and 20 % concentrations for the essential oils was based on preliminary dose-response testing, established literature protocols and WHO guideline. Our preliminary dose response study confirmed that the 10 % concentration is slightly higher than the calculated effective dose threshold (ED₉₉) for all tested essential oils, ensuring robust baseline activity while matching commonly evaluated doses across peer-reviewed botanical repellent literature. Additionally, a 20 % dosage was selected in accordance with standardized WHO guidelines, allowing for a direct, standardized comparison against DEET (20 %), which was employed as conventional positive control for mosquito repellency.

**Comment 12**. Binary Mixtures, Authors evaluates binary oil combinations. Please specify: mixing ratio; preparation procedure; stability before testing; possible phase separation.

**Response:** We appreciate the reviewer for the comment. The binary oil combinations were prepared by mixing the individual essential oils in a 1:1 (v/v) ratio using micropipettes, followed by shaking to ensure complete homogeneity. These formulations were prepared fresh on the day of testing, stored at room temperature (27 ± 2 °C). Throughout this time, the mixtures remained completely miscible and stable, with no visible phase separation, observed either during blending or post-dilution in the ethanol. These changes following the feedback has been indicated in the revised manuscript **(line 151-157).**

**Comment 13**. Figures 1–3, The figures present only mean repellency percentages. Please include: error bars, confidence intervals, statistical annotations.

**Response:** Thank you. We have accepted the comment and included error bars in the percentage repellency trend graphs (Figures 1-3). However, to maintain figure readability and avoid text redundancy, complete median protection times, ranges, and post-hoc statistical annotations are presented in Tables 7-9.

**Comment 14**. The Discussion should acknowledge that laboratory bioassays may not accurately predict field performance because of: wind; temperature; behavioral variability.

**Response:** Thank you. We have updated the discussion section in response to the feedback to explicitly acknowledge that laboratory bioassays may not fully predict field performance due to environmental factors such as wind, temperature fluctuations, and natural mosquito behavioral variability (Lines 425-427) in the revised manuscript.

**Minors Comment**

**Comment 15**. Several abbreviations are introduced before being defined.

**Response:** We thank the reviewer for identifying this formatting error. We have reviewed the entire manuscript to ensure that every abbreviation is explicitly defined.

**Comment 16**. Scientific names should be consistently italicized in all figure legends.

**Response:** We thank the reviewer. We have reviewed all figure legends and ensured that all scientific names are consistently italicized throughout the manuscript.

**Comment 17**. Several tables would benefit from reporting sample size (n).

**Response:** We thank the reviewer for this constructive suggestion. We have updated all tables to explicitly report the sample size (*n* = 5 subjects).

**Comment 18**. Please verify spacing before and after percentage symbols and units. Write 10 % not 10%.

**Response:** Thank you. The texts have been revised to include a space between numerical values and the percentage symbol after the comment

**Comment 19**. The references should be checked for consistency according to the PLOS ONE citation style.

**Response:** We thank the reviewer for this note. The entire bibliography and all in-text citations have been checked to ensure complete consistency with the PLOS ONE style.

**Comment 20**. Finally, the manuscript would benefit from careful language editing to improve clarity and correct minor grammatical and formatting inconsistencies.

**Response:** We thank the reviewer for this constructive comment. A line-by-line language edit of the entire manuscript have been made to improve overall clarity, sentence structures, as well as to correct grammatical errors. Furthermore, all formatting parameters, including unit spacing and definitions of abbreviation have been checked and corrected to ensure the manuscript meets scientific standard.

**Reviewer #7:** Here are my comments to the authors.
**Title**
**Comment 1**. -What is the importance of showing **complex matrices** on a title? Why not “**Chemical composition and repellency of essential oils from four medicinal plants against Anopheles arabiensis (Diptera: Culicidae) under laboratory conditions**"?

**Response:**

Thank you for the suggestion. We accept the comment and made changes on the title as suggested. Additionally, we have rearranged the title to place “repellency**”** first to better reflect the primary focus of our study. The phrase "complex matrices" was added to the title during the previous round of review based on reviewer feedback. However, in light of the current suggestion to remove it, we removed the phrase from the title to keep it concise, but have repositioned it to the abstract section **(Line 17)** to preserve the context.

**Abstract**

**Comment 2**. The abstract section should have to rewritten in a well-structured manner.

**Response:** We thank for the constrictive feedback. The abstract section has been rewritten to ensure a clear, logical, and well-structured flow after the comment.

**Comment 3**. Lines 14 to 16 can be better rewritten by merging into 1 or 2 lines with a nice flow

**Response:** Thank you. The text has been revised following the comment.

**Comment 4**. Lines 16 to 17 mixed objective and method; it's better to rewrite a clear objective using more appropriate words and the method followed separately.

**Response:** Thank you for the comment. We have revised the statement in the revised manuscript to first state objective, followed by methods.

**Comment 5**. Line 17 against “laboratory-reared”: What is the importance of explaining it under the objective? Better to connect with the sentence under line 23.

**Response:** We appreciate the comment. The term "laboratory-reared" has been removed from the objective sentence and integrated into the methodology text after the comment.

**Comment 6**. Lines 18 to 21: It's better to begin with "Essential oils were extracted from fresh parts of ------ using hydrodistillation."

**Response:** We thank for this note. We have revised the sentence as suggested in the revised version of the manuscript.

**Comment 7**. Line 22, “chemical composition of the EOs was characterized by GC-MS." Is "characterized" appropriate? Or analyzed using GC-MS?

**Response:** Thank you for the comment. We agree that "analyzed using GC-MS" is a more appropriate.

**Comment 8**. Lines 23 to 25 should have to be rewritten in a clear manner by combining separated phrases.

**Response:** Lines 23 to 25 have been rewritten by combining separated phrases as suggested.

**Comment 9**. Lines 25 to 28, the authors suggested rewriting the sentences by avoiding unnecessary words

**Response:** We thank for the comment. We have updated the text in response to the comment.

**Comment 10**. For example, about 162 compounds were identified, among which are the most abundant and showed a positive correlation ---

**Response:** Thank you. We have rephrased the statement to clarify the result.

**Comment 11**. Line 28, the plant parts used for EOs extraction should be stated as the method section; otherwise, it makes a confusion

**Response:** We thank you and the text has been updated in response to the comment.

**Comment 12**. Line 30: Similarly, it is better to explain “binary blends” as treatments using few words to avoid confusion.

**Response:** Thank you. The text has been revised concisely, avoiding any potential confusion.

**Introduction**

**Comment 13**. Lines 38 to 42 have too much introduction and would be better if the authors rewrite the paragraph focusing on the direct study subject.

**Response:** Thank you for the feedback. We have thoroughly revised the paragraph in the revised manuscript to remove the extra background information.

**Comment 14**. It is recommended to rewrite the incomplete phrases with too much in-text citation.

**Response:** Thank you for the feedback. The incomplete phrases have been rewritten in the revised manuscript.

**Comment 15**. The introduction section is recommended to improve its connectivity and flow of ideas between sentences as well as between paragraphs.

**Response:** We thank the reviewer for this suggestion. The introduction section has been re-visited and revised in response to the comment.

**Comment 16**. Lines 40 to 42 can be summarized in one line by combining the ideas. For example, line 43 would be better if it begins with “Malaris is transmitted by over 70 Anopheles species." ---

**Response:** We thank the reviewer and have updated the text in response to the comment.

**Comment 17**. Lines 61 and 62: Is it economical to use EOs in this regard?

**Response:** Thank you. The text has been revised to avoid confusion following the comment.

**Comment 18**. Line 66: Why can't the authors use the form of smoke and tars instead of EOs?

**Response:** Thank you for the suggestion. For the current study, we focused specifically on essential oils to establish a standardized baseline of their volatile chemical components and evaluate their direct repellent potency under controlled conditions without the confounding variables of combustion. Testing these plants in the form of smoke or tars is a valuable and we have noted as part of our future research plans.

**Comment 19**. Lack of literature review regarding the studied plant essential oils' repellency effects and existing research gaps.

**Response:** Thank you. We have expanded the introduction section to include a thorough literature review of repellency effects of the studied plant essential oils.

**Comment 20**. At the end of Line 68, is “both safe and biodegradable” not clear? Biodegradable for EOs?

**Response:** The text has been revised in the revised manuscript following the comment.

**Comment 21**. Lines 71 and 72, vast literature review: It is better to focus on those plants used in the current study with application method and repellent efficacy test

**Response:** Thank you for the comment. We have revised the introduction section as suggested.

**Materials and Methods
Comment 22**. It is better to write the “Essential oil extraction from plant parts” section by avoiding mixed procedures and some extra words.

**Response:** Thank you. The text has been updated in response to the feedback.

**Comment 23**. Would it be better if the authors inserted the image of plants used in this study?

**Response:** We appreciate the suggestion. Due to seasonal and local limitations in obtaining publication-grade field photographs, we have instead a detailed taxonomic and geographic profile in Table 1.

**Comment 24**. Line 110, for how long are the EOs stored before repellency tests? It is better to state the exact duration, as some active components escape due to the volatile nature of EOs.

**Response:** Thank you for the critical comment. The essential oils were evaluated within one to three weeks of extraction. We have updated Line 110 of the revised manuscript to state this exact duration and storage configuration.

**Comment 25**. Line 112, “Gas chromatography/mass spectrometry (GC/MS) analysis”??? Instead, essential oil composition analysis, GC-MS profile of essential oils, or chemical composition of essential oils.

**Response:** Thank you. Line 112 has now been updated as GC-MS profile of essential oils in response to the feedback.

**Comment 26**. Lines 113 to 134, Try to present only the main steps and remove extra words.

**Response:** Thank you. We have revised Lines 113 to 134 to remove redundant phrasing and extra words in the revised manuscript following the comment.

**Comment 27**. Lines 145 to 148 are not important to explain multiple times once the extracted EOs were already known. It is enough to write as “The repellency test was performed following WHO guidelines" (41).

**Response:** Thank you for the comment. We have revised the text as suggested.

**Comment 28**. Lines 150 to 153: try to minimize repeated similar words, which seem boring, and rewrite in clear and attractive manner.

**Response:** We appreciate the suggestion. Lines 150 to 153 have been revised to enhance sentence structure, ensuring a clear and concise presentation.

**Comment 29**. For example, five various concentrations of EOs (1%---10%) were prepared in ethanol (v/v) or µL/mL—instead of the word "per."

**Response:** We thank for this suggestion. We have revised Lines 150 to 153 to improve sentence flow.

**Comment 30**. Line 154, “Additionally, a total of six binary combinations of the essential oils were created." "Formulated" can be used instead of "created."

**Response:** We thank the reviewer for this suggestion. We have revised the text in Line 154 to replace the word created with formulated.

**Comment 31**. Line 154 to 159 can be combined and presented together.

**Response:** Thank you. Lines 154 to 159 have been combined to improve readability following the feedback.

**Comment 32**. Line 160 to 1622 can be written in one line by connecting unnecessarily separated phrases.

**Response:** We thank the reviewer for this helpful suggestion. We have revised the text to remove the unnecessary phrasal separations and combined into a single, sentence to improve clarity and reading flow.

**Comment 33**. For repellency evaluation tests, it could be better if the authors include the image of the arm in the cage showing mosquito landing status/rate.

**Response:** We thank the reviewer for this constructive suggestion. While a live photograph of the assay captures the experimental setup, obtaining a publication-grade image that clearly displays individual mosquito landing rates inside a mesh cage can be visually challenging. To ensure optimal clarity, we have instead provided a detailed, step-by-step text description.

**Comment 34**. What is the application method of essential oils on the hands of volunteers? Spraying/painting?

**Response:** Thank you. The essential oils were applied to the skin using a pipetting and painting method to ensure precise dosage control. Utilizing micropipette to dispense the formulation followed by manual spreading ensures dosage accuracy over skin.

**Comment 35**. The authors explain that the testing duration lasted for 6 hours per person, and the experiments have been conducted in three replications. Does it mean one volunteer waited for 18 hours for one treatment?

Response: We thank the reviewer for allowing us to clarify the experimental timeline. A single volunteer did not wait for 18 hours continuously. The 6-hour evaluation represents the maximum observation window for a single treatment session on a given day. The three independent replications were conducted on separate days, with a minimum washout period of 7 days between sessions. This setup was deliberately chosen to prevent volunteer fatigue, ensure ethical compliance, and allow the skin surface to return to its baseline physiological state before subsequent evaluations. We have explicitly clarified this protocol in the revised manuscript **(line 224-227)**

**Statistical analysis
Comment 36**. This section is too much and mixed with calculation of some parameters; it is recommended that the authors clearly present only statistical analysis (design, testing, etc.).

**Response:** We thank the reviewer for this constructive suggestion. We have now restructured to separate parameter calculations from formal statistical analysis in the revised manuscript.

**Results**
**Comment 37**. Essential oil yield or essential oil content? Which is more appropriate in this context?

**Response:** The phrasing has now been updated in the extraction data table (Table 1) to use "Essential oil yield (%) instead of content following the suggestion.

**Comment 38**. Line 246, Table caption should be written correctly. For instance, Table 1. Essential oil yield (%) of five medicinal plants collected from the Gibe Valley, Ethiopia (and others better in self-explanatory ways).

**Response:** Thank you. We have reviewed and updated the captions for all tables throughout the revised manuscript to ensure they are fully self-explanatory in response to the comment.

**Comment 39**. It's better to present table with the top and bottom border only.

**Response:** We appreciate the reviewer for this formatting suggestion. We have updated all tables across the revised manuscript following the feedback.

**Comment 40.** Lines 249 to 251 could be better written in short by removing long words.

**Response:** Lines 249 to 251 have been revised to remove redundant and lengthy phrasing.

**Comment 41.** A total of 162 compounds in which essential oils?

**Response:** We have revised the text to explicitly state that this number represents the collective chemical profile across all four studied plant species in the revised manuscript.

**Comment 42.** The author can be designate or rename if required to better present the results of each essential oil extracted from different plants.

**Response:** Thank you for this comment. To improve clarity, we have standardized the names of the essential oils throughout the revised manuscript by using their botanical designations in response to the comment.

**Comment 43.** Lines 255 and 256: “All three of these most abundant chemical compounds derived from E. globulus have been known for insect-repellent properties." Is it important to present it here?

**Response:** Thank you. We fully agree with the reviewer. This interpretive statement has now been removed to ensure the text remains focused on our descriptive data.

**Comment 44.** Lines 257 to 258: The correlation coefficient value presented does not clearly show the association between which of the two compounds?

**Response:** We thank the reviewer for highlighting this ambiguity. Given the complex chemical matrices inherent to the essential oils, we determined that standard linear correlation analysis could yield conceptually misleading concepts. Because the overall insect repellency is driven by intricate multi-component interactions rather than simple isolated dual-compound pairings, we have completely removed the correlation analysis and its corresponding data from the entire manuscript to ensure strict scientific accuracy and data integrity following the comments.

**Comment 45.** Lines 262 to 263, the author better justify the finding with appropriate citation for such sentences “These 262 compounds are sesquiterpenes, and there are indications that some sesquiterpenes can exhibit repellent properties against mosquitoes and other insects," and the same is true for the sentences in lines 267 and 268 and lines 273 and 274.

**Response:** Thank you. We fully agree with the comment. This interpretive statement has now been removed from the result section to ensure the text remains focused on our descriptive data.

**Comment 46.** Line 275, Table 2. It would be better if the authors could present essential oil composition for each plant separately using an appropriate standard table format with a clear caption for each.

**Response:** We thank and fully agree with the recommendation. We have restructured the results by separating the table 2 in to 4 tables for each studied plant species in response to the comment.

**Comment 47.** Line 278: It's better to start with a clear presentation of results.

**Response:** We thank the reviewer for this constructive suggestion. We have revised the text starting at Line 278 to remove introductory phrasing and started with a clear, direct presentation of the results following the feedback.

**Comment 48.** Why do not the authors separately present the correlation analysis using a table rather than attaching raw data as supplementary information?

**Response:** We thank for this recommendation. We have already removed the correlation analysis from the entire manuscript and the supplementary information as we determined that standard linear correlation analysis could yield conceptually misleading concepts. Because the overall insect repellency is driven by intricate multi-component interactions rather than simple isolated dual-compound pairings.

**Comment 49.** Why is the figure not inserted at its appropriate places with their corresponding caption.

**Response:** Thank you. We understand the reviewer's concern. However, in accordance with the PLOS ONE submission guidelines, figures must not be embedded within the main manuscript text. Instead, they must be uploaded as separate individual files. The figure caption has been included in the text at their appropriate locations.

**Comment 50.** In Tables 4, 5, and 6, it could be better if the authors presented the mean ± standard deviation or standard error for values labeled with significant mean letters.

**Response:** We appreciate the suggestion. However, because our datasets did not follow a normal distribution, we utilized non-parametric analyses (Kruskal-Wallis and Dunn's post-hoc tests) to evaluate statistical significance. In non-parametric statistics, presenting the median with the interquartile range is the standard and mathematically correct method for descriptive reporting, instead of the mean ± standard deviation. To clarify this for the readers, we have explicitly updated the captions and footnotes of these Tables to reflect all values represent the median with interquartile range.

**Comment 51.** What is the main objective of using blended essential oils, and what is the basis for blending proportions?

**Response:** The purpose of blending oils was to produce synergistic interactions to enhance prolong protection duration. The 1:1 (v/v) proportion serves as a standardized, balanced baseline to ensure an equal concentration of each oil, providing a uniform component to evaluate their combined repellent effect.

**Discussion**
**Comment 52.** Lines 356 to 358, The authors should focus on similar parameters and extraction methods employed from the previous study for discussion.

**Response:** We thank and accept this suggestion. The discussion section has now been revisited and revised following the comment.

**Comment 53.** It could be better if the authors briefly explain the testing method and conditions of those plants studied for repellent properties in previous studies.

**Response:** Thank you. The discussion section has now been updated in response to the suggestion to reflect contextual comparison of the current screening results with previous findings, in the revised manuscript.

**Comment 54.** Lines 389 to 390: How can the type of solvent affect the efficiency of essential oils since there is no further extraction?

**Response:** Thank you. We appreciate this comment. The text has been revised in response to the comment to avoid any confusion in the revised manuscript.

**Comment 55.** Lines 390 to 394: It's better to rewrite by avoiding some repetition of similar and extra words with better flow.

**Response:** We thank for this recommendation. We have rewritten the texts to remove redundant words and extra phrasing in the revised manuscript following the feedback.

**Comment 56.** Lines 401 to 403: The authors have to clarify the drawback of using concentrated essential oils.

**Response:** We thank for this comment. The text has been revised after the comment to explicitly clarify the main drawbacks of using concentrated essential oils, due to potential risk of skin irritation or dermal toxicity at high concentrations in the revised manuscript.

**Comment 57.** Lines 427 to 429: Rewrite in better ways by aligning separated sentences and use one citation (61).

**Response:** We thank the reviewer for this suggestion. We have rewritten Lines 427 to 429 by connecting the unnecessarily separated sentences into a single statement that flows smoothly in response to the comment.

**Comment 58.** Line 434, "A comparable result was reported by previous studies on essential oil of E. globulus in Ethiopia." What are the concentration and testing method/conditions?

**Response:** We thank the reviewer for this question. We have updated the justification in response to the comment to provide a precise comparison with our current findings in the revised manuscript.

**Comment 59.** Lines 442 (end) to 445 contain similar ideas and are better revised on the basis of blending importance and consequences.

**Response:** We thank the reviewer for this constructive suggestion. We have revised the text to remove overlapping ideas in the revised manuscript.

**Comment 60.** Lines 452 to 453, “Therefore, the result implies that a 1:1 combination of most of the essential oils tested in this study did not cause a synergistic or antagonistic reaction." Is this enough evidence to conclude?

**Response:** We thank the reviewer for this critical observation. To address this comment, we have revised the text to change the phrasing in order to avoid overstated conclusions in the revised manuscript in response to the comment.

**Comment 61.** Lines 457 to 465: The authors tried to elaborate on the use of essential oils being less effective compared to commercial repellents in many aspects. So, what is the importance of using essential oils, particularly in terms of affordability, applicability, safety, and economic feasibility?

**Response:** Thank you for this constructive comment. We agree that the broader practical and socio-economic advantages of these essential oils from local plants should be highlighted. Therefore, we have tried to clarify that these locally available plant extracts provide a safe, affordable, and practical alternative for mosquito control because the plants grow locally and are easy for people to use following the feedback.

**Limitation of study
Comment 62.** What about the application method of essential oils (spray or other repellency testing methods (if any)?

**Response:** We thank the reviewer for highlighting this important point. We acknowledge that manual painting method used during this preliminary screening phase does not capture the spray dynamics of commercial consumer applications.

**Comment 63.** Advanced essential oil formulation (macro and nano encapsulation) for better applicability?

**Response:** Thank you. We accept the suggestion and have updated the limitation of the study section to explicitly recommend micro- and nano-encapsulation technologies as necessary strategies to regulate essential oil volatility and extend protection times for practical consumer application in the revised manuscript.

**Comment 64.** Economic feasibility analysis?

**Response:** We thank and acknowledge this limitation in response to the feedback in the revised manuscript.

**Conclusion**
**Comment 65.** The conclusion is full of recommendation and can be better improved.

**Response:** We thank the reviewer for this comment. The conclusion section has been revised to focus on the core findings in response to the comment.
