# Supplementary material for: Repellency and chemical composition of essential oils from four medicinal plants against Anopheles arabiensis (Diptera: Culicidae) under laboratory conditions
Source: PLoS One. 2026 Sep 15;21(9):e0358040. doi: 10.1371/journal.pone.0358040 (PMC13577348; doi:10.1371/journal.pone.0358040)
Supplement: Peer review history

# Peer review: Decision Letter 0

**DOI:** 10.1371/journal.pone.0358040.r001

**Authors:** Mansureh Ghavam

21 Apr 2026

Dear Dr. Teshome,

Thank you for submitting your manuscript to PLOS ONE. After careful consideration, we feel that it has merit but does not fully meet PLOS ONE’s publication criteria as it currently stands. Therefore, we invite you to submit a revised version of the manuscript that addresses the points raised during the review process.

Please submit your revised manuscript by Jun 05 2026 11:59PM. If you will need more time than this to complete your revisions, please reply to this message or contact the journal office at plosone@plos.org. When you're ready to submit your revision, log on to https://www.editorialmanager.com/pone/ and select the 'Submissions Needing Revision' folder to locate your manuscript file.

- A letter that responds to each point raised by the academic editor and reviewer(s). You should upload this letter as a separate file labeled 'Response to Reviewers'.
- A marked-up copy of your manuscript that highlights changes made to the original version. You should upload this as a separate file labeled 'Revised Manuscript with Track Changes'.
- An unmarked version of your revised paper without tracked changes. You should upload this as a separate file labeled 'Manuscript'.

If you would like to make changes to your financial disclosure, please include your updated statement in your cover letter. Guidelines for resubmitting your figure files are available below the reviewer comments at the end of this letter.

If applicable, we recommend that you deposit your laboratory protocols in protocols.io to enhance the reproducibility of your results. Protocols.io assigns your protocol its own identifier (DOI) so that it can be cited independently in the future. For instructions see: https://journals.plos.org/plosone/s/submission-guidelines#loc-laboratory-protocols. Additionally, PLOS ONE offers an option for publishing peer-reviewed Lab Protocol articles, which describe protocols hosted on protocols.io. Read more information on sharing protocols at https://plos.org/protocols?utm_medium=editorial-email&utm_source=authorletters&utm_campaign=protocols.

As the corresponding author, your ORCID iD is verified in the submission system and will appear in the published article. PLOS supports the use of ORCID, and we encourage all coauthors to register for an ORCID iD and use it as well. Please encourage your coauthors to verify their ORCID iD within the submission system before final acceptance, as unverified ORCID iDs will not appear in the published article. *Only*  the individual author can complete the verification step; PLOS staff *cannot*  verify ORCID iDs on behalf of authors.

We look forward to receiving your revised manuscript.

Kind regards,

Mansureh Ghavam

Academic Editor

PLOS One

Journal Requirements:

When submitting your revision, we need you to address these additional requirements.

1. Please ensure that your manuscript meets PLOS ONE's style requirements, including those for file naming. The PLOS ONE style templates can be found at

https://journals.plos.org/plosone/s/file?id=wjVg/PLOSOne_formatting_sample_main_body.pdf and

https://journals.plos.org/plosone/s/file?id=ba62/PLOSOne_formatting_sample_title_authors_affiliations.pdf

2. In your Methods section, please provide additional information regarding the permits you obtained for the work. Please ensure you have included the full name of the authority that approved the field site access and, if no permits were required, a brief statement explaining why.

3. Thank you for stating the following in the Acknowledgments Section of your manuscript:

“This study was financed by the Office of the Vice President for Research and Innovation, Addis Ababa University (grant number RD/PY-524/2023).”

We note that you have provided funding information that is not currently declared in your Funding Statement. However, funding information should not appear in the Acknowledgments section or other areas of your manuscript. We will only publish funding information present in the Funding Statement section of the online submission form.

Please remove any funding-related text from the manuscript and let us know how you would like to update your Funding Statement. Currently, your Funding Statement reads as follows:

“This study was financed by the Office of the Vice President for Research and Innovation, Addis Ababa University (grant number RD/PY-524/2023).”

Please include your amended statements within your cover letter; we will change the online submission form on your behalf.

4. Thank you for stating the following financial disclosure:

“This study was financed by the Office of the Vice President for Research and Innovation, Addis Ababa University (grant number RD/PY-524/2023).”

Please state what role the funders took in the study.  If the funders had no role, please state: "The funders had no role in study design, data collection and analysis, decision to publish, or preparation of the manuscript."

If this statement is not correct you must amend it as needed.

Please include this amended Role of Funder statement in your cover letter; we will change the online submission form on your behalf.

5. Thank you for stating the following financial disclosure:

“This study was financed by the Office of the Vice President for Research and Innovation, Addis Ababa University (grant number RD/PY-524/2023).”

We note that one or more of the authors is affiliated with the funding organization, indicating the funder may have had some role in the design, data collection, analysis or preparation of your manuscript for publication; in other words, the funder played an indirect role through the participation of the co-authors. If the funding organization did not play a role in the study design, data collection and analysis, decision to publish, or preparation of the manuscript and only provided financial support in the form of authors' salaries and/or research materials, please do the following:

1. Review your statements relating to the author contributions, and ensure you have specifically and accurately indicated the role(s) that these authors had in your study. These amendments should be made in the online form.

2. Confirm in your cover letter that you agree with the following statement, and we will change the online submission form on your behalf:

“The funder provided support in the form of salaries for authors [insert relevant initials], but did not have any additional role in the study design, data collection and analysis, decision to publish, or preparation of the manuscript. The specific roles of these authors are articulated in the ‘author contributions’ section.

6. If the reviewer comments include a recommendation to cite specific previously published works, please review and evaluate these publications to determine whether they are relevant and should be cited. There is no requirement to cite these works unless the editor has indicated otherwise.

Additional Editor Comments:

Authors are requested to consider all the comments of the reviewers and prepare a point-by-point response. In addition, the following should be noted:

Clarify the doses applied in the first series of experiments and how the results of this bioassay were used in the second experiment for the duration of protection.

The repellent properties of any of the compounds identified in the essential oils should be presented. The correlation between the chemical component and the mosquito repellent effect was not investigated.

Finally, a data analysis should be performed to determine the potential synergistic effects of the essential oils.

It is also recommended that the writing be professionally improved in terms of English and nativeness.

[Note: HTML markup is below. Please do not edit.]

Reviewers' comments:

Reviewer's Responses to Questions

**Comments to the Author**

1. Is the manuscript technically sound, and do the data support the conclusions?

Reviewer #1: Yes

Reviewer #2: Partly

**********

2. Has the statistical analysis been performed appropriately and rigorously?

Reviewer #1: Yes

Reviewer #2: Yes

**********

3. Have the authors made all data underlying the findings in their manuscript fully available?

The PLOS Data policy

Reviewer #1: Yes

Reviewer #2: Yes

**********

4. Is the manuscript presented in an intelligible fashion and written in standard English?

Reviewer #1: No

Reviewer #2: Yes

**********

Reviewer #1: The authors investigated the mosquito repellent effects of five essential oils that extracted from Croton macrostachyus, Echinops Kebericho, Eucalyptus globuluns, and Juniperus procera against Anopheles arabiensis. The experimental results showed that all the five essential oils exibit significant repellent properties. However, only the leaf and seed oils from C. macrostachyus meet the criteria by Environmental Protection Agency's registration. This research is interesting to the readerships of the PLoS One. Some specific comments are as follows:

1.The English writing need to be improved.

2.The chemical component of the five essential oils were analysed based on GC-MS. However, the correlations between the chemical component and the mosquito repellent effect were not investigated.

3.Rewrite the abstract.

4.Vector graphics should be provided in Figures 1 to 3.

5.Revise the unit symbol of temperature to ℃.

6.Use abbreviation for Latin names of the species after they are full named in the manuscript.

Reviewer #2: Dear authors,

This paper deals with the determination of repellent properties of essential oils from 5 plant species against Anopheles arabiensis.

Herein you will find my comments for your consideration.

Line 59: Do EOS really disrupt natural ecosystems? Please clarify how. The induction of insecticide resistance is not relevant for repellents, so please remove it. Here it would be better to find some references for the potential of negative effects of synthetic repellents to humans.

Line 73: use “repellent” not “repulsive”. Also, in the scope you have to mention the tested plant species.

Lines 160-169: The applied doses should be clarified. It seems that you applied one dose of each essential oil in μL per cm2. Is the range 0.86-0.98 μL referring to the amount of applied EO per cm2 due to differences in the skin area among the volunteers?

What was the time interval between the application of cumulative doses? This may have affected the repellent potency due to evaporation. Hence, the repellent result may be different between one high dose and cumulative low doses that give the same high dose.

The exposure time of treated arm to mosquitoes for the estimation of effective dose is not clarified.

Why did you use 100 mosquitoes for the estimation of effective dose and 200 mosquitoes for the duration of protection?

Line 187: In the statistical analysis the post hoc test should be clarified.

A major limitation of this study is that no bioassay was conducted with the major compounds of the oils to identify potent repellent chemicals against mosquitoes and explain the action of the essential oil.

Table 3: The expression of the dose rate in μL/cm2 should be provided in the legend. Please confirm if this is the correct dose rate expression.

Table 4 & 5. Remove the columns with P<0.001. The reference in the main text is sufficient. The applied concentrations should be clarified in the legends of the tables.

For me it is not clear how the results from the first trial for the estimation of effective dose are correlated with the next trial for the estimation of protection time in terms of applied doses. Normally, the most potent doses from the first trial should have been used for the protection time assays. However, for the protection time you tested 10% and 20% concentrations for all EOs no matter what the ED99 was estimated from the first bioassay.

Lines 356-358: You mention the comparison between single and blended EOs, but no statistical comparison is made in this perspective. I would suggest elaborating more on your data by comparing the single and blended EOs to identify potential synergistic effects and support the relevant discussion.

**********

what does this mean?). If published, this will include your full peer review and any attached files.

If you choose “no”, your identity will remain anonymous but your review may still be made public.

**Do you want your identity to be public for this peer review?**  For information about this choice, including consent withdrawal, please see our Privacy Policy

Reviewer #1: No

Reviewer #2: No

**********

[NOTE: If reviewer comments were submitted as an attachment file, they will be attached to this email and accessible via the submission site. Please log into your account, locate the manuscript record, and check for the action link "View Attachments". If this link does not appear, there are no attachment files.]

To ensure your figures meet our technical requirements, please review our figure guidelines: https://journals.plos.org/plosone/s/figures

You may also use PLOS’s free figure tool, NAAS, to help you prepare publication quality figures: https://journals.plos.org/plosone/s/figures#loc-tools-for-figure-preparation.

NAAS will assess whether your figures meet our technical requirements by comparing each figure against our figure specifications.

# Author comment: Author response to Decision Letter 1

**DOI:** 10.1371/journal.pone.0358040.r002

20 May 2026

Please see attached response to reviewers file

Attachment Submitted filename: Response to Reviewers.docx

# Peer review: Decision Letter 1

**DOI:** 10.1371/journal.pone.0358040.r003

**Authors:** Mansureh Ghavam

24 Jun 2026

Dear Dr. Teshome,

Thank you for submitting your manuscript to PLOS ONE. After careful consideration, we feel that it has merit but does not fully meet PLOS ONE’s publication criteria as it currently stands. Therefore, we invite you to submit a revised version of the manuscript that addresses the points raised during the review process.

Please submit your revised manuscript by Aug 08 2026 11:59PM. If you will need more time than this to complete your revisions, please reply to this message or contact the journal office at plosone@plos.org. When you're ready to submit your revision, log on to https://www.editorialmanager.com/pone/ and select the 'Submissions Needing Revision' folder to locate your manuscript file.

- A letter that responds to each point raised by the academic editor and reviewer(s). You should upload this letter as a separate file labeled 'Response to Reviewers'.
- A marked-up copy of your manuscript that highlights changes made to the original version. You should upload this as a separate file labeled 'Revised Manuscript with Track Changes'.
- An unmarked version of your revised paper without tracked changes. You should upload this as a separate file labeled 'Manuscript'.

If you would like to make changes to your financial disclosure, please include your updated statement in your cover letter. Guidelines for resubmitting your figure files are available below the reviewer comments at the end of this letter.

If applicable, we recommend that you deposit your laboratory protocols in protocols.io to enhance the reproducibility of your results. Protocols.io assigns your protocol its own identifier (DOI) so that it can be cited independently in the future. For instructions see: https://journals.plos.org/plosone/s/submission-guidelines#loc-laboratory-protocols. Additionally, PLOS ONE offers an option for publishing peer-reviewed Lab Protocol articles, which describe protocols hosted on protocols.io. Read more information on sharing protocols at https://plos.org/protocols?utm_medium=editorial-email&utm_source=authorletters&utm_campaign=protocols.

As the corresponding author, your ORCID iD is verified in the submission system and will appear in the published article. PLOS supports the use of ORCID, and we encourage all coauthors to register for an ORCID iD and use it as well. Please encourage your coauthors to verify their ORCID iD within the submission system before final acceptance, as unverified ORCID iDs will not appear in the published article. *Only*  the individual author can complete the verification step; PLOS staff *cannot*  verify ORCID iDs on behalf of authors.

We look forward to receiving your revised manuscript.

Kind regards,

Mansureh Ghavam

Academic Editor

PLOS One

Journal Requirements:

If the reviewer comments include a recommendation to cite specific previously published works, please review and evaluate these publications to determine whether they are relevant and should be cited. There is no requirement to cite these works unless the editor has indicated otherwise.

[Note: HTML markup is below. Please do not edit.]

Reviewers' comments:

Reviewer's Responses to Questions

**Comments to the Author**

Reviewer #3: All comments have been addressed

Reviewer #4: All comments have been addressed

**********

2. Is the manuscript technically sound, and do the data support the conclusions?

Reviewer #3: Yes

Reviewer #4: Partly

**********

3. Has the statistical analysis been performed appropriately and rigorously?

Reviewer #3: Yes

Reviewer #4: I Don't Know

**********

4. Have the authors made all data underlying the findings in their manuscript fully available?

The PLOS Data policy

Reviewer #3: Yes

Reviewer #4: Yes

**********

5. Is the manuscript presented in an intelligible fashion and written in standard English?

Reviewer #3: Yes

Reviewer #4: Yes

**********

Reviewer #3: 3 June 2026

Dear Author

The revised manuscript has improved considerably and addresses most of the concerns raised during the initial review. The study investigates the repellency of selected essential oils against Anopheles arabiensis, an important malaria vector, and provides valuable information on plant-based mosquito repellents with potential applications in vector control.

The inclusion of GC-MS analysis, dose-response assessment, complete protection time evaluation, and investigation of blended oil effects strengthens the scientific merit of the work. The revised statistical analyses are more appropriate and clearly presented, and the discussion has been improved by relating the findings to existing literature.

However, a few minor issues should still be considered before publication:

1. The manuscript would benefit from additional clarification regarding the practical implications of laboratory-based protection times under field conditions.

2. The discussion of the correlation between chemical constituents and repellency should be interpreted cautiously, as correlation does not necessarily imply causation.

3. Some language and grammatical issues remain throughout the manuscript and should be corrected through careful proofreading.

4. Future studies should include skin safety/toxicity assessments and field validation to support the development of these oils as commercial repellents.

Subject to minor editorial revisions, I recommend the manuscript for publication.

Sd/

ASHISH VALA

Reviewer #4: This manuscript is about the potential of selected essential oil (EO) from different plant species and the effectiveness of these EO to repel mosquitoes. This is the second manuscript that get reviewed. Overall, the manuscript writing flow is good and easy to follow. Nevertheless, there are few issues that author needs to address.

The title can be further refined to include a complex system too such as food because later part of the manuscript interaction in a complex system was highlighted. The manuscript is written in a good flow but needs to be further improved.

The abstract needs to further improve. The current abstract too lengthy and contains unnecessary information. The detailed comment is in the manuscript or specific comment section below.

The introduction background is good. However, the author should highlight the current successful EO used commercially for insect repellant. There are many effective EO that are commercially available or proven to be a potent insect repellent such as lemon grass. The problem statement is fine. The objective needs to be clear and detail. The optimization of effective dose first determines, followed by trial on human subject.

The methodology section needs a proper explanation of the design. The sample of 5 people is not enough for scientific work. Nevertheless, the replication and repetition must be robust to support the findings. The effective dosage and the applicable dosage is two different values and not interconnected to each other. This makes the result of the effective dosage irrelevant for the application. In the discussion, the reason for these differences was due to high volatility of the compounds. Is there any measurement to measure the rate of volatility of the EO applied at the effective dosage?

The discussion is fit and explain the result. Suggest relating with other EO that known to have insect repellency such as peppermint EO, clove EO and etc.

Conclusion is concise but can be further improved. Highlight the major compounds that found in the EO extracted that potentially contribute to the repellency from mosquitoes.

Specific comment

Line 24-26; Please remove this from abstract. It should be highlight in the methodology statistical analysis.

Line 19- I don’t notice any specific pathway that been highlighted in the manuscript. Do you mean redox pathway?

Line 101-114; State how the compound was identified? which library and software used for the identification. Any internal standard used since the quantification based on area under the curve.

Line 113-114; State how the compound was identify? which library and software used for the identification. Any internal standard used since the quantification based on area under the curve.

Line 131-132; Additional for 10% too? I think this is a typing error. The 10% already mentioned. delete the words and remain the 20% only.

Line 142; state the unit as (w/v) or (v/v)

Line 145; this is very low number of samples for human test

Line 149; Please write down the formula for the calculation. Highlight this and make this as equation 1.

Line 162-169; Is this the same mosquito or a different group of mosquito? I mean the same 100 mosquito? Why this is not done simultaneously?

Line 179; state the concentration of DEET use as positive control.

Line 189-190; Please rephrase this. Do you mean mosquito landed after the 30 minutes rest?

Line 209; label this formula as equation 2

Line 218; Insert Table 1 at line 218.

Line 231 & 237; use symbol for delta & use symbol for gamma

Line 257-258; Based on the data in Table 3, they are not significant value. What are these significant values (p-values) represent? Is this comparing between ED50& ED99 or between the sample? It was not mentioned in the paragraph.

Line 286, 310, 312; Table 4, Table 5 and Table 6, Statistical indicator explanation in each table. a stand for what? significant different? They should mention different small letter mean there are statistical differences.

Line 327 & 329; Please use symbol for gamma

Line 338-340; The reference focuses on the toxicity of these compounds on human. The right paper to cite is on the effect of insects. Either this is also toxic to insect or it just repels the mosquitoes due to the smell. Suggest finding a more relevant reference.

Line 362-364; What is the difference between these approaches?

Line 642-643; Reference 53, as mentioned earlier, please find a relevant reference for the effect of toxicity on insects.

Line 664-665; In the text, this reference state EPA standard but this reference is not from EPA. This is secondary source citation. Suggest to cite both references.

**********

what does this mean?). If published, this will include your full peer review and any attached files.

If you choose “no”, your identity will remain anonymous but your review may still be made public.

**Do you want your identity to be public for this peer review?**  For information about this choice, including consent withdrawal, please see our Privacy Policy

Reviewer #3: **Yes:**  DR. ASHISHKUMAR G VALA ASSISTANT PROFESSOR

Reviewer #4: No

**********

[NOTE: If reviewer comments were submitted as an attachment file, they will be attached to this email and accessible via the submission site. Please log into your account, locate the manuscript record, and check for the action link "View Attachments". If this link does not appear, there are no attachment files.]

To ensure your figures meet our technical requirements, please review our figure guidelines: https://journals.plos.org/plosone/s/figures

You may also use PLOS’s free figure tool, NAAS, to help you prepare publication quality figures: https://journals.plos.org/plosone/s/figures#loc-tools-for-figure-preparation.

NAAS will assess whether your figures meet our technical requirements by comparing each figure against our figure specifications.

Attachment Submitted filename: Reviewer report.docx

Attachment Submitted filename: PONE-D-26-14817_R1_reviewer.pdf

# Author comment: Author response to Decision Letter 2

**DOI:** 10.1371/journal.pone.0358040.r004

6 Jul 2026

Our point-by-point response to reviewers has been uploaded as a separate file labeled 'Response to Reviewers'.

Attachment Submitted filename: Response_to_Reviewers_auresp_2.docx

# Peer review: Decision Letter 2

**DOI:** 10.1371/journal.pone.0358040.r005

**Authors:** Mansureh Ghavam

29 Jul 2026

Dear Dr. Teshome,

Thank you for submitting your manuscript to PLOS ONE. After careful consideration, we feel that it has merit but does not fully meet PLOS ONE’s publication criteria as it currently stands. Therefore, we invite you to submit a revised version of the manuscript that addresses the points raised during the review process.

Please submit your revised manuscript by Sep 12 2026 11:59PM. If you will need more time than this to complete your revisions, please reply to this message or contact the journal office at plosone@plos.org. When you're ready to submit your revision, log on to https://www.editorialmanager.com/pone/ and select the 'Submissions Needing Revision' folder to locate your manuscript file.

- A letter that responds to each point raised by the academic editor and reviewer(s). You should upload this letter as a separate file labeled 'Response to Reviewers'.
- A marked-up copy of your manuscript that highlights changes made to the original version. You should upload this as a separate file labeled 'Revised Manuscript with Track Changes'.
- An unmarked version of your revised paper without tracked changes. You should upload this as a separate file labeled 'Manuscript'.

If you would like to make changes to your financial disclosure, please include your updated statement in your cover letter. Guidelines for resubmitting your figure files are available below the reviewer comments at the end of this letter.

If applicable, we recommend that you deposit your laboratory protocols in protocols.io to enhance the reproducibility of your results. Protocols.io assigns your protocol its own identifier (DOI) so that it can be cited independently in the future. For instructions see: https://journals.plos.org/plosone/s/submission-guidelines#loc-laboratory-protocols. Additionally, PLOS ONE offers an option for publishing peer-reviewed Lab Protocol articles, which describe protocols hosted on protocols.io. Read more information on sharing protocols at https://plos.org/protocols?utm_medium=editorial-email&utm_source=authorletters&utm_campaign=protocols.

As the corresponding author, your ORCID iD is verified in the submission system and will appear in the published article. PLOS supports the use of ORCID, and we encourage all coauthors to register for an ORCID iD and use it as well. Please encourage your coauthors to verify their ORCID iD within the submission system before final acceptance, as unverified ORCID iDs will not appear in the published article. *Only*  the individual author can complete the verification step; PLOS staff *cannot*  verify ORCID iDs on behalf of authors.

We look forward to receiving your revised manuscript.

Kind regards,

Mansureh Ghavam

Academic Editor

PLOS One

Journal Requirements:

If the reviewer comments include a recommendation to cite specific previously published works, please review and evaluate these publications to determine whether they are relevant and should be cited. There is no requirement to cite these works unless the editor has indicated otherwise.

[Note: HTML markup is below. Please do not edit.]

Reviewers' comments:

Reviewer's Responses to Questions

**Comments to the Author**

Reviewer #5: All comments have been addressed

Reviewer #6: All comments have been addressed

Reviewer #7: All comments have been addressed

**********

2. Is the manuscript technically sound, and do the data support the conclusions?

Reviewer #5: Yes

Reviewer #6: Partly

Reviewer #7: Yes

**********

3. Has the statistical analysis been performed appropriately and rigorously?

Reviewer #5: Yes

Reviewer #6: No

Reviewer #7: Yes

**********

4. Have the authors made all data underlying the findings in their manuscript fully available?

The PLOS Data policy

Reviewer #5: Yes

Reviewer #6: Yes

Reviewer #7: Yes

**********

5. Is the manuscript presented in an intelligible fashion and written in standard English?

Reviewer #5: Yes

Reviewer #6: No

Reviewer #7: Yes

**********

Reviewer #5: Although the study addresses a local issue, the knowledge generated can be applied to other regions with similar characteristics. The analysis is robust and holds both local and global relevance. Additionally, the authors have successfully addressed the feedback from previous reviews. My only remaining suggestion is to polish the English phrasing

Reviewer #6: The manuscript investigates the chemical composition and mosquito repellent efficacy of essential oils extracted from Croton macrostachyus, Echinops kebericho, Eucalyptus globulus, and Juniperus procera against laboratory-reared Anopheles arabiensis. The authors characterized the essential oils using GC-MS and evaluated their repellent activities through WHO-based arm-in-cage bioassays involving human volunteers. Effective doses (ED50 and ED99), complete protection time (CPT), and the efficacy of binary oil combinations were also assessed. The topic is relevant because vector behavioral changes and increasing insecticide resistance necessitate the development of alternative mosquito control strategies. Furthermore, the investigation of Ethiopian medicinal plants represents a valuable contribution to ethnopharmacology and botanical insecticide research. The manuscript contains interesting experimental data and addresses an important public health issue. Although the manuscript has been significantly improved following the authors' revisions, several important points still require further clarification in order to enhance its scientific quality. Several methodological, analytical, statistical, and interpretative weaknesses considerably reduce the scientific robustness of the study. In its present form, several conclusions are insufficiently supported by the data.

Overall, the manuscript has scientific merit but requires substantial revision before it can be considered for publication.

Majors Comment

Line 57, The Introduction mentions insecticide resistance but does not explain why repellents remain effective despite physiological resistance mechanisms. The authors should clearly distinguish between: insecticidal resistance, behavioral avoidance, host-seeking inhibition, repellency mechanisms. This distinction is important for readers unfamiliar with vector biology.

Lines 165-170, Only five volunteers participated in the bioassays. The authors state that this sample size provides sufficient statistical power. However, no sample size calculation or power analysis is presented. This claim should either be justified statistically or removed.

Lines 187, 195-196, The cumulative dose procedure requires further explanation. Please clarify: why cumulative dosing was preferred, whether previous doses influenced subsequent responses, how evaporation between successive applications was controlled.

Lines 231-238, The authors correlate the abundance of individual compounds with repellent activity. This approach is problematic because essential oils are complex multicomponent mixtures exhibiting numerous synergistic and antagonistic interactions. Correlation alone cannot establish that individual compounds are responsible for biological activity. The Discussion should clearly acknowledge this limitation.

Lines 267-268, Statements such as « Both compounds are known to have insect repellent properties » are too strong because these compounds were not individually tested in this study. The wording should be changed to « may contribute » or « have previously been reported » instead of implying causality.

Lines 248-274, The GC-MS identification relies exclusively on NIST and Wiley spectral libraries. No Linear Retention Index (LRI) or Kovats Retention Index (RI) values are reported. For essential oil characterization, spectral matching alone is generally insufficient for reliable compound identification. The authors should report experimental retention indices together with literature values whenever possible.

Table 2, Several reported compounds appear unusual or unexpected in essential oils, including steroid-like molecules such as Pregn-16-en-20-one and Gestonorone derivatives. These identifications should be carefully verified because they may represent false-positive library matches.

Table 3, Line 286, Only ED50 and ED99 are reported. The probit regression parameters should also include: Regression slope, Confidence limits, and χ² values with interpretation.

The manuscript does not sufficiently explain why binary combinations of essential oils were investigated. The hypothesis that blending oils may improve efficacy should be supported by previous literature.

The negative control consists of ethanol. Please justify why ethanol was selected instead of another carrier commonly used in topical repellent formulations.

Dose Selection, The rationale for selecting concentrations of 10 % and 20 % should be explained. Were these concentrations based on previous studies or preliminary experiments?

Binary Mixtures, Authors evaluates binary oil combinations. Please specify: mixing ratio; preparation procedure; stability before testing; possible phase separation.

Figures 1–3, The figures present only mean repellency percentages. Please include: error bars, confidence intervals, statistical annotations.

The Discussion should acknowledge that laboratory bioassays may not accurately predict field performance because of: wind; temperature; behavioural variability.

Minors Comment

Several abbreviations are introduced before being defined.

Scientific names should be consistently italicized in all figure legends.

Several tables would benefit from reporting sample size (n).

Please verify spacing before and after percentage symbols and units. Write 10 % not 10%.

The references should be checked for consistency according to the PLOS ONE citation style.

Finally, the manuscript would benefit from careful language editing to improve clarity and correct minor grammatical and formatting inconsistencies.

Reviewer #7: Here are my comments to the authors

Title

-What is the importance of showing complex matrices on a title? Why not “Chemical composition and repellency of essential oils from four medicinal plants against Anopheles arabiensis (Diptera: Culicidae) under laboratory conditions"?

Abstract

-The abstract section should have to rewritten in a well-structured manner

-Lines 14 to 16 can be better rewritten by merging into 1 or 2 lines with a nice flow

-Lines 16 to 17 mixed objective and method; it's better to rewrite a clear objective using more appropriate words and the method followed separately.

-Line 17 against “laboratory-reared”: What is the importance of explaining it under the objective? Better to connect with the sentence under line 23.

-Lines 18 to 21: It's better to begin with "Essential oils were extracted from fresh parts of ------ using hydrodistillation."

-Line 22, “chemical composition of the EOs was characterized by GC-MS." Is "characterized" appropriate? Or analyzed using GC-MS?

-Lines 23 to 25 should have to be rewritten in a clear manner by combining separated phrases.

-Lines 25 to 28, the authors suggested rewriting the sentences by avoiding unnecessary words

-For example, about 162 compounds were identified, among which are the most abundant and showed a positive correlation ---

-Line 28, the plant parts used for EOs extraction should be stated as the method section; otherwise it makes a confusion

-Line 30: Similarly, it is better to explain “binary blends” as treatments using few words to avoid confusion.

Introduction

-Lines 38 to 42 have too much introduction and would be better if the authors rewrite the paragraph focusing on the direct study subject.

-It is recommended to rewrite the incomplete phrases with too much in-text citation

-The introduction section is recommended to improve its connectivity and flow of ideas between sentences as well as between paragraphs.

-Lines 40 to 42 can be summarized in one line by combining the ideas. For example, line 43 would be better if it begins with “Malaris is transmitted by over 70 Anopheles species." ---

-Lines 61 and 62: Is it economical to use EOs in this regard?

-Line 66: Why can't the authors use the form of smoke and tars instead of EOs?

-Lack of literature review regarding the studied plant essential oils' repellency effects and existing research gaps

-At the end of Line 68, is “both safe and biodegradable” not clear? Biodegradable for EOs?

-Lines 71 and 72, vast literature review: It is better to focus on those plants used in the current study with application method and repellent efficacy test

Materials and Methods

-It is better to write the “Essential oil extraction from plant parts” section by avoiding mixed procedures and some extra words.

-Would it be better if the authors inserted the image of plants used in this study?

-Line 110, for how long are the EOs stored before repellency tests? It is better to state the exact duration, as some active components escape due to the volatile nature of EOs.

-Line 112, “Gas chromatography/mass spectrometry (GC/MS) analysis”??? Instead, essential oil composition analysis, GC-MS profile of essential oils, or chemical composition of essential oils.

-Lines 113 to 134, Try to present only the main steps and remove extra words

-Lines 145 to 148 are not important to explain multiple times once the extracted EOs were already known. It is enough to write as “The repellency test was performed following WHO guidelines" (41). ”.

-Lines 150 to 153: try to minimize repeated similar words, which seem boring, and rewrite in clear and attractive manner

-For example, five various concentrations of EOs (1%---10%) were prepared in ethanol (v/v) or µL/mL—instead of the word "per."

-Line 154, “Additionally, a total of six binary combinations of the essential oils were created." "Formulated" can be used instead of "created."

-Line 154 to 159 can be combine and presented together

-Line 160 to 1622 can be written in one line by connecting unnecessarily separated phrases

-For repellency evaluation tests, it could be better if the authors include the image of the arm in the cage showing mosquito landing status/rate.

-What is the application method of essential oils on the hands of volunteers? Spraying/painting?

-The authors explain that the testing duration lasted for 6 hours per person, and the experiments have been conducted in three replications. Does it mean one volunteer waited for 18 hours for one treatment?

Statistical analysis

-This section is too much and mixed with calculation of some parameters; it is recommended that the authors clearly present only statistical analysis (design, testing, etc.).

Results

-Essential oil yield or essential oil content? Which is more appropriate in this context?

-Line 246, Table caption should be written correctly. For instance, Table 1. Essential oil yield (%) of five medicinal plants collected from the Gibe Valley, Ethiopia (and others better in self-explanatory ways).

-It's better to present table with the top and bottom border only

-Lines 249 to 251 could be better written in short by removing long words.

-A total of 162 compounds in which essential oils?

-The author can be designate or rename if required to better present the results of each essential oil extracted from different plants

-Lines 255 and 256: “All three of these most abundant chemical compounds derived from E. globulus have been known for insect-repellent properties." Is it important to present it here?

-Lines 257 to 258: The correlation coefficient value presented does not clearly show the association between which of the two compounds?

-Lines 262 to 263, the author better justify the finding with appropriate citation for such sentences “These 262 compounds are sesquiterpenes, and there are indications that some sesquiterpenes can exhibit repellent properties against mosquitoes and other insects," and the same is true for the sentences in lines 267 and 268 and lines 273 and 274.

-Line 275, Table 2. It would be better if the authors could present essential oil composition for each plant separately using an appropriate standard table format with a clear caption for each.

-Line 278: It's better to start with a clear presentation of results.

-Why do not the authors separately present the correlation analysis using a table rather than attaching raw data as supplementary information?

-Why is the figure not inserted at its appropriate places with their corresponding caption

In Tables 4, 5, and 6, it could be better if the authors presented the mean ± standard deviation or standard error for values labeled with significant mean letters.

-What is the main objective of using blended essential oils, and what is the basis for blending proportions?

Discussion

-Lines 356 to 358, The authors should focus on similar parameters and extraction methods employed from the previous study for discussion.

-It could be better if the authors briefly explain the testing method and conditions of those plants studied for repellent properties in previous studies.

-Lines 389 to 390: How can the type of solvent affect the efficiency of essential oils since there is no further extraction?

-Lines 390 to 394: It's better to rewrite by avoiding some repetition of similar and extra words with better flow.

-Lines 401 to 403: The authors have to clarify the drawback of using concentrated essential oils.

-Lines 427 to 429: Rewrite in better ways by aligning separated sentences and use one citation (61).

-Line 434, "A comparable result was reported by previous studies on essential oil of E. globulus in Ethiopia." What are the concentration and testing method/conditions?

-Lines 442 (end) to 445 contain similar ideas and are better revised on the basis of blending importance and consequences.

-Lines 452 to 453, “Therefore, the result implies that a 1:1 combination of most of the essential oils tested in this study did not cause a synergistic or antagonistic reaction." Is this enough evidence to conclude?

-Lines 457 to 465: The authors tried to elaborate on the use of essential oils being less effective compared to commercial repellents in many aspects. So, what is the importance of using essential oils, particularly in terms of affordability, applicability, safety, and economic feasibility?

Limitation of study

-What about the application method of essential oils (spray or other repellency testing methods (if any)?

-Advanced essential oil formulation (macro and nano encapsulation) for better applicability?

-Economic feasibility analysis?

Conclusion

-The conclusion is full of recommendation and can be better improved

**********

what does this mean?). If published, this will include your full peer review and any attached files.

If you choose “no”, your identity will remain anonymous but your review may still be made public.

**Do you want your identity to be public for this peer review?**  For information about this choice, including consent withdrawal, please see our Privacy Policy

Reviewer #5: No

Reviewer #6: No

Reviewer #7: No

**********

[NOTE: If reviewer comments were submitted as an attachment file, they will be attached to this email and accessible via the submission site. Please log into your account, locate the manuscript record, and check for the action link "View Attachments". If this link does not appear, there are no attachment files.]

To ensure your figures meet our technical requirements, please review our figure guidelines: https://journals.plos.org/plosone/s/figures

You may also use PLOS’s free figure tool, NAAS, to help you prepare publication quality figures: https://journals.plos.org/plosone/s/figures#loc-tools-for-figure-preparation.

NAAS will assess whether your figures meet our technical requirements by comparing each figure against our figure specifications.

# Author comment: Author response to Decision Letter 3

**DOI:** 10.1371/journal.pone.0358040.r006

19 Aug 2026

A point-by-point response is given to each point raised by the academic editor and reviewer(s) and has been uploaded as a separate file labeled "Response to Reviewers".

Attachment Submitted filename: Response_to_Reviewers_auresp_3.docx

# Editor report: Decision Letter 3

**DOI:** 10.1371/journal.pone.0358040.r007

**Authors:** Mansureh Ghavam

26 Aug 2026

Repellency and chemical composition of essential oils from four medicinal plants against Anopheles arabiensis (Diptera: Culicidae) under laboratory conditions

PONE-D-26-14817R3

Dear Dr. Teshome,

We’re pleased to inform you that your manuscript has been judged scientifically suitable for publication and will be formally accepted for publication once it meets all outstanding technical requirements.

Within one week, you’ll receive an e-mail detailing the required amendments. When these have been addressed, you’ll receive a formal acceptance letter and your manuscript will be scheduled for publication.

An invoice will be generated when your article is formally accepted. Please note, if your institution has a publishing partnership with PLOS and your article meets the relevant criteria, all or part of your publication costs will be covered. Please make sure your user information is up-to-date by logging into Editorial Manager at Editorial Manager® and clicking the ‘Update My Information' link at the top of the page. For questions related to billing, please contact billing support.

If your institution or institutions have a press office, please notify them about your upcoming paper to help maximize its impact. If they’ll be preparing press materials, please inform our press team as soon as possible -- no later than 48 hours after receiving the formal acceptance. Your manuscript will remain under strict press embargo until 2 pm Eastern Time on the date of publication. For more information, please contact onepress@plos.org.

Kind regards,

Mansureh Ghavam

Academic Editor

PLOS One

Additional Editor Comments (optional):

Reviewers' comments:

# Editor report: Acceptance letter

**DOI:** 10.1371/journal.pone.0358040.r008

**Authors:** Mansureh Ghavam

PONE-D-26-14817R3

PLOS One

Dear Dr. Teshome,

I'm pleased to inform you that your manuscript has been deemed suitable for publication in PLOS One. Congratulations! Your manuscript is now being handed over to our production team.

At this stage, our production department will prepare your paper for publication. This includes ensuring the following:

* All references, tables, and figures are properly cited

* All relevant supporting information is included in the manuscript submission,

* There are no issues that prevent the paper from being properly typeset

You will receive further instructions from the production team, including instructions on how to review your proof when it is ready. Please keep in mind that we are working through a large volume of accepted articles, so please give us a few days to review your paper and let you know the next and final steps.

Lastly, if your institution or institutions have a press office, please let them know about your upcoming paper now to help maximize its impact. If they'll be preparing press materials, please inform our press team within the next 48 hours. Your manuscript will remain under strict press embargo until 2 pm Eastern Time on the date of publication. For more information, please contact onepress@plos.org.

You will receive an invoice from PLOS for your publication fee after your manuscript has reached the completed accept phase. If you receive an email requesting payment before acceptance or for any other service, this may be a phishing scheme. Learn how to identify phishing emails and protect your accounts at https://explore.plos.org/phishing.

If we can help with anything else, please email us at customercare@plos.org.

Thank you for submitting your work to PLOS One and supporting open access.

Kind regards,

PLOS One Editorial Office Staff

on behalf of

Dr. Mansureh Ghavam

Academic Editor

PLOS One
